# Long-range mutual activation establishes Rho and Rac polarity during cell migration

**DOI:** 10.1038/s41556-026-01965-1

**Published:** 2026-06-10

**Authors:** Henry De Belly, Andreu F. Gallén, Evelyn Strickland, Dorothy C. Estrada, David Sanchez Godinez, Eric Neiva, Patrick J. Zager, Tamas L. Nagy, Janis K. Burkhardt, Hervé Turlier, Orion D. Weiner

**Affiliations:** 1 Cardiovascular Research Institute, University of California, San Francisco, San Francisco, CA USA; 2 Department of Biochemistry and Biophysics, University of California, San Francisco, San Francisco, CA USA; 3 Center for Interdisciplinary Research in Biology (CIRB), Collège de France, CNRS, INSERM, Université PSL, Paris, France; 4 Department of Pathology and Laboratory Medicine, Children’s Hospital of Philadelphia Research Institute and Perelman School of Medicine, University of Pennsylvania, Philadelphia, PA USA; 5 Present Address: Children’s Medical Center Research Institute, University of Texas Southwestern Medical Center, Dallas, TX USA; 6 Present Address: Immunology Program, Memorial Sloan Kettering Cancer Center, New York, NY USA; 7 Present Address: Department of Fluid Mechanics, Universitat Politècnica de Catalunya - Barcelona Tech (UPC), Barcelona, Spain; 8 Present Address: Broad Stem Cell Research Center, University of California, Los Angeles, Los Angeles, CA USA; 9 Present Address: Center for Integrative Biology (CBI), University of Toulouse, CNRS, Toulouse, France

**Keywords:** Cell polarity, Chemotaxis, Membrane biophysics, Actin

## Abstract

In migrating cells, the GTPase Rac organizes a protrusive front, whereas Rho organizes a contractile back. How these GTPases are positioned at opposite poles remains unclear. We leverage optogenetics, mechanical perturbations, and mathematical modelling to reveal a surprising mechanochemical long-range mutual activation between front and back polarity programmes that complements their well-known local mutual inhibition. Rac-based protrusions elevate membrane tension, stimulating an mTORC2-dependent activation of Rho at the opposite side of the cell. Conversely, Rho-mediated contractility induces cortical-flow-based regulation of phosphoinositide signalling that triggers Rac activation distally. We develop a minimal mechanochemical model to explain how long-range facilitation, together with local inhibition, enables robust Rho and Rac partitioning. Our findings demonstrate how the actin cortex and plasma membrane interact as an integrated mechanochemical system for long-range Rac–Rho patterning. This circuit is required for efficient polarity and migration in primary human T cells and is conserved in epithelial cells, highlighting the generality of this mechanism.

## Abstract (web summary)

De Belly et al. show Rac induces membrane-tension-mediated mTORC2 activation to stimulate long-range Rho activation. Meanwhile, Rho-mediated contractility and blebbing at the opposite side of the cell may lead to PI3K-dependent Rac activation.

## Main

**Fig. 1:**
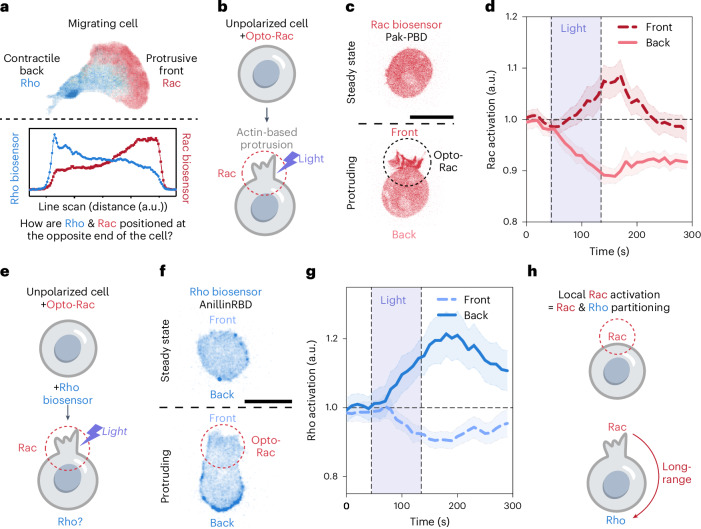
Local Rac activation stimulates long-range RhoA activation at the opposite side of the cell. **a**, Confocal image and associated line-scan of a migrating neutrophil-like HL-60 cell expressing the polarity biosensors for Rac (Pak-PBD) and Rho (AnillinRBD). In migrating cells, Rac and Rho localize to the protruding cell front and contracting cell back, respectively. Here we sought to investigate how Rac and Rho are appropriately positioned at the opposite poles of a migrating cell. **b**, We locally activated the front polarity programme Rac in an initially unpolarized neutrophil-like HL-60 cell using optogenetics (opto-PI3K, Methods). **c**, Time-lapse confocal images of an unpolarized cell before and during opto-Rac stimulation. Rac activation was monitored via the Rac biosensor Pak-PBD. **d**, Average time trace of Rac activation at the plasma membrane at the site of opto-Rac activation (cell front) compared with the opposite side of the cell (cell back) (mean ± 95% CI; *n* = 29, *N* = 3). **e**, We locally activated Rac in an initially unpolarized cell while simultaneously measuring Rho activation using AnillinRBD. **f**, Time-lapse confocal images of an unpolarized cell before and during opto-Rac stimulation. Rho activation was monitored using the biosensor AnillinRBD. **g**, Average time trace of Rho activation at the plasma membrane for the front versus back of the cell following opto-Rac activation. (mean ± 95% CI; *n* = 43, *N* = 8). **h**, Local Rac activation leads to long-range activation of Rho at the opposite side of the cell. Scale bars, 10 μm. Unless otherwise indicated, for all panels, *n*, individual cell replicate; *N*, biological replicates.

For proper physiology, many cells need to polarize or restrict different signalling programmes to different portions of their cell surface. Rac and Rho GTPases are key regulators of cell polarity and spatially pattern the actin cytoskeleton for mitosis, morphogenesis, migration and development^1^. Rac is localized to the front of migrating cells, where it promotes the extension of sheet-like lamellipodia or pressure-driven blebs (Fig. 1a)^2,3^. Rho is localized to the back of migrating cells, where it promotes local myosin-2-based contractions and long-range cortical flows^1,4^ (Fig. 1a).

While we know that Rho and Rac mutually inhibit each other locally^1,5–7^, how they are properly positioned at opposite ends of the cell during migration remains poorly understood.

Most models of polarity in migration are based on local biochemical interactions^6,8–15^, with a majority of mathematical models of polarity establishment relying on the so-called wave-pinning mechanism^16–19^, where a travelling front of active Rho GTPase is pinned by a limited bath of cytosolic inactive form. Such a mechanism relies on the fast diffusion of the inactive form and a hypothesis of fixed total protein amount. However, previous studies have challenged these requirements, demonstrating that a diffusion-based inhibitor or depletion mechanism is not sufficient to explain neutrophil polarization^20–23^. In addition, local inhibition by itself is also not sufficient to explain the long-range partitioning of active pools of Rho and Rac and to coordinate front and back at the scale of a migrating cell (Supplementary Note, sections 1–3). The requirement for an additional long-range communication had long been suggested^20–25^, but direct evidence and a molecular mechanism for this link is unknown.

Cell polarity involves long-range cellular information processing and necessitates information flow at the cellular scale. Forces transmitted via the actin cortex and the plasma membrane have emerged as key conduits for this global coordination. In migration, membrane tension guides shape by relaying actin-based protrusive forces at the front to the contraction of the rear, setting up a global competition that enables the establishment of a single dominant front^26–31^. Here we investigate whether the membrane and cortex could act as the mechanical conduit for long-range coordination of the front and back polarity programmes.

To probe the long-range spatial coordination of Rac and Rho, we take advantage of optogenetic activators of Rac and Rho in initially unpolarized cells. This enables us to precisely and specifically activate either Rac or Rho in a small region of the cell and observe the global response of the other GTPase. By combining optogenetics with manipulation of cell mechanics and mathematical modelling, we find that both the front and back mutually activate each other at a distance, and this occurs using two distinct pathways. The front stimulates the back via membrane tension, whereas the back stimulates the front via cortical remodelling. We demonstrate the physiological relevance of our findings for immune cell migration using primary human T cells. Our results demonstrate that the actin cortex and plasma membrane act as an integrated mechanochemical system to ensure the proper positioning of the front and back polarity programmes during cell migration.

## Results

### Local Rac-based protrusions stimulate long-range Rho activation at the opposite side of the cell

**Extended Data Fig. 1:**
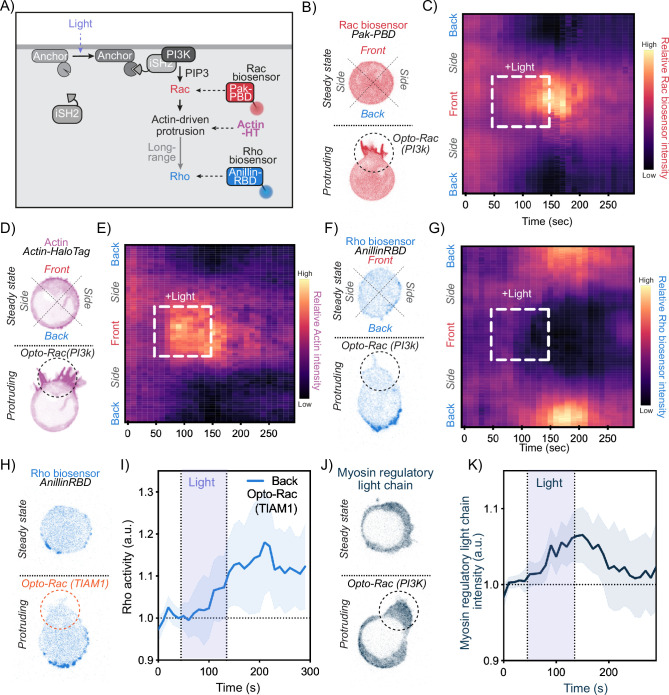
Optogenetic control of both PI3K and Rac triggers localized actin-driven cell protrusion and long-range Rho and Myosin II activation at the opposite side of the cell. (**A**) Schematic of local Rac activation via opto-PI3K. When localized 488-nm light is applied, the membrane anchor protein (iLid-BFP-CAAX) changes conformation, which leads to the binding of inter SH2 domain (iSH2) to the illuminated region. iSH2 then recruits PI3K, whose lipid product (PIP_3_) induces the activation of Rac GTPase (Rac). We monitor Rac activation using the Rac biosensor Pak-PBD-mCherry, which recognizes and binds the active GTP-bound Rac. Rac activation via opto-PI3K leads to localized actin-mediated protrusion, which we monitor by expression of Actin-HaloTag. Long-range Rho activation by local Rac activation is monitored using the Rho Biosensor AnillinRBD, which recognizes the active GTP-bound Rho (preferentially RhoA). (**B**) Time-lapse confocal images of an unpolarized cell before and during opto-Rac stimulation. Rac activation was monitored via the Rac biosensor Pak-PBD. (**C**) Kymograph of Rac activation (Pak-PBD intensity) along the normalized cell circumference (y axis) showing that over time (x axis), Rac activation increases at the front in response to opto-PI3K. (N = 3, *n* = 42). (**D**) Time-lapse confocal images of an unpolarized cell expressing actin-HaloTag before and during opto-Rac stimulation. (**E**) Kymograph of actin intensity during opto-PI3k activation (N = 2, n = 17). (**F**) Time-lapse confocal images of an unpolarized cell expressing AnillinRBD–mCherry before and during opto-Rac stimulation. (**G**) Kymograph of Rho activation in response to local Rac activation (opto-PI3k) (N = 8, n = 43). (**H**) Time-lapse confocal images of an unpolarized cell before and during opto-Rac (TIAM1) stimulation. Rho activation was monitored using the biosensor AnillinRBD. (**I**) Average time trace of Rho activation at the plasma membrane for the front versus back of the cell following opto-Rac (TIAM1) activation showing that direct opto-Rac activation leads to long-range Rho activation (mean ± 95%CI; n = 16, N = 2). (**J**) Time-lapse confocal images of an unpolarized cell. Myosin II activity was monitored using the biosensor pRLC showing that local Rac activation leads to myosin activation at the opposite side of the cell. (**K**) Average time trace of pRLC level at the plasma membrane for the back of cells following opto-Rac (PI3K) activation. (mean ± 95%CI; n = 15, N = 2). For all panels n = individual cell replicate, N = biological replicate.

To investigate how the front and back polarity programmes are properly positioned at the opposite ends of the cell, we first analysed the establishment of cell polarity in a context where we can independently control the spatial and temporal dynamics of each of these programmes. Towards this end, we leveraged an optogenetic approach (via local activation of PI3K^32^; Methods), to locally activate the front polarity programme Rac in initially unpolarized neutrophil-like HL-60 cells (Extended Data Fig. 1a). As previously demonstrated, this local Rac activation leads to actin-driven protrusions like those seen at the cell front during migration (Extended Data Fig. 1)^31,32^. Rac activation, visualized with Pak-PBD (P21-activated kinase 1 p21-binding domain), is locally enriched in the zone of activation (cell front), while being depleted at the opposite side of the cell (cell back) (Fig. 1b–d, Extended Data Fig. 1b,c and Supplementary Video 1). These results confirm that opto-PI3K locally activates Rac. Next, we sought to investigate how this local zone of Rac activation influences the back polarity programme. To this end, we used local Rac activation via either opto-PI3K or via direct Rac1/2 activation using Opto-TIAM1 (ref. ^33^) while monitoring the signalling activity of Rho (likely RhoA) using the biosensor AnillinRBD (which preferentially binds to active RhoA) (Fig. 1e and Supplementary Video 2). In both cases light-induced Rac activation elicits a rapid long-range increase in Rho activation at the opposite side of the cell (Fig. 1f,g and Extended Data Fig. 1f–i). To assay the effectors of contractility that act downstream of Rho activation, we analysed the dynamics of a fluorescently tagged myosin regulatory light chain and found it to parallel the increase in Rho activation in response to light-induced Rac activation at the other end of the cell (Extended Data Fig. 1j,k).

**Extended Data Fig. 2:**
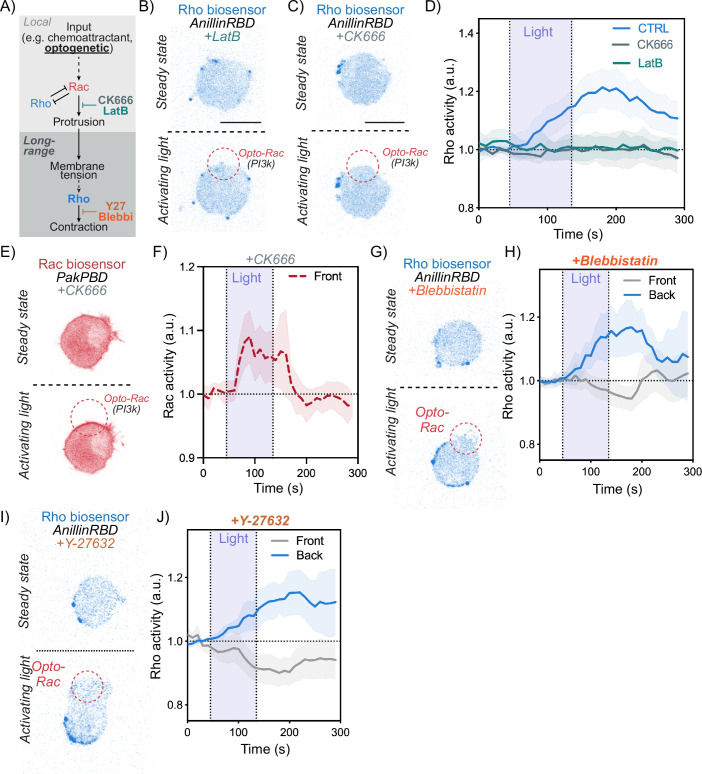
Long-range Rho activation requires cellular protrusion but not contraction. (**A**) To test the role of protrusions in Rac/Rho crosstalk, we locally activated Rac via optogenetics in the presence of inhibitors of actin assembly (Latrunculin B) or the Arp2/3 complex activation (CK666). (**B**)(**C**) Time-lapse confocal images of unpolarized cells expressing AnillinRBD–mCherry before and during opto-Rac stimulation treated with either 10 μM of Latrunculin B (**B**) or 100 μM of CK666 (**C**).(**D**) Average time trace of Rho activation at the plasma membrane at the back of cells treated with either 10 μM of Latrunculin B or 100 μM of CK666 demonstrate minimal change in Rho activation following opto-Rac activation (mean ± 95%CI; n = 20 for CK666, n = 30 for LatB, N = 3) in contrast to control cells in which the cytoskeleton was not inhibited (same curve as in Fig. 1d). (**E**) Time-lapse confocal images of an unpolarized cell expressing the Rac biosensor Pak-PBD before and during opto-Rac stimulation treated with 100 μM of CK666. These experiments show that preventing cellular protrusion does not impair Rac activation in response to opto-PI3K. (**F**) Average time trace of Rho activation at the plasma membrane at the back of cells treated 100 μM of CK666 (mean ± 95%CI; n = 20, N = 2). (**G**) Confocal images of opto-PI3K cells expressing the Rho biosensor AnillinRBD treated with 10μM of para-amino-blebbistatin (Myosin inhibitor) before and during light-induced protrusions. (**H**) Average time trace of Rho activation at the plasma membrane at the back of cells treated with 10 μM of para-amino-blebbistatin demonstrate that myosin contractility is not required for the protrusion-mediated long-range Rho activation (mean ± 95%CI; n = 20, N = 2) (**I**)(**J**) Same as (**G**)(**H**) but using cells treated with 20μM of the ROCK inhibitor Y-27632, showing that contractility is not required for the protrusion-mediated long-range Rho activation (mean ± 95%CI; n = 15, N = 2). Unless indicated otherwise, for all panels, n = individual cell replicate, N = biological replicates.

We next sought to understand how local Rac activation in one part of the cell triggers Rho activation at the opposite end. We considered two possibilities: distal Rho activation could result from local Rac-based biochemical inhibition^1^ or indirect long-range mechanical signals. One possible means of long-range Rho activation would be Rac-induced protrusions, which generate a membrane tension increase that propagates across the cell^31^ (Extended Data Fig. 2a). To distinguish between these possibilities, we impaired Rac-mediated protrusion formation by applying pharmacological inhibitors of actin assembly (latrunculin) or Arp2/3 complex activation (CK666) to opto-PI3K cells (Extended Data Fig. 2a–d). While these perturbations do not impair our ability to optogenetically activate Rac (Extended Data Fig. 2e,f), they inhibited Rac-mediated Rho activation at the other end of the cell. To test whether Rho activation depends on the actomyosin network, we impaired actomyosin contractility by treating cells with either the myosin inhibitor blebbistatin or the ROCK inhibitor Y-27632 which impairs cellular contraction without affecting the ability of cells to protrude and to generate long-range tension increase, and observed no noticeable effect on Rho as a result of local Rac activation (Extended Data Fig. 2g–j). These data indicate that protrusion-mediated Rho activation is independent of contractility. Altogether, our results support long-range activation of Rho by Rac, operating independently from the well-established local antagonism between these GTPases (Fig. 1h).

### Rac stimulates long-range Rho activation via membrane-tension-mediated mTORC2 activation

**Fig. 2:**
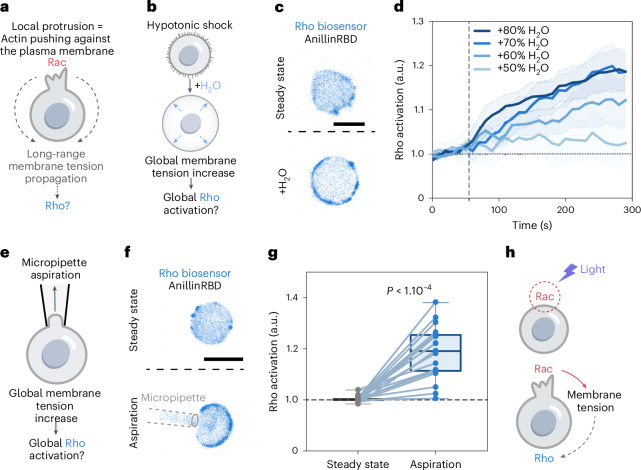
Rac acts through protrusion-mediated increases in membrane tension for long-range activation of Rho. **a**, Local Rac-mediated cell protrusion leads to a global increase in membrane tension^31^. **b**, If protrusions activate Rho by elevating membrane tension, then increasing membrane tension (even in the absence of Rac activation) should suffice to activate Rho. Towards this end, we used hypotonic shock to elevate membrane tension. **c**, Time-lapse confocal images of an unpolarized opto-Rac HL-60 cell expressing the Rho biosensor (AnillinRBD) before and after hypotonic shock. **d**, Average time trace of Rho activation (as measured by AnillinRBD) at the plasma membrane in response to hypotonic shocks of various intensity (*N* = 3, *n* = 20 for 50%, *n* = 21 for 60%, *n* = 31 for 70% and *n* = 27 for 80%, mean ± 95% CI). Hypotonic-shock-based elevation of membrane tension suffices to globally increase RhoA activation. **e**, As an alternate approach to increase membrane tension, we leveraged micropipette aspiration^31^. **f**, Time-lapse confocal images of an unpolarized opto-Rac HL-60 cell expressing the Rho biosensor (AnillinRBD) before and after micropipette aspiration (Methods). **g**, Average Rho activation (as measured by AnillinRBD) before (steady state, normalized to first time point) and during aspiration. Micropipette-based elevation of membrane tension stimulates Rho activation. Boxes and whiskers show median and min to max (*N* = 3, *n* = 19; *P* values from a Wilcoxon paired Student’s *t-*test). **h**, Membrane tension increase by local Rac activation stimulates long-range Rho activation. Scale bars, 10 μm. For all panels, *n*, individual cell replicate, *N*, biological replicates.

**Extended Data Fig. 3:**
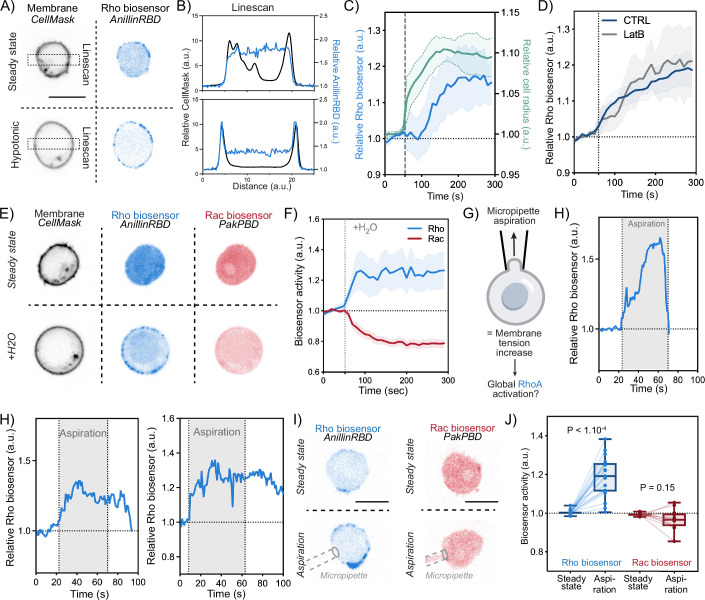
Rho, unlike Rac, is activated by increased membrane tension. (**A**) Confocal images of a cell before and during osmotic shock ( + 70% water) expressing the Rho biosensor AnillinRBD and stained with the plasma membrane marker CellMask. (**B**) line-scan of the Rho and membrane intensity before and during osmotic shock, showing a decrease in background biosensor in the cytoplasm (cytoplasmic dilution) and a recruitment of the biosensor at the plasma membrane. (**C**) Left y axis, average time trace of Rho activation at the membrane in response to hypotonic shock (70% water). Right y axis, cell radius over time. (N = 2, n = 11, means ± 95%CI) (**D**) Average time trace of Rho activation at the membrane in response to hypotonic shock (80% water) for control cells or cells treated with 10 μM of Latrunculin B, showing that actin inhibition doesn’t impair Rho recruitment in response to elevated membrane tension. (data for control is the same as Fig. 2d, For LatB data N = 3, n = 16, means ± 95%CI). (**E**) Confocal images of a cell dyed with the membrane marker CellMask and expressing both Rho (AnillinRBD) and Rac (Pak-BPD) biosensors before and during osmotic shock ( + 70% water) (**F**) Average time trace of Rho and Rac activation at the plasma before and after hypo-osmotic shock (60 mOsm or 70% H2O). Hypotonic-shock-based elevation of membrane tension suffices to globally increase RhoA activation and leads to a global decrease in Rac activation as was previously reported^28^ (mean ± 95%CI; n = 28, N = 3). (**G**) As an alternate approach to increase membrane tension, we leveraged micropipette aspiration^31^ (**H**) Three example time trace of Rho activation at the plasma membrane in response to micropipette aspiration. (**I**) Time-lapse confocal images of an unpolarized opto-Rac HL-60 cell expressing either a Rho biosensor (AnillinRBD) or a Rac biosensor (Pak-PBD) before and after micropipette aspiration. (**J**) Average Rho and Rac activation before (steady state) and during aspiration. Micropipette-based elevation of membrane tension markedly stimulates Rho activation while leading to a little change in Rac activation. Data for AnillinRBD is the same as Fig. 2g. Box and whiskers: median and min to max; p values from Wilcoxon paired Student’s t test. Scale bars: 10μm. For all panels, n = individual cell replicate, N = biological replicates.

If protrusions stimulate Rho activation through an increase in membrane tension^31^ (Fig. 2a), membrane tension increases should suffice to stimulate Rho activation even in the absence of Rac activation or protrusions. To investigate this possibility, we used hypotonic shock to stimulate an increase in membrane tension^34^ (Fig. 2b). Hypotonic shock induced a rapid and global increase in Rho activation across the cell (Fig. 2c,d, Extended Data Fig. 3a–f and Supplementary Video 3). To verify that this increase in Rho signalling activity is a function of increased membrane tension and not a secondary consequence of the rebuilding of the actin cortex following hypotonic shock, we combined our hypotonic shock assay with latrunculin B treatment, a combination that potently depolymerizes the actin cortex^31,34^. Under these conditions, osmotic shock still activates Rho, indicating that the actin cytoskeleton is not necessary for membrane-stretch-based Rho activation (Extended Data Fig. 3d). As an alternative approach, we next used a micropipette-based aspiration assay, which we previously demonstrated to increase membrane tension^31^. This mechanical manipulation also stimulates global Rho activation (Fig. 2e–g, Extended Data Fig. 3g–j and Supplementary Video 4). The activation of Rho by hypotonic shock, micropipette aspiration and opto-induced protrusions likely reflects a role for membrane tension as a Rho activator. To test whether increases in tension also affect Rac activation, we performed measurements of Rac activation in response to both osmotic shock and micropipette aspiration and found that, as previously reported, elevated tension does not activate Rac (Extended Data Fig. 3e,f,i,j). Our data show that Rac acts through protrusion-mediated increases in membrane tension to stimulate Rho at a distance.

**Fig. 3:**
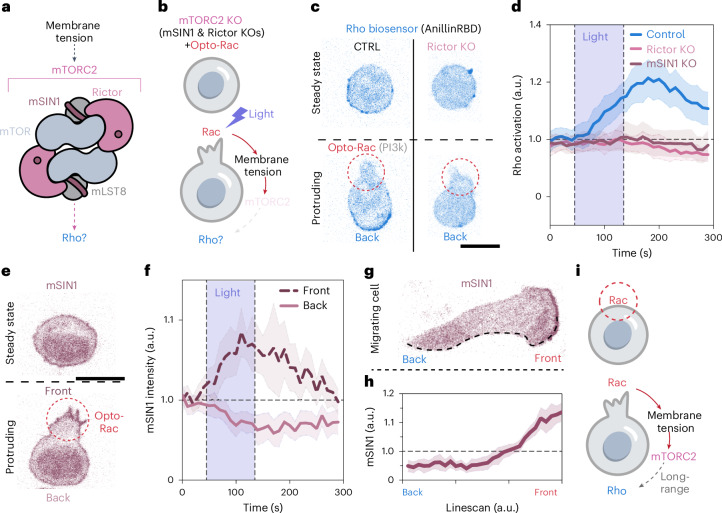
mTORC2 links membrane tension increases to Rho activation. **a**, We investigate whether mTORC2 is part of the mechanosensory pathway that links increases in membrane tension to the activation of Rho. **b**, We used optogenetics to locally activate Rac in control cells versus cells lacking different core components of the mTORC2 complex (Rictor or mSIN1 knockouts). **c**, Time-lapse confocal images of an unpolarized control or Rictor KO cell before and during opto-Rac stimulation. Rac activation was monitored via the Rac biosensor Pak-PBD. **d**, Local Rac activation potently stimulates long-range activation of Rho in control cells (blue, same data as Fig. 1g) but not cells deficient in mTORC2 activity (Rictor KO and mSIN1 KO) (mean ± 95% CI; *n* = 18 for Rictor KO, *n* = 25 for mSIN1 KO, *N* = 3). Control curve is same as in Fig. 1d. **e**, Time-lapse confocal images of an unpolarized cell expressing the mTORC2 subunit mSIN1 before and during opto-Rac stimulation showing mSIN1 enrichment at the protruding front. **f**, Average time trace of mSIN1 level at the plasma membrane at the site of opto-Rac activation (cell front; dotted line) compared with the opposite side of the cell (cell back; solid line) (mean ± 95% CI; *n* = 20, *N* = 3). **g**, Confocal images of a migrating neutrophil-like HL-60 cell expressing mSIN1. **h**, Average line-scan of mSIN1 level across migrating HL-60s, showing enrichment of mTORC2 at the cell front (mean ± 95% CI; *n* = 13, *N* = 2). **i**, Local Rac activation stimulates long-range Rho activation via membrane-tension-mediated mTORC2 activation. For all panels, *n*, individual cell replicate; *N*, biological replicates.

**Extended Data Fig. 4:**
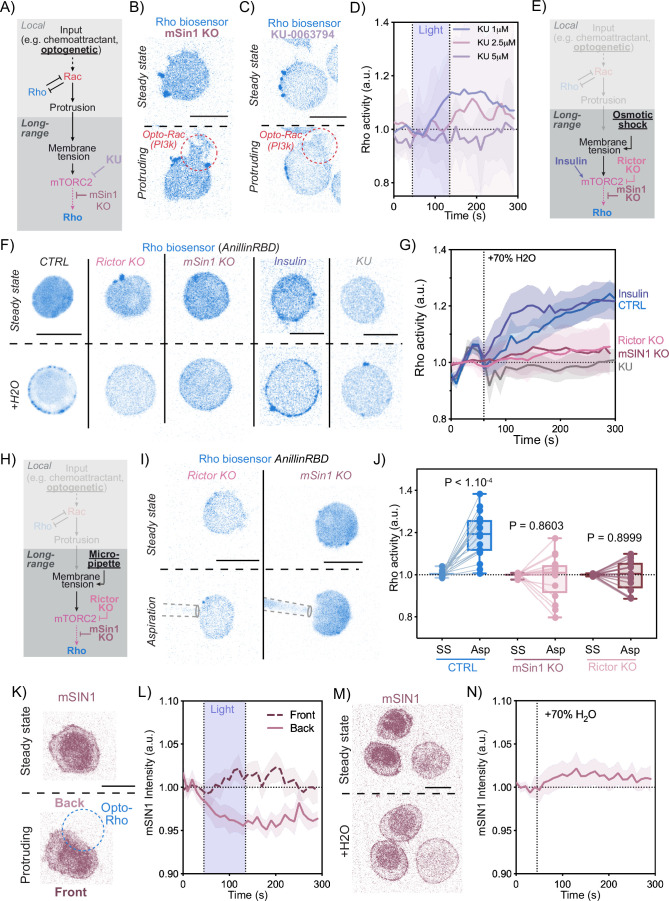
mTORC2 links membrane tension increases to Rho activation. We investigate whether mTORC2 is part of the mechanosensory pathway that links increased in membrane tension to the activation of Rho. (**A**) We used optogenetics to locally activate Rac in control cells versus cells with impaired mTORC2 activity (either by using cells lacking mSIN1, a core component of the mTORC2 complex or cells treated with the mTOR inhibitor KU-0063794. (**B**) Time-lapse confocal images of an unpolarized control or mSIN1 KO cell before and during opto-Rac stimulation. Rac activation was monitored via the Rac biosensor Pak-PBD. (**C**) Time-lapse confocal images of unpolarized cells expressing AnillinRBD–mCherry treated with 5uM of KU-0063794 before and during opto-Rac stimulation. (**D**) Average time trace of Rho activation in opto-Rac cells treated with either 1uM, 2.5uM or 5uM of KU-0063794. (N = 2, n = 10 for 1uM, n = 8 for 2.5uM, n = 7 for 5uM). (**E**)(**F**) Osmotic shock to globally increase membrane tension in cells lacking core components of the mTORC2 complex (either Rictor or mSIN1 knockouts), pharmacologically inhibited with mTOR inhibitor KU-0063794, or stimulated with the mTORC2 activator Insulin. (**G**) Time-lapse confocal images of unpolarized control, Rictor KO, mSIN1 KO cells or cells treated with 10 μM of the mTOR inhibitor KU-0063794, or with Insulin (10 μg ml) expressing AnillinRBD–mCherry before and during hypotonic shock (70% Water). (**H**) Average time trace of Rho activation at the plasma membrane before and after hypo-osmotic shock in control demonstrate a requirement for mTORC2 in membrane-tension-mediated activation of RhoA (mean ± 95%CI; KU n = 50, CTRL n = 55, Insulin = 48, Rictor KO n = 35, mSIN1 KO n = 34, N = 3). (**H**) Same as (**E**) but using micropipette aspiration to mechanically stimulate an increase in membrane tension. (**I**) Time-lapse confocal images of unpolarized Rictor KO or mSIN1 KO HL-60s cells expressing the Rho biosensor (AnillinRBD) before and after micropipette aspiration. (**J**) Average Rho activation before (steady state) and during aspiration of control, Rictor KO and mSIN1 KO cells. Values for control are the same as Fig. 2g. Box and whiskers: median and min to max; p values from Wilcoxon paired Student’s t test. (**K**) Time-lapse confocal images of an unpolarized cell expressing the mTORC2 subunit mSIN1 before and during opto-Rho stimulation showing mSIN1 depletion at the contractile back. (**L**) Average time trace of mSIN1 level at the plasma membrane at the site of opto-Rho activation (cell back; solid line) compared to the opposite side of the cell (cell front; dotted line) (mean ± 95%CI; n = 20, N = 3). (**M**) Time-lapse confocal images of unpolarized cell expressing mSIN1-mCherry before and during hypotonic shock (70% Water). (**N**) Average time trace of mSIN1 level at the plasma membrane before and after hypo-osmotic shock (mean ± 95%CI; n = 30, N = 2). Scale bars: 10μm. n = individual cell replicate, N = biological replicate. For all panels, n = individual cell replicate, N = biological replicates.

Next, we sought to identify the mechanosensors that link increases in membrane tension to Rho activation. The mTORC2 complex emerged as a strong candidate, given its responsiveness to membrane tension and its key roles in regulating cell polarity and motility^28,29,34–36^(Fig. 3a). To test whether mTORC2 links membrane tension increases to Rho activation, we performed our opto-Rac activation assay in cells lacking different core components of the mTORC2 complex (Rictor or mSIN1 CRISPR knockout (KO) cell lines)^29^ or using a pharmacological inhibition of mTOR. Cells deficient in mTORC2 (Rictor or mSIN1 KO) or treated with mTOR inhibitor KU-0063794 failed to activate Rho upon local Rac activation (Fig. 3b–d, Extended Data Fig. 4a–d and Supplementary Video 5) despite retaining their ability to generate protrusions. To further isolate the tension-mediated potentiation of Rho activation, we repeated previous hypotonic and micropipette aspiration assays in mTORC2-impaired cells. Our results confirmed that mTORC2 is essential for Rho activation following both hypotonic shock (Extended Data Fig. 4e–g) and micropipette aspiration (Extended Data Fig. 4h–j). As an alternative means to demonstrate mTORC2 involvement beyond the genetic and pharmacological inhibition of mTORC2, we used insulin to potentiate mTORC2 activation; this resulted in increased Rho activation following membrane stretch (Extended Data Fig. 4f–g).

We next examined the localization and dynamics of mTORC2 during cell polarization. To this end, we tagged the mTORC2-specific subunit mSIN1, as previously described in HeLa cells^37^, and monitored mTORC2 localization in cells subjected to opto-Rac stimulation. Light-induced Rac activation elicits mTORC2 enrichment at the cell front and a concomitant depletion from the cell rear (Fig. 3e,f). In addition, we used an optogenetic approach to locally activate the back polarity programme Rho (via LARG; Methods) while simultaneously monitoring mSIN1 and found that mTORC2 decreases at the site of contraction (Extended Data Fig. 4k,l). Consistent with this pattern, mTORC2 is absent from the back of migrating cells while being enriched at the leading edge and at the plasma membrane of osmotically swollen cells (Fig. 3g,h and Extended Data Fig. 4m,n). If this redistribution reflects enhanced mTORC2 activity at sites of elevated membrane tension, it implies the existence of a diffusible downstream effector that transmits mTORC2-dependent signals from protrusive regions to distal cellular compartments, thereby enabling Rho activation at the cell rear, analogous to previously described long-range signalling mechanisms in *Dictyostelium*^38,39^ that link Akt activation at the leading edge with Paka phosphorylation at the trailing edge (Discussion). Our work reveals a molecular pathway in which Rac-induced actin protrusions globally increase membrane tension, activating the mechanosensitive mTORC2, which in turn stimulates Rho activation **(**Fig. 3i**)**.

### Local Rho activation leads to Rac activation at the opposite side of the cell

We previously established that local Rac-mediated protrusion is sufficient for long-range Rac and Rho partitioning across the cell. However, not all migrating cells generate branched actin-based protrusions at their front. In bleb-based migration, Rho-mediated contraction at the cell back is the primary force generator, producing increased intracellular pressure leading to blebbing on the opposite side^2,40^. Interestingly, while both blebbing programmes and actin polymerization-based protrusions use different protrusion engines, both rely on the same Rho-Rac polarity machinery^41,42^. Given the role of Rho activation in promoting bleb-based migration, we next wondered whether local Rho activation is also sufficient to trigger long-range Rac and Rho partitioning.

**Fig. 4:**
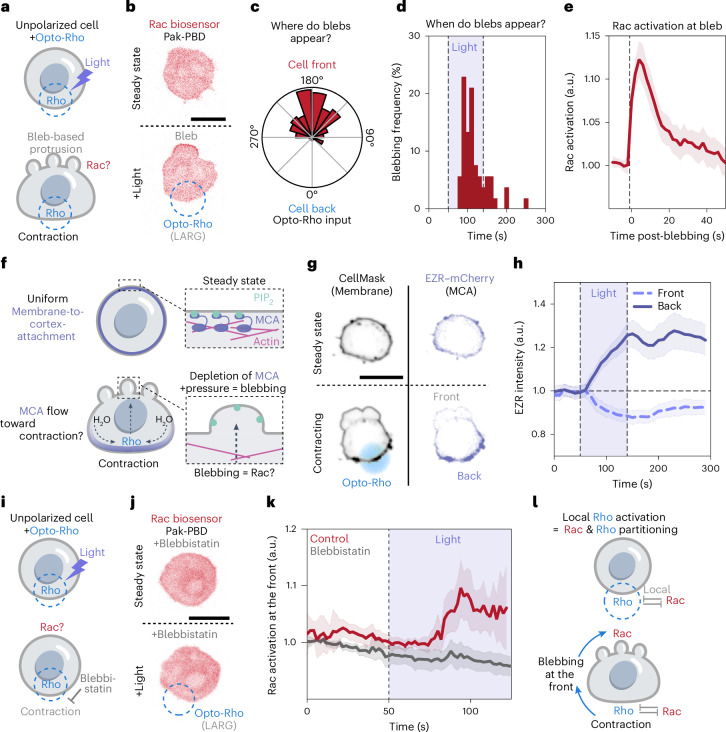
Local Rho activation elicits Rac activation at the opposite side of the cell. **a**, Local Rac activation leads to long-range Rho activation at the opposite end of the cell via membrane tension. Does local Rho activation also lead to long-range Rho and Rac partitioning? To test this, we used optogenetics to locally stimulate Rho (opto-LARG, Methods) while simultaneously measuring Rac activation using the biosensor Pak-PBD. **b**, Time-lapse confocal images of an unpolarized opto-Rac HL-60 cell expressing the Rac biosensor (Pak-PBD) before and during opto-Rho stimulation. Local Rho activation induces blebs at the opposite end of the cell, and these blebs are enriched in active Rac. **c**, Polar histogram of the spatial distribution of bleb appearance in relation to opto-Rho (cell back) showing that the majority of blebs are positioned at the opposite side of the cell back (opto-Rho). (*N* = 5, *n* = 52; here (and in **d**,**e**), *n* represents individual blebs). **d**, Histogram of bleb distribution in time during opto-Rho stimulation. (*N* = 3, *n* = 52). **e**, Average time trace of Rac activation (as measured by Pak-PBD) at the plasma membrane at the protrusive blebs. Time 0 = blebbing. For times before blebbing, Rac activation is measured at the same membrane spot where the bleb will appear (Methods) (mean ± 95% CI; *n* = 36, *N* = 3). **f**, In unpolarized cells at steady state, actin and MCA proteins are uniformly distributed. Upon local contraction (for example induced by opto-Rho), Actin flows toward the site of contraction. We ask whether MCA also flow with the cytoskeleton toward the site of contraction to induce an MCA asymmetry across the cell. **g**, Time-lapse confocal images of unpolarized cells expressing the MCA protein Ezrin–mCherry and stained with the membrane marker CellMask before and during opto-Rho stimulation. **h**, Average time trace of relative Ezrin intensity at the back and front of the cell in response to opto-Rho activation (at the back). (*N* = 3, *n* = 43, mean ± 95% CI). **i**, We hypothesized that Rho’s long-range stimulation of Rac might depend on Rho-based contractility, which in turn triggers blebbing. **j**, We locally activated Rho using optogenetics in the absence or presence of an inhibitor of myosin activation and blebbing (10 μM blebbistatin). **k**, Average time trace of Rac activation (as measured by Pak-PBD) at the plasma membrane measured at the cell front of control or cells treated with 10 μM Blebbistatin. These data indicate that myosin-based blebbing is important for Rho-mediated activation of Rac (mea*n* ± 95% CI; *n* = 19 (control) and 22 (blebbistatin), *N* = 3). **l**, Local Rho activation leads to long-range activation of Rac at the opposite side of the cell. Scale bars, 10 μm. Unless indicated otherwise, for all panels, *n*, individual cell replicate, *N*, biological replicates.

**Extended Data Fig. 5:**
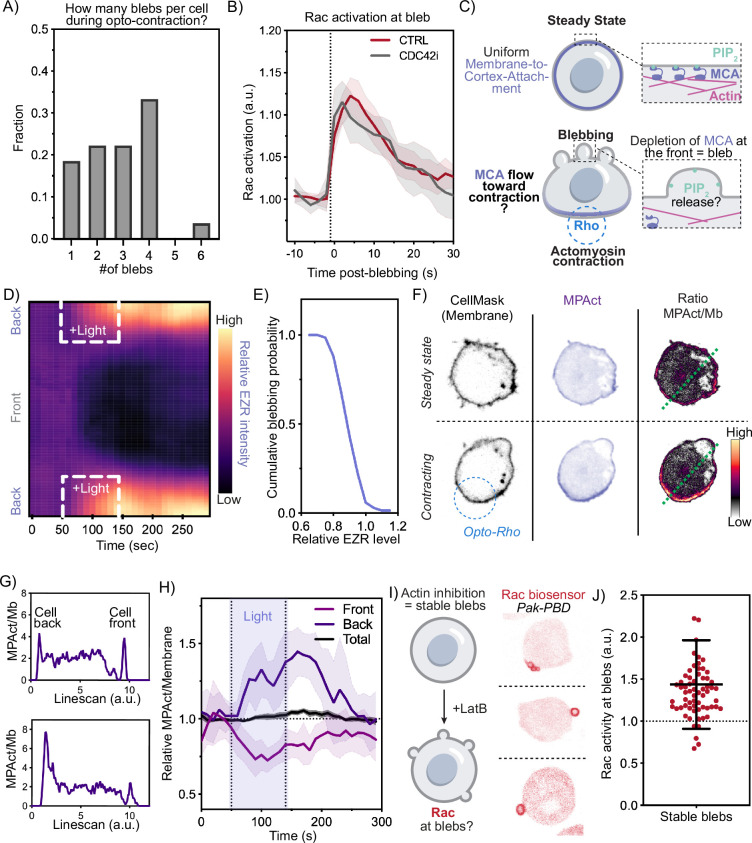
Contraction-induced membrane-to-cortex-attachment asymmetry leads to bleb-dependent Rac activation at the opposite side of the cell. (**A**) Histogram of the number of blebs per cell in response to opto-Rho stimulation (N = 3, n = 55). (**B**) Average time trace of Rac activation measured by Pak-PBD at the plasma membrane of control cells or cells treated with 10 micromolar of the CDC42 inhibitor ML141 following opto-Rho (LARG) activation showing that our Pak-PBD probe is responding to Rac and not CDC42 activity in blebs. (mean ± 95%CI; n = 20, N = 2, data for control is same as Fig. 4e). (**C**) In unpolarized cells at steady state, actin and MCA proteins are uniformly distributed. Upon local contraction (for example induced by opto-Rho), Actin flows toward the site of contraction. We ask whether MCA flows with the cytoskeleton toward the site of contraction to generate an MCA asymmetry across the cell (**D**) Kymograph of Ezrin intensity along the normalized cell circumference (y axis) showing that over time (x axis), Ezrin is enriched at the back in response to opto-Rho activation. (**E**) Cumulative blebbing probability as a function of relative Ezrin level. (N = 3, n = 40). (**F**) Time-lapse confocal images of unpolarized cells expressing the MPact, a membrane-actin proximal sensor^47^ which we use as a proxy for MCA level, and stained with the membrane marker CellMask before and during opto-Rho stimulation. The ratio between MPact and membrane channel is used to measure the approximate MCA level intensity. (**G**) Line-scan of MPAct/Membrane ratio of cell displayed in (**F**), see green dotted line. (**H**) Average time trace of relative MPAct/Membrane ratio (normalized to before light) at the front and back of the cell. (N = 2, n = 10, means ± 95%CI). (**I**) To determine whether blebs suffice for Rac activation in a context independent of Rho-mediated stimulation of actomyosin contractility, we used the actin inhibitor Latrunculin B to generate stable blebs. Right: three representative confocal images of cells expressing the Rac biosensor Pak-PBD treated with 10 μM of Latrunculin B demonstrate Rac enrichment in stable blebs. (J) Quantitation of Rac activation in Latrunculin B-induced stable blebs (see Methods) (mean ± 95%CI; n = 64 (here n= individual blebs), N = 3). Unless indicated otherwise, for all panels, n = individual cell replicate, N = biological replicates.

To investigate the ability of Rho to activate Rac, we used an optogenetic approach to locally activate the back polarity programme Rho (via LARG; Methods) while simultaneously monitoring Rac activation using the biosensor Pak-PBD (Fig. 4a and Supplementary Video 6). Our results show that Rho activation leads to long-range Rac activation at the opposite end of the cell, coinciding with another morphological change—blebbing (Fig. 4b and Extended Data Fig. 5a). Because the Pak-PBD probe can bind both active Rac and activated Cdc42, we made use of the well characterized Cdc42 inhibitor ML141 and show that it has no effect on opto-LARG-based generation of Pak-PBD recruitment at the opposite end of the cell (Extended Data Fig. 5b), suggesting this probe is primarily reading out Rac activation in our context.

Given previous studies linking blebbing to Rac activation^42,43^, we hypothesized that Rho’s long-range stimulation of Rac might depend on Rho-mediated contraction-induced blebbing (Fig. 4c–e and Extended Data Fig. 5c). Blebs form when the plasma membrane detaches from the underlying actin cortex. Rho induces blebbing at the opposite side of the cell through two complementary physical effects: (1) localized Rho leads to actomyosin flow toward the site of contraction^31^ which causes membrane-to-cortex-attachment (MCA) proteins to accumulate at the site of contraction while depleting them from the opposite end of the cell^44–46^ (Fig. 4f); and (2) a global increase in intracellular pressure (driven by cell deformation and increased cortical tension, via Laplace’s law) promoting membrane detachment even further. To test this, we imaged cells expressing a fluorescently tagged ezrin, a core MCA protein in neutrophils. Upon local Rho activation, ezrin accumulated at the site of contraction and was depleted at the opposite side of the cell (Fig. 4g,h, Extended Data Fig. 5d,e and Supplementary Video 7). To validate these findings, we used actin-membrane proximity biosensor MPAct^47^ to assess MCA distribution during opto-contraction. This tool confirmed that local contraction enriches MCA at the contraction site while depleting it from the opposite side of the cell (Extended Data Fig. 5f–h). These results suggest that the depletion of MCA opposite to the contraction site promotes the formation of bleb-based sites of Rac activation.

Finally, we further test the role of Rho-induced cellular contractions in the long-range positioning of Rac by using the actomyosin inhibitor blebbistatin to block cellular contraction following optogenetic Rho activation (Fig. 4i). Indeed, this inhibited blebbing and Rho could no longer activate Rac at a distance (Fig. 4j,k). Just as Rac requires its downstream cytoskeletal effects (actin polymerization and protrusion generation) to activate Rho at a distance (Extended Data Fig. 2), Rho relies on its downstream cytoskeletal impact (myosin-based contraction leading to blebbing) to activate Rac at a distance. To further explore whether blebs suffice to activate Rac independent of Rho-mediated stimulation of actomyosin contractility, we used the actin inhibitor latrunculin B to induce stable blebs^31^ (Extended Data Fig. 5i). These stable blebs showed robust Rac enrichment (Extended Data Fig. 5i,j). Our data demonstrate that local Rho activation stimulates Rac activation at the opposite end of the cell through actomyosin-based blebbing (Fig. 4l).

### Contraction-induced membrane-to-cortex-attachment asymmetry leads to PIP_2_ release and PI3K-dependent Rac activation at the opposite side of the cell

**Fig. 5:**
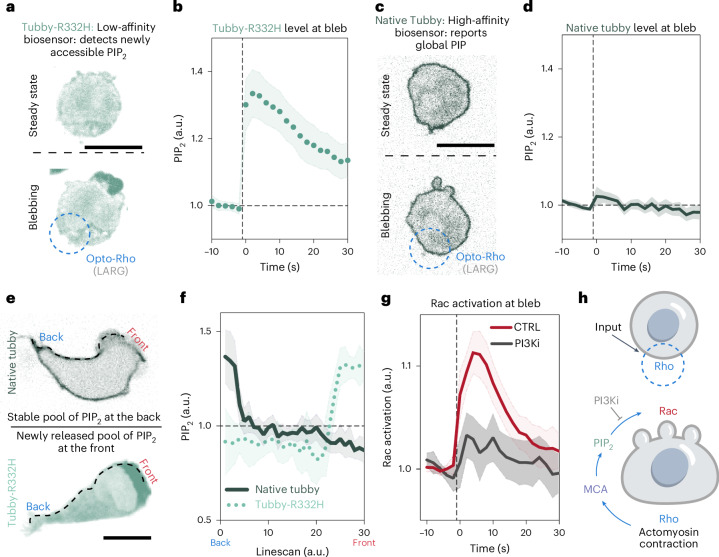
Contraction-induced blebbing leads to PIP_2_ release and PI3K-dependent Rac activation at the opposite side of the cell. Because MCA proteins such as ERMs bind to the membrane through interactions with PIP_2_ (ref. ^48^), we hypothesize that contraction-induced-MCA detachment from plasma membrane during blebbing leads to PIP_2_ release. **a**, Time-lapse confocal images of an unpolarized opto-Rac HL-60 cell expressing the low-affinity PIP_2_ biosensor (Tubby-R332H) before and during opto-Rho stimulation, demonstrating PIP_2_ enrichment in bleb-based protrusions. **b**, Average time trace of PIP_2_ accumulation at the protrusive blebs (opposite side of the cell from Rho activation) (mean ± 95% CI; *n* = 34, *N* = 3, n represents individual blebs). **c**,**d**, In contrast to the low-affinity PIP_2_ sensor, cells expressing a high-affinity PIP_2_ biosensor (Native-Tubby), which detects global PIP_2_, show only a mild increase at the cell front. **e**, Confocal images of migrating neutrophil-like HL-60 cells expressing either high (Native-Tubby, top) or low-affinity (Tubby-R332H, bottom) PIP_2_ biosensor showing that newly exposed PI(4,5)P_2_ localizes to transient flashes at the leading edge, whereas total PI(4,5)P_2_ is enriched at the rear (mean ± 95% CI; *n* = 16, *N* = 2). **f**, Average line-scan of PIP_2_ level across migrating HL-60s (mean ± 95% CI; *n* = 10, *N* = 2). **g**, Average time trace of Rac activation at the plasma membrane at the protrusive blebs in control and cells treated with 1 μM PI3Kδ/γ inhibitor, duvelisib demonstrates a requirement for PI3K for Rac activation in protrusive blebs (mean ± 95% CI; *n* = 30, *N* = 3). Control data are the same as in Fig. 4e. **h**, Local Rho activation leads to contraction-driven localized PIP_2_ enrichment at the cell front which acts as a substrate for the Rac activator PI3K. Scale bars, 10 μm. Unless indicated otherwise, for all panels, *n*, individual cell replicate; *N*, biological replicates.

We next explored the molecular mechanism linking blebbing to Rac activation, hypothesizing the lipid composition of the blebs to play a critical role. Because MCA proteins such as ERMs (Ezrin, Radixin and Moesin) bind to the membrane through interactions with PIP_2_ (ref. ^48^), detachment of MCA proteins from the membrane during blebbing could increase the local concentration of accessible PIP_2_. PI3K could act on this newly released PIP_2_ to produce PIP_3_, a known activator of Rac. To test this hypothesis, we used two versions of the PIP_2_ biosensor Tubby: Tubby-R332H, a low-affinity binder to PI(4,5)P2 that is thought to primarily recognize newly generated/exposed PI(4,5)P2 (ref. ^49^) and Native-Tubby, which recognizes total PI(4,5)P2 (ref. ^49^). Upon local opto-Rho activation, we observe a marked increase in newly generated/exposed PIP_2_ at blebs (Fig. 5a–d and Supplementary Video 8). When we compare the distribution of these PI(4,5)P2 probes in migrating HL-60 cells, the low-affinity Tubby domain (newly exposed PI(4,5)P2) localizes to flashes at the leading edge of the cell, whereas the high-affinity Tubby domain (total PI(4,5)P2) localizes to the rear of the cell (consistent with total PI(4,5)P2 localization in *Dictyostelium*^50^) (Fig. 5e,f and Supplementary Video 9), potentially indicating unique localization of the newly exposed versus total PI(4,5)P2.

**Extended Data Fig. 6:**
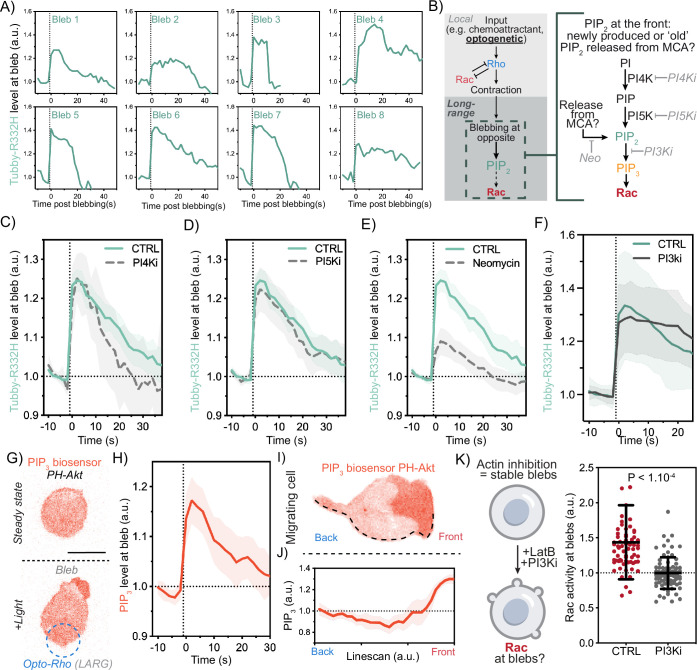
PI(4,5)P2 Synthesis inhibition does not prevent accumulation at cell front. (**A**) Individual examples of time trace of PIP_2_ accumulation, as measured by Tubby-R332H, at protrusive blebs (opposite side of the cell from Rho activation). (**B**) We speculate that the transient increases in PI(4,5)P2 observed at the cell front could arise from two possible mechanisms: release of pre-existing PI(4,5)P2 from PI(4,5)P2-binding proteins, such as MCA proteins, or *de novo* synthesis of PI(4,5)P2. To distinguish between these possibilities, we treated cells either with inhibitors of PI(4,5)P2 synthesis or with the PI(4,5)P2-sequestering drug neomycin. (**C**-**F**) Average time traces of PI(4,5)P2 levels (assayed with Tubby-R332H) at the plasma membrane at the protrusive blebs in control (n = 35, N = 3) and cells treated with either 200 nM of PI4K inhibitor (GSK-A1; n = 8, N = 2), 5 μM of PI5K inhibitor (PIP5K1C-IN-1; n = 27, N = 3), 10 mM of Neomycin (n = 50, N = 3), or 1 μM PI3Kδ/γ inhibitor Duvelisib (n = 30, N = 3) (mean ± 95%CI). (**G**) Time-lapse confocal images of an unpolarized opto-Rac HL-60 cell expressing the PIP_3_ biosensor (PH-Akt) at steady state and during opto-Rho stimulation showing that contraction-induced blebbing leads to elevated PIP_3_ at the cell front. (**H**) Average time trace of PIP_3_ level at the plasma membrane at the protrusive blebs, showing PIP3. Time 0 = blebbing. For times before blebbing, PIP_3_ activity is measured at the same membrane spot where the bleb will appear. (mean ± 95%CI; n = 20, N = 2). (**I**) Confocal image of a migrating neutrophil-like HL-60 cell expressing the PIP3 biosensor PH-Akt. (**J**) Average line-scan of PIP3 level across migrating HL-60s, showing enrichment of PIP_3_ at the cell front (mean ± 95%CI; n = 15, N = 2). (**K**) Rac activation in stable blebs in control cells and cells treated with 1 μM PI3Kδ/γ inhibitor show that PI3K inhibition prevents Rac activation in stable blebs. (N = 3, n = 77 (here n= individual blebs), data for control are the same as Fig. S5J, p values from Mann Whitney test). Unless indicated otherwise, for all panels, n = individual cell replicate, N = biological replicates.

We speculate that the transient increases in PI(4,5)P2 observed at the cell front could arise from two possible mechanisms: release of pre-existing PI(4,5)P2 from PI(4,5)P2-binding proteins, such as MCA proteins or de novo synthesis of PI(4,5)P2. To distinguish between these possibilities, we treated cells either with inhibitors of PI(4,5)P2 synthesis or with the PI(4,5)P2-sequestering drug neomycin. Notably, inhibition of PI(4,5)P2 synthesis did not reduce PI(4,5)P2 levels (as assayed by the low-affinity Tubby-R332H) at the cell front, whereas neomycin treatment caused a pronounced decrease in PI(4,5)P2 at the front (Extended Data Fig. 6b–f). These results support the release-based mechanism as the more likely origin of the transient PI(4,5)P2 increase.

**Extended Data Fig. 7:**
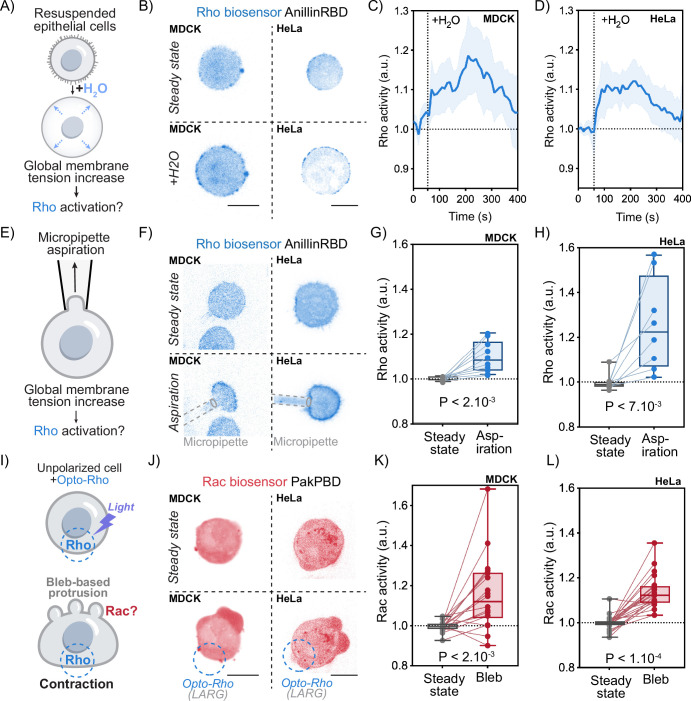
Core components of Rho and Rac mutual long-range activation are conserved in HeLa and MDCKs. (**A**) Are the core components of the long-range communication from Rac to Rho conserved in non-migratory cells? If membrane tension operates through mTORC2 to activate Rho in epithelial cells, then increasing membrane tension should suffice to activate Rho. Towards this end, we used hypotonic shock to elevate membrane tension in resuspended epithelial cells. (**B**) Confocal images of HeLa and MDCKs cells expressing the Rho biosensor AnillinRBD before and during osmotic shock. (**C**)(**D**) Average time trace of Rho activation at the plasma membrane in response for hypotonic shocks of MDCKs ( + 50% H_2_O) and HeLa cells ( + 65% H_2_O) (N = 2, n = 20, means ± 95%CI). (**E**) We used micropipette aspiration as another mean to induce membrane deformation. (**F**) Time-lapse confocal images of HeLa and MDCK cells expressing the Rho biosensor (AnillinRBD) before and after micropipette aspiration. (**G**)(**H**) Average Rho activation before (steady state) and during aspiration of MDCK and HeLa cells. (**I**) (**J**) Time-lapse confocal images of an unpolarized opto-Rho MDCKs and HeLa cells expressing the Rac biosensor (Pak-PBD) at steady state and during opto-Rho stimulation. Local Rho activation induces blebs at the opposite end of the cell, and these blebs are enriched in active Rac. (**K**)(**L**) Relative Rac activation level at the plasma membrane at steady state and at protrusive blebs. for **G**), **H**), **K**), **L**): Boxes and whiskers median and min to max; p values from Wilcoxon paired Student’s t test. Scale bars: 10μm. Unless indicated otherwise, for all panels, n = individual cell replicate, N = biological replicates.

Next, we examined whether PI3K could act on this newly released PIP_2_ to produce PIP_3_, a known activator of Rac. As expected, PIP_3_ increases are observed (as assayed by a PH-AKT biosensor) at sites of blebbing following opto-Rho activation (Extended Data Fig. 6h,i) and at the front of migrating cells (Extended Data Fig. 7j,k). We further test the functional role of this PIP_3_ in Rac activation within blebs by inhibiting PI3K using duvelisib (PI3Ki), a dual PI3Kδ/γ inhibitor^51^ (Fig. 5g and Extended Data Fig. 6g). Cells treated with duvelisib displayed impaired Rac enrichment downstream of both opto-Rho-mediated blebs (Fig. 5j) as well as latrunculin-induced stable blebs (Extended Data Fig. 6g). Together, these findings provide a molecular mechanism to link local Rho activation to Rac activation at the opposite end of the cell. Local Rho-induced contraction redistributes MCA proteins away from the opposite end of the cell, promoting blebbing. This depletion of MCA proteins induced blebbing releases PIP_2_, which serves as a substrate for PI3K to generate the Rac activator PIP_3_(Fig. 5h).

### Core components of the long-range mutual Rho and Rac activation are conserved in non-migratory cells

**Fig. 6:**
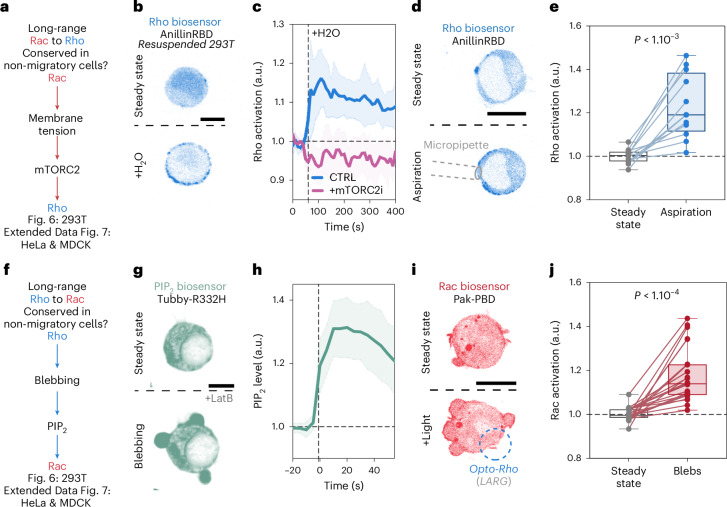
Core components of Rho and Rac mutual long-range activation are conserved in non-migratory cells. **a**, Are the core components of the long-range communication from Rac to Rho conserved in non-migratory cells? If membrane tension operates through mTORC2 to activate Rho in epithelial cells, then increasing membrane tension should suffice to activate Rho. Towards this end, we used hypotonic shock and micropipette aspiration to elevate membrane tension in resuspended epithelial cells. **b**, Confocal images of 293Ts cells expressing the Rho biosensor AnillinRBD before and during osmotic shock. **c**, Average time trace of Rho activation at the plasma membrane in response for hypotonic shocks of control cells (in blue) or cells treated with the 10 μM mTOR inhibitor KU-0063794 (in pink) (*N* = 2, *n* = 19, mean ± 95% CI. **d**, Time-lapse confocal images of 293T cells expressing the Rho biosensor (AnillinRBD) before and after micropipette aspiration. **e**, Average Rho activation before (steady state) and during aspiration showing Rho activation in response to membrane deformation (Boxes and whiskers: median and min to max; *N* = 2, *n* = 11; *P* values from Wilcoxon paired Student’s *t*-test). **f**, Are the core components of the long-range communication from Rho to Rac conserved in non-migratory cells? **g**, Time-lapse confocal images of 293T cells expressing the PIP_2_ biosensor (Tubby-R332H) before and during latrunculin B treatment demonstrates that PIP_2_ enrichment in bleb-based protrusions is conserved in non-migratory cells. **h**, Average time trace of PIP_2_ accumulation at the blebs in 293T cells treated with 2 μM actin inhibitor latrunculin B (mean ± 95% CI; *n* = 30, *N* = 2). **i**, Time-lapse confocal images of an unpolarized opto-Rho 293T cell expressing the Rac biosensor (Pak-PBD) at steady state and during opto-Rho stimulation. Local Rho activation induces blebs at the opposite end of the cell, and these blebs are enriched in Rac. **j**, Relative Rac level at the plasma membrane at steady state and at protrusive blebs (Boxes and whiskers show median and min to max; *N* = 2, *n* = 23; *P* values from Wilcoxon paired Student’s *t*-test. Scale bars, 10 μm. Unless indicated otherwise, for all panels, *n*, individual cell replicate; *N*, biological replicates.

Our experiments demonstrate that Rho and Rac mutually activate one another at a distance using two distinct mechanisms: (1) membrane tension, which links front generation with back activation; and (2) cortical remodelling, which connects back activation with front generation. Given the broad conservation of the components of the pathways we have identified for this long-range coupling (membrane tension propagation, mTORC2 activation, cortical flows and phospholipid changes), we next investigated the generality of this circuit beyond cell migration. First, we probed whether tension/mTORC2-mediated increases in Rho activation via mTORC2 are conserved in non-migratory cells (Fig. 6a). To investigate this possibility, we used hypotonic shock in resuspended epithelial cells (Hek-293Ts, MDCKs and HeLa) to stimulate an increase in membrane tension. Consistent with our previous findings, we observed that hypotonic shock induced a rapid and global increase in Rho activation across the cell (Fig. 6b,c and Extended Data Fig. 7a–d). Cells treated with mTOR inhibitor KU-0063794 failed to activate Rho in response to hypotonic shock. As an alternate approach to generate membrane deformation, we used micropipette-based aspiration to elicit global Rho activation (Fig. 6d–e and Extended Data Fig. 7e–h). These data show a conservation of the pathway linking membrane tension to mTORC2 to Rho activation in non-migratory cells.

Next, we probed whether the MCA-mediated release of PIP_2_ and subsequent Rac activation at blebs is also conserved in non-migratory cells (Fig. 6f). Towards this end, we used the actin inhibitor latrunculin B to induce stable blebs^31^ in resuspended epithelial cells expressing the low-affinity PIP_2_ biosensor Tubby-R332H and observed a marked increase in PIP_2_ in blebs (Fig. 6g,h). To test whether the activation of Rac in blebs is also conserved in non-migrating cells, we next imaged epithelial cells expressing the Rac biosensor Pak-PBD before and during opto-Rho activation and find Rac enrichment in blebs, consistent with our observations in HL-60 cells (Fig. 6i,j and Extended Data Fig. 7i–l). Together, our results show that the core components of the long-range mutual activation between Rac and Rho are conserved in non-migratory cells and are likely to account for the robustness of polarity in a number of other cellular contexts, such as collective cell migration^52^, epithelial polarity^53^ and asymmetric cell division^54^, all of which involve long-range Rac/Rho patterning.

### Mechanochemical model of Rho and Rac partitioning in cells

**Fig. 7:**
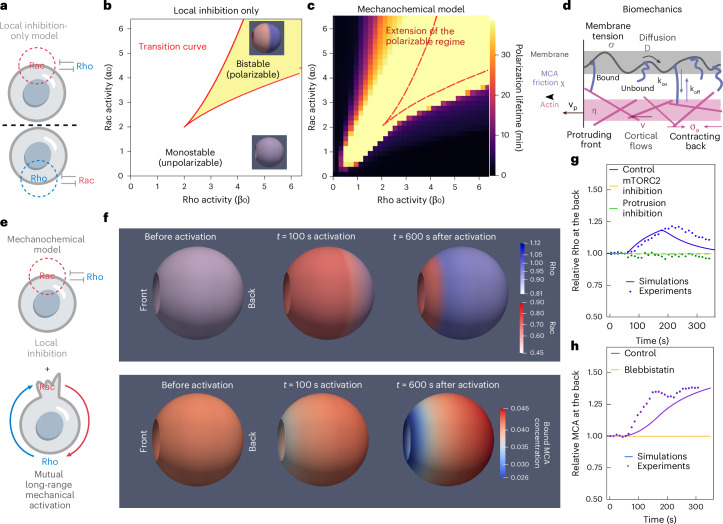
Modelling Rac and Rho polarization mechanisms through local inhibition and long-range mechanical feedback. **a**, Schematic of a simple local mutual-inhibition model between Rac and Rho. **b**, Phase diagram from stability analysis of the local mutual-inhibition model. For given basal Rac and Rho activities (α0 and β0, respectively), the system is either monostable (unpolarized) or bi-stable (polarizable). Inset shows example simulations showing unpolarized and polarized states. **c**, Heatmap of the relaxation time from a polarized state back to steady state as a function of basal Rac and Rho activities (α0 and β0) in the model combining local inhibition with long-range mechanical feedback. The dashed curve indicates the bi-stable region predicted by local inhibition alone. **d**, One-dimensional schematic of the coupled mechanochemical model generating long-range mechanical feedback together with local mutual inhibition. **e**, Simulation protocol: Rac is locally activated at one end of the cell (‘front’), and Rho activity is quantified at the opposite end (‘back’). **f**, Two-dimensional simulation of the integrated mechanochemical model. Top: local Rac activation at the front induces progressive Rho recruitment at the back (left, before activation; middle, 200 s after activation; right, 600 s after activation). Bottom: surface density of membrane–cortex-attachment linkers (for example, ezrin) before activation (left), 100 s (middle) and 600 s (right). **g**, Rho activity at the back following Rac activation at the front: simulations (solid lines) and experiments (dots). Control cells, mTORC2-defective cells (Rictor KO) and protrusion-inhibited cells (CK666). **h**, Ezrin levels at the back: simulations (solid lines) and experiments (dots) show that local Rho activity drives membrane–cortex-attachment asymmetry.

To understand the key requirements for the long-range Rho and Rac mutual activation, we developed a mechanochemical model of Rho and Rac partitioning. We first formulated a biochemical model based on two coupled differential equations capturing local mutual inhibition between Rac and Rho^55^ (Fig. 7a,b and Supplementary Note, section 2). The model features two control parameters describing antagonism-modulated activation of the two GTPases, denoted α0 and β0. At low overall activation, or when activation is highly asymmetric, the system is monostable and converges to a state dominated by either Rho or Rac. At sufficiently high activation of both GTPases, the system enters a bi-stable regime, in which high-Rac and high-Rho states can both exist, and, in principle support spatial segregation (Fig. 7b). However, in this purely biochemical framework, polarization cannot be maintained in the presence of diffusion alone (Supplementary Note, section 2.4). This highlights the limitations of polarity regulation based solely on local inhibition, consistent with our experimental results (Extended Data Figs. 2a–f and 3a–d).

**Extended Data Fig. 8:**
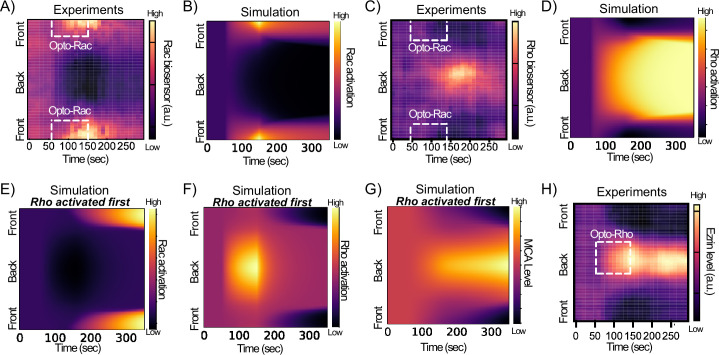
Kymographs comparing experimental and simulated data. Kymographs from experiments and simulations. (**A**) Kymograph of Rac biosensor level across time in Opto-Rac stimulated cells (same data as Fig. 1d). (**B**) Simulated Rac activation in response to Rac input at the front. (**C**) Kymograph of Rho biosensor level across time in Opto-Rac stimulated cells (Same data as Fig. 1g). (**D**) Simulated Rho activation in response to Rac activation at the front. (**E**)(**F**)(**G**) Kymographs of simulated Rho activation, Rac activation, and MCA levels in response to local Rho activation. (**H**) Kymograph of Ezrin level across time in Opto-Rho stimulated cells (Same data as Fig. 4h).

**Extended Data Fig. 9:**
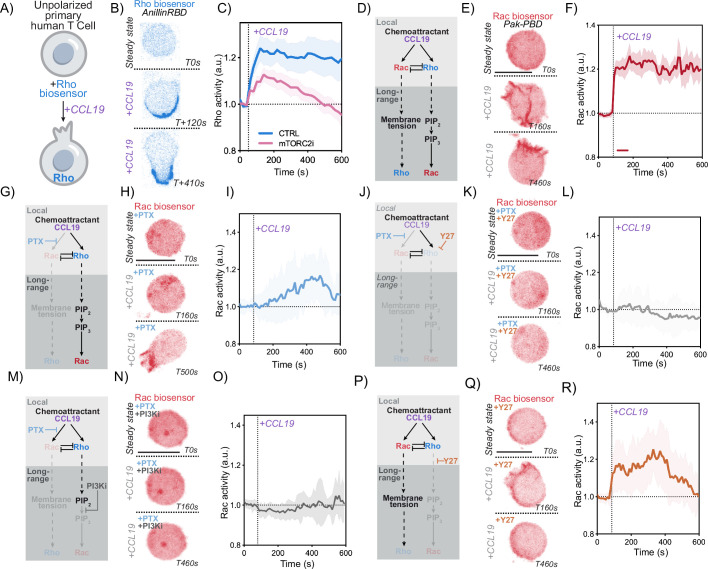
Long-range mutual activation establishes robust Rho and Rac polarity in primary T cells. (**A**) (**B**) Time-lapse confocal images of an unpolarized human primary T cell before and during CCL19 stimulation. Rho activity was monitored via the Rho biosensor AnillinRBD. (**C**) Average time trace of Rho activity at the cell back following addition of 25 nM of CCL19 comparing control cells and cells treated with 10 μM of the mTORC2 inhibitor KU. (mean ± 95%CI; CTRL n = 30, KU n = 20, N = 3). (**D**) Primary human T cells expressing the Rac biosensor (Pak-PBD) to assay cell polarization following acute stimulation with the chemoattractant CCL19. CCL19 is known to activate both Rho and Rac. (**E**) Time-lapse confocal images of an unpolarized human primary T cell before and during CCL19 stimulation. Rac activation was monitored via the Rac biosensor Pak-PBD. (**F**) Average time trace of Rac activation at the cell front following addition of CCL19. (mean ± 95%CI; n = 30, N = 3) (**G**) (**H**) Cells are treated with GαI inhibitor to prevent direct Rac activation by CCL19. (**I**) Average time trace of Rac activation at the cell front of cells treated with 1 ug ml of GαI inhibitor following addition of CCL19. (mean ± 95%CI; n = 20, N = 3). (**J**)(**K**) Cells are treated with a combination of Y27 and PTX to prevent direct Rho and Rac activation by CCL19. (**L**) Average time trace of Rac activation at the cell front of cells treated with 1 ug ml of GαI inhibitor PTX together with 20 μM of Y27 following addition of CCL19 (mean ± 95%CI; n = 17, N = 3). (**M**)(**N**) Cells are treated with a combination of PTX and the PI3K inhibitor Duvelisib to disrupt the long-range Rho-mediated Rac activation described in this study. (**O**) Average time trace of Rac activation at the cell front of cells treated with 1 ug/ml of GαI inhibitor PTX together with 1µM PI3K inhibitor Duvelisib. (mean ± 95%CI; n = 13, N = 2). (**P**)(**Q**) Cells are treated with ROCK inhibitor Y27 to prevent Rho activation. (**R**) Average time trace of Rac activation at the cell front of cells treated with 20 μM of Y27 following addition of CCL19 (mean ± 95%CI; n = 19, N = 3). Data for (**C**), (**F**), (**I**) and (**O**) are similar as in Fig. 8 but separated into different panels for extra clarity. For all panels, n = individual cell replicate, N = biological replicates.

Next, we integrated this mutual inhibition module with a mechanical description of membrane–cortex coupling, building on previous works^31^ (Supplementary Note, section 4 and Fig. 7d). The cortex is modelled as a viscous, contractile layer attached to the membrane through MCA proteins with surface density *ρ*. The mechanically relevant membrane tension (also referred to as frame tension^56,57^) is stored in entropic and cortical-flow-driven membrane ruffles, which are minimally modelled as linearly elastic elements. Rac promotes local protrusion at the front, represented by a protrusive velocity *v*_*p*_, while Rho drives local contraction in the cortex, represented by an active stress *σ*_a_. Based on experimental findings, we introduced two additional couplings: (1) Rac activation is negatively modulated by MCA protein density; and (2) Rho is enhanced by membrane tension, with both couplings implemented using switch-like response functions. This integrated mechanochemical model reproduces our previous observation that local opto-Rac activation leads to Rho activation at the opposite end of the cell (Fig. 7e–g, Extended Data Fig. 8a–d and Supplementary Video 10). In agreement with experiments, blocking Rac-driven membrane tension generation (for example, via drugs inhibiting protrusion) or disrupting tension-dependent Rho activation (for example, in mTORC2 mutants) prevents distal Rho activation. Thus, the model explains how the front positions the back by combining local mutual inhibition with long-range, tension-mediated activation of Rho. We then simulated the reverse scenario by locally activating Rho and measuring Rac at the opposite end. The model captures how Rho generates MCA asymmetry (Fig. 7f,h and Extended Data Fig. 8e–h) and induces Rac activation at the distal pole (Extended Data Fig. 9e).

**Fig. 8:**
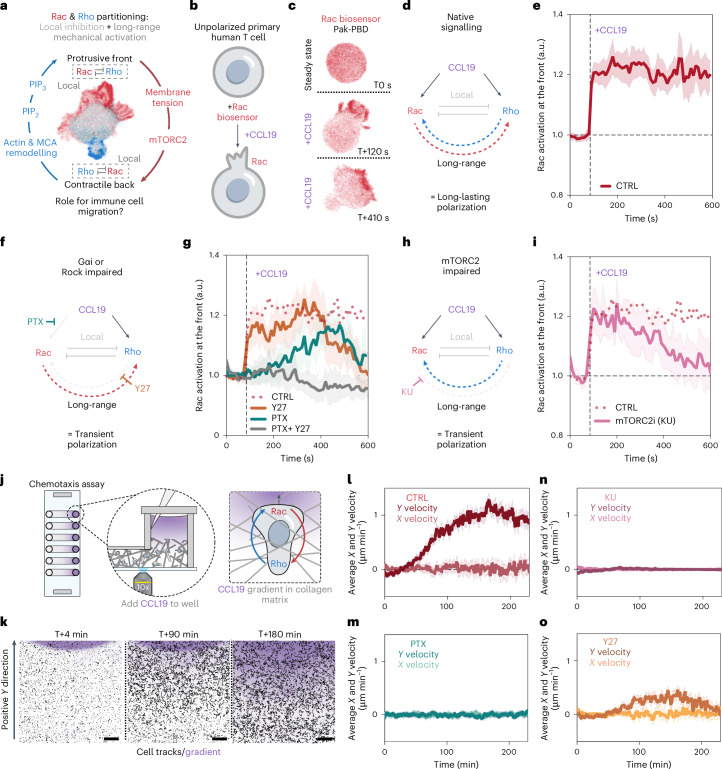
Long-range mutual activation establishes robust Rho and Rac polarity during primary T cell migration. **a**, Confocal image of a migrating human primary T cell expressing the polarity biosensors for Rac (PAK-PBD) and Rho (AnillinRBD). Activating either Rho or Rac leads to a robust establishment of polarity and that Rho and Rac both mutually reinforce each other at a distance. **b**, Primary human T cells expressing the Rac biosensor (Pak-PBD) to assay cell polarization following acute stimulation with the chemoattractant CCL19. **c**, Time-lapse confocal images of an unpolarized human primary T cell before and during CCL19 stimulation. Rac activation was monitored via the Rac biosensor Pak-PBD. **d**, Chemoattractants such as CCL19 are known to activate both Rho and Rac. **e**, Average time trace of Rac activation (measured by Pak-PBD level) at the cell front following addition of 25 nM CCL19 showing that CCL19 induces long-lasting polarization (mean ± 95% CI; *n* = 30, *N* = 3). **f**, To test whether long-lasting Rac partitioning depends exclusively on local Gαi-mediated activation or also requires long-range Rho/myosin signalling, cells were treated with 1 μg ml^−1^ Gαi inhibitor PTX, cells treated with 20 μM Y27 and a combination of PTX and 20 μM Y27. **g**, Average time trace of Rac activation (measured by Pak-PBD level) at the cell front following addition of 25 nM CCL19 in cells treated with either PTX, Y27 or a combination of both. PTX and Y27 treated cells polarized only transiently, while cells treated with both failed to polarize altogether when compared with control cells (mean ± 95% CI; *n* = 20 for PTX, *n* = 19 for Y27, *n* = 15 for PTX + Y27, *N* = 3). **h**, We prevent the long-range Rac to Rho communication using the mTORC2 inhibitor KU-0063794. **i**, Average time trace of Rac activation (measured by Pak-PBD level) at the cell front following addition of 25 nM CCL19 in cells treated with 10 μM KU-0063794 showing that cells with impaired mTORC2 signalling only polarize transiently in response to CCL19 (mean ± 95% CI; *n* = 20, *N* = 2). **j**, Ex vivo assay for human primary T cell chemotaxis. Cells are premixed with bovine dermal collagen and placed into linear channels. After collagen sets, the medium is added to one side of the channel (TCM) and medium with human CCL19 (100 ng total) and 10 μg ml^−1^ Dextran10k-AF647 (Dex647) is added to the other. Imaging takes place right on the edge of the well containing CCL19 so that T cell responses can be recorded as the CCL19 (as read out by Dex647) diffuses into the channel. **k**, Representative control experiment is shown at different time points with Dex647 fluorescence (top row) and corresponding tracks of T cells (bottom row); tracks display 3 min preceding time point listed. **l**–**o**, Average *Y* only component of velocity of all tracks at each time point recorded. Positive is considered towards the well containing CCL19. Control Volunteer *N* = 3, PTX Volunteer *N* = 3, Y27 Volunteer *N* = 2, KU Volunteer *N* = 2. For all panels, *n*, individual cell replicate, *N*, biological replicates.

Finally, we compared the persistence of polarity generated by the integrated mechanochemical model with that produced by two alternative models: one based solely on local Rac–Rho mutual inhibition, and another using wave-pinning dynamics^16–19^ driven by diffusion and mass conservation (Supplementary Note, section 3). Our results indicate that local mutual inhibition alone is insufficient to robustly partition Rho and Rac across the cell (Supplementary Note, sections 2 and 3). Although wave-pinning models can produce stable polarization, they exhibit hypersensitivity and a binary response to external stimuli or fluctuations in Rho and Rac—features that are inconsistent with the broader spectrum of cellular behaviours observed experimentally. In contrast, the integrated model, which combines local mutual inhibition with long-range mutual activation via mechanochemical coupling, successfully recapitulates experimentally observed behaviours. Specifically, it predicts three distinct regimes of cellular behaviour across a wide range of Rac and Rho basal production levels: (1) a transiently polarizable state, characteristic of HL-60 cells; (2) a responsive and polarizable state, in which sufficiently strong stimulation can induce persistent polarization through mechanochemical feedback, consistent with the response of primary human T cells (Fig. 8); and (3) a spontaneously polarized state, where the cell maintains a persistent polarized configuration without external cues (Supplementary Note, sections 4–8).

### Long-range mutual activation establishes robust Rho and Rac polarity during primary T cell migration

Having shown that activating either Rho or Rac leads to a robust establishment of polarity and that Rho and Rac both mutually reinforce each other at a distance (Fig. 8a), we next investigated the consequences of this long-range crosstalk for immune cell polarization and migration. To this end, we used primary human T cells expressing the Rac biosensor (Pak-PBD) or the Rho biosensor (AnillinRBD) to assay cell polarization following acute stimulation with the chemoattractant CCL19 (Fig. 8b). We found that CCL19 induces rapid cell polarization with sustained Rac activation at the front and Rho activation at the cell back (Fig. 8c–e, Extended Data Fig. 9a–f and Supplementary Video 11).

CCL19 is known to activate both Rac and Rho^58,59^. We first tested whether our observed long-lasting Rac partitioning depends solely on the known local activation of Rac via Gαi signalling or if it also depends on the long-range activation via the Rho–myosin pathway defined in this work. To focus on the role of Rho–myosin on Rac regulation, we first treated cells with pertussis toxin (PTX) to inactivate Gαi signalling (Fig. 8f). These cells maintained their ability to stimulate Rac activation and polarization, albeit with a marked delay and more transiently than control cells (Fig. 8g and Extended Data Fig. 9f,g). The ability of PTX treated cells to polarize in response to chemokines has been previously reported but the mechanism by which this is achieved is unknown^6^. To test whether this residual Rac regulation occurred through long-range Rho/myosin activation, we treated cells with both PTX and the actomyosin inhibitor Y27632 (Fig. 8g and Extended Data Fig. 9j–l). This combination completely abolished even transient Rac polarization, consistent with a long-range back-to-front signalling link that acts in conjunction with Gαi-based Rac activation.

We previously showed that long-range Rac activation by Rho is dependent on PI3K which locally converts PIP_2_ into the Rac activator PIP_3_ at the cell front. We further investigated whether chemoattractant induced back-to-front signalling is also dependent on PI3K activation and found Rac polarization to be blocked when using both PTX (which blocks the Gαi-based Rac activation) and the PI3K inhibitor duvelisib (which blocks the long-range Rho-based Rac activation; Extended Data Fig. 9m–o). Finally, we show that preventing the long-range Rho to Rac communication with Y27632 or inhibiting the long-range Rac to Rho communication using the mTORC2 inhibitor KU-0063794 both elicit a more transient polarization than is seen for control cells (Fig. 8h,i and Extended Data Fig. 9p–r).

These results demonstrate that chemoattractant signalling communicates with the front programme in two different ways, one direct route via Gαi and one indirect route via the back-mediated mechanism that we define in this work. Cells deficient in either the direct or indirect (long-range) route can only polarize transiently, whereas wild-type primary cells combine both direct and indirect routes of Rac activation for robust and sustained polarization in response to chemoattractant.

**Extended Data Fig. 10:**
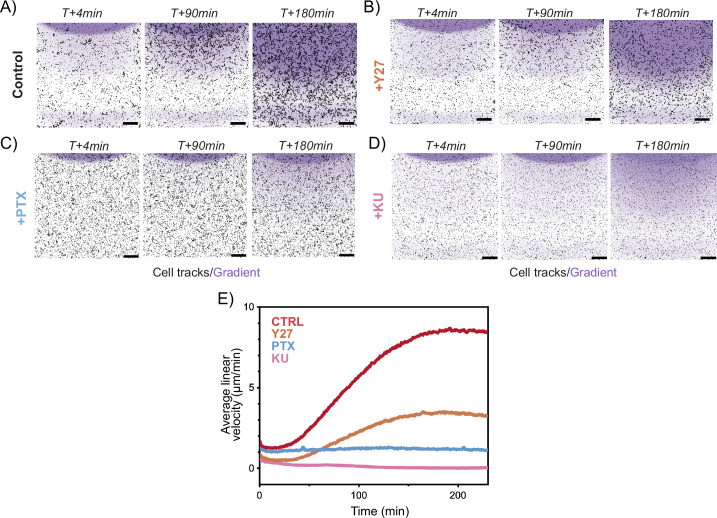
Long range mutual activation is necessary for chemotaxis in human primary T cells. Ex-vivo assay for human primary T cell chemotaxis. Cells are premixed with Bovine Dermal Collagen and placed into linear channels. After collagen sets, media is added to one side of the channel (TCM) and media with human CCL19 (100 ng total) and 10 μg ml^−1^ of Dextran10k-AF647 (Dex647) is added to the other. Imaging takes place right on the edge of the well containing CCL19 so that T cell responses can be recorded as the CCL19 (as read out by Dex647) diffuses into the channel. (**A**) Representative control, (**B**) Y27, (**C**) PTX, (**D**) KU experiment at 4 min, 90 min, 180 min with Dex647 fluorescence and corresponding tracks of T cells; tracks display 3 min preceding time point listed. (**E**) Average linear velocity of all tracks at each time point recorded. Control Volunteer N = 3, PTX Volunteer N = 3, Y27 Volunteer N = 2, KU Volunteer N = 2 (mean ± 95%CI).

Finally, we sought to test the role of this robust polarization established by the long-range Rac–Rho communication during chemoattractant induced migration. To stimulate chemotaxis, we embedded T cells in a three-dimensional (3D) collagen matrix in a thin channel with CCL19 placed on one side (Fig. 8j). As CCL19 freely diffuses into the channel, a chemotactic gradient is established, and control cells rapidly polarize and chemotax up the CCL19 gradient (Fig. 8k,l and Supplementary Video 12). In contrast, cells treated with PTX, KU-0063794 or Y27 only briefly polarized and failed to robustly migrate toward the CCL19 gradient (Fig. 8m–o and Extended Data Fig. 10).

Our results show that long-range mutual activation of front and back is an essential feature of human lymphocyte polarization and motility. Furthermore, we show that activating either the front or back polarity programmes without the other only leads to transient polarization. Dual activation is required to support sustained polarization and efficient chemotactic migration.

## Discussion

Our work demonstrates that the well-appreciated mutual short-range inhibition between Rac and Rho is accompanied by an additional mutual long-range facilitation of the front and back polarity programmes in migrating cells (Fig. 8a). The front reinforces the back via membrane tension-mediated mTORC2 activation (Figs. 1–3 and Extended Data Figs. 1–4). The back reinforces the front via cortical remodelling and localized PIP_2_ release (Figs. 4 and 5 and Extended Data Figs. 5 and 6). Both modes of long-range facilitation are required for sustained and efficient front organization and chemotaxis in primary human lymphocytes **(**Fig. 8 and Extended Data Figs. 9 and 10). Understanding the partitioning of Rho and Rac in migrating cells has long been a challenge due to the difficulty in establishing the range and directionality of interactions of Rac and Rho. Our use of optogenetics in unpolarized cells enables us to dissect the long-range positive interactions between the front and back programmes, and our combined mechanical and biochemical models complement our experimental work by revealing the importance of these links to cell polarization. Our combined multidisciplinary approach is likely to be similarly powerful for other complex interconnected spatiotemporal signalling networks such as axon specification^60^, asymmetric cell division^54^, epithelial polarity^53^, planar cell polarity^61^, branching morphogenesis^62^, lumen formation^63^ and collective cell migration^52^.

Robust cell polarization requires long-range integration of signals across the cell. A number of currencies have been proposed for this long-range signal relay: diffusion of signals across the cell^14,17,55,64,65^ or force-mediated communication via propagation of forces in the membrane or cortex^26–28,30,66^ or advection of cellular components via force asymmetries across the cell^67–69^. Membrane tension rapidly propagates across cells and is used for long-range integration of cellular processes from cell spreading^70,71^ to phagocytosis^72^ to the competition of fronts that enables a dominant protrusion in migrating cells^27,28^. Tension-based communication is particularly powerful for global signal transmission across the cell^41^. Our work shows that membrane tension also enables the front to facilitate contraction at the cell rear through mTORC2 activation. Previous studies^35,73–78^, including our own^28,29^, have broadly linked TORC2 and mTORC2 to cell polarity, but how mTORC2 does so is not well understood. Even its links to myosin regulation have been a point of contention, with some studies showing a positive role^29^ and others proposing a negative role^35^. Our work provides direct evidence for and a molecular mechanism for this link (Fig. 3). In addition, our work shows that mTORC2 localizes to the leading edge of migrating cells, to the site of protrusions in opto-PI3K-simulated cells, and to the plasma membrane in osmotically swollen cells (Fig. 3e–h and Extended Data Fig. 4m,n). In contrast, mTORC2 enrichment decreases at the site of contraction in opto-LARG simulated cells and at the back of migrating cells (Fig. 3g,h and Extended Data Fig. 4k,l). These results are consistent with a mechanosensory complex that responds to protrusion-induced increases in in-plane membrane tension. If increased mTORC2 localization reflects increased activity, then linking elevated mTORC2 signalling at the leading edge to Rho activation at the trailing edge would likely require soluble effectors. Notably, precedent for such front-back communication exists in *Dictyostelium*, where local PI3K activity at the cell front was shown to promote phosphorylation of PAK, a regulator of the cell rear^38,39^.

The evolutionarily conserved mTORC2 is also the mechanosensitive pathway that enables competition between fronts^28,29^, regulation of plasma membrane homeostasis in budding yeast^79,80^, and chemotaxis in cells from neutrophils^35^ to *Dictyostelium*^75^. We show that this link is also conserved in non-migratory epithelial cells. Given the evolutionarily conserved role of mTORC2, it would be interesting to probe in future work whether the mTORC2-mediated activation of Rho in response to elevated membrane tension is important in other contexts such as cell division.

Force-mediated flow of components is another powerful mechanism of long-range cell communication^67,81,82^. Here, we show that redistribution of MCA components driven by Rho-mediated actin-based contractions at the back and front-to-back flows enables the cell rear to establish a Rac-based protrusive front. These flow-mediated modes of communication provide an efficient route to polarize signalling by selectively redistributing molecular ‘constraints’—in this case, locally depleting MCA and thereby facilitating Rac activation distal to the contraction site. More generally, transport of molecular signals from front to back via retrograde flows and cortical contractions has been proposed to enable robust polarization^67,81,82^. Our work provides one possible molecular mechanism for this long-range integration. Consistent with previous studies, we find that localized PI3K signalling is more closely coupled to newly exposed PI(4,5)P_2_ at the cell front than to the distribution or dynamics of the total PI(4,5)P_2_ pool (Fig. 5**)**. Using a low-affinity Tubby probe that preferentially reports newly exposed PI(4,5)P_2_, we observe transient flashes at the leading edge, whereas high-affinity Tubby probe reporting total PI(4,5)P_2_ is enriched at the rear; patterns consistent with observations in other motile cells *Dictyostelium* and other migrating cells^50,83^. These observations suggest that force and flow-mediated redistribution of membrane components may selectively expose PI(4,5)P_2_ at the front, thereby providing a locally accessible substrate for PI3K and reinforcing Rac-dependent protrusive polarity.

It is notable that the long-range back-to-front and front-to-back communication involve different currencies of membrane tension and cortical flow. These different currencies have different spatial patterns of propagation (relatively global for membrane tension and polarized for cortical flows) and also differ in their molecular requirements, potentially enabling these modes of communication to be independently modulated without interfering with one another.

Rho GTPases are central regulators of the cytoskeleton, and their partitioning underlies many fundamental cellular functions such as cell shape, adhesions and division^1^. In the context of cell locomotion, we find that long-range activation is a key complement to local inhibition to ensure the proper partitioning of Rac and Rho at opposite poles of the cell. Our findings and model highlight the limitations of a system that relies only on local interactions for spatial segregation. We speculate that the coupling between local biochemical interactions and long-range mechanical facilitation is a conserved feature of Rho GTPases partitioning. This is in good agreement with other systems in which Rho GTPase partitioning relies on long-range communication via the actin and the membrane, from polarization in early *Caenorhabditis* *elegans* embryos by cortical flow^84^, to axon branching in neuron via long-range membrane tension propagation^60^, to the winner-take-all competition enabling a single front during migration^27,28^. Long-range mechanochemical interactions represent a conserved mechanism to complement local biochemical interactions for the partitioning of Rho GTPases in cell migration and likely other processes.

## Methods

Our research complies with all relevant ethical regulations.

### Experimental model and subject details

HL-60 cells are from the laboratory of H. Bourne and were recently verified via short tandem repeat profiling^29^. HL-60s were cultured in R10 growth medium, which is RPMI 1640 supplemented with L-glutamine and 25 mM HEPES (Corning; Supplementary Table 1) and containing 10% (v/v) heat-inactivated fetal bovine serum (Gibco). Cultures were kept at a density of 0.2–1.0 million cells per ml at 37 °C/5% CO_2._ HL-60s KO for mSin1 and for mTORC2 were obtained from elsewhere^29^. OptoPI3K cells (iLid–BFP–CAAX, iSH2–GFP, Pak-PBD–mCherry) and opto-LARG cells (iLid-BFP-CAAX, DHPH-ARHGEF1–GFP, AnillinRBD–mCherry) were obtained from elsewhere^31,32^. Opto-PI3K and opto-LARG expressing AnillinRBD–mCherry, Pak-PBD–mCherry, Ezrin–mCherry, Native-Tubby–miRFP650, Tubby-R332HHaloTag, pH-Akt–HaloTag, mSIN1.2–mCherry and pRLCSNAP2 were assembled using a Golden-Gate-based modular cloning toolkit^85^.

HEK293T cells (used to make lentivirus for transduction of HL-60s) were from the University of California, San Francisco (UCSF) cell culture facility (CCLZR076) and were grown in DMEM (Corning) containing 10% (v/v) heat-inactivated fetal bovine serum (Gibco) and maintained at 37 °C/5% CO_2_. All media were 0.22-μm filtered.

Healthy blood specimens from patients were obtained with informed consent according to the institutional review board-approved study protocol at the UCSF (study no. 21-35147). Volunteers were informed not to take ibuprofen, acetaminophen or more than one drink of alcohol within 24 h, class 1 or 2 antihistamines within 5 days, or aspirin within 7 days of blood draw. Volunteer demographics such as age and sex are donor no. 4 (male, age 28 years); no. 21 (male, age 28 years); no. 23 (male, age 24 years); no. 28 (female, age 27 years); and no. 24 (female age 27 years).

### Method details

#### Making virus for transduction of HL-60 and primary T cells

HEK293T cells were used to generate lentivirus and were seeded into six-well plates until approximately 80% confluent. For each transduction, 1.5 μg pHR vector (containing the appropriate transgene), 0.167 μg vesicular stomatitis virus-G vector and 1.2 μg cytomegalovirus 8.91 vector were prepared for transfection using TransIT-293 Transfection Reagent (Mirus Bio). Two days after transduction, virus-containing supernatants were collected and concentrated approximately 40-fold using Lenti-X Concentrator (Clontech). Concentrated viruses were frozen and stored at −80 °C until needed. For HL-60 transductions (see below for T cell transduction), thawed virus was mixed with approximately 0.3 million cells in growth medium supplemented with Polybrene (8 μg ml^−1^) and incubated overnight. Cells expressing desired transgenes were isolated using fluorescence-activated cell sorting (FACS) as appropriate (BD FACSAria Fusion; BD Biosciences).

#### HL-60 differentiation

HL-60s were differentiated into neutrophil-like cells by taking an aliquot of cells in their culturing medium and supplementing with an equal volume of dimethylsulfoxide (DMSO) diluted in RPMI such that the final concentrations were 0.2 million per ml HL-60 cells with 1.3% (v/v) DMSO and 5% (v/v) fetal bovine serum in RPMI.

#### T cell isolation protocol

T cell medium (TCM) was first prepared with Lonza X-VIVO15 (cat. no. 04-418Q), 5% human AB serum (Fisher, cat. no. NC9310328), 10 mM neutralized *N*-acetyl L-cysteine (Sigma-Aldrich, cat. no. A9165) and 1 mM 2-mercaptoethanol (Gibco, cat. no. 21985-023). The medium mix was filtered and stored at 4 °C. Before use with T cells, 40 U ml^−1^ human interleukin (IL)-2 (National Cancer Institute (NCI) BRB preclinical repository) was added in batches of 50 ml and stored at 4 °C.

Blood specimens from patients were obtained with informed consent according to the institutional review board-approved study protocol at the UCSF (study no. 21-35147). Fresh samples of peripheral blood (three tubes, 7 ml each) from healthy adult volunteers were collected via a BD 23-gauge butterfly needle collection set (Fisher SKU: 23-021-022) into 8.5 ml BD Vacutainer ACD Solution A tubes (BD364606). Blood was kept on a shaker at minimum setting and utilized within 2 h of the draw. CD4^+^ T cells were isolated using the Stemcell EasySep Direct Human CD4^+^ T cell Isolation kit (no. 19662) with the Easy50 magnet (no. 18002) according to the manufacturer’s protocol. Cells were spun down at 300*g* for 5 min, resuspended in TCM + 40 U ml^−1^ IL-2 between 1–2 × 10^6^ cells per ml, and then placed in a Corning six-well plate (CAT). For drop-assay use, T cells went through the transduction protocol below; for chemotaxis use they followed the chemotaxis protocol from this point. For all language used in this method section, day 1 is the day of isolation.

#### Transduction of human primary T cells

To integrate the biosensors into the primary T cells, we did the following protocol. T cells were allowed to rest in IL-2 TCM overnight until activation midday on day 2. To activate the T cells, cells were placed in a 24-well plate with approximately 1–1.5 × 10^6^ cells per well in 1 ml of medium. Next, following the manufacturer’s guidelines, we added 25 μl of washed and TCM + 40 U ml^−1^ IL-2 resuspended CD3/28 Dynabeads (Gibco, cat. no. 11131D) to each well. Cells were incubated with beads for 24 h before being transferred to a 24-well plate pre-coated with retronectin (Takara, cat. no. T100B). Retronectin coating was carried out by following the manufacturer’s protocol with approximately 10 μg per well (5 μg cm^−^^2^) used in 0.5 ml of PBS in Falcon non-TC treated 24 wells (cat. no. 0877251). Next, 100 μl of concentrated lentivirus was added to each well of T cells for the Rac–mCherry (Pak-PBD–mCherry) or Rho–GFP (Anillin–GFP) biosensors. Normally, three or four wells of the same condition were used in parallel to have enough cells to sort. Cells were spin-fected by spinning at 460*g* for 1 h at room temperature (25–30 °C), and then placed in a 37 °C 5% CO_2_ incubator for 3 days. On day 6, cells were taken from the plate, Dynabeads were removed with a magnet, then placed in a fresh tube. Cells were then spun down at 300*g* for 5 min, resuspended in fresh TCM with 40 U ml^−1^ IL-2 at approximately 2 × 10^6^ cells per ml. Cells were sorted for positive mCherry fluorescence on a FACSAria Fusion instrument (Rho cells were not sorted). After sorting, cells were spun down and resuspended in fresh TCM + 40 U ml^−1^ of IL-2. Cells were allowed to rest for 2 or more days before use in drop experiments.

#### Microscopy hardware

Imaging depicted in Figs. 1–5 and Extended Data Figs. 1–6 were performed at 37 °C on a Nikon Eclipse Ti inverted microscope equipped with a Borealis beam conditioning unit (Andor), a CSU-W1 Yokogawa spinning disc (Andor), a ×100 PlanApo TIRF 1.49 numerical aperture (NA) objective (Nikon), an iXon Ultra EMCCD camera (Andor) and a laser merge module (LMM5, Spectral Applied Research) equipped with 405, 440, 488 and 561-nm laser lines. All hardware was controlled using Micro-Manager (UCSF).

Optogenetic activation was performed using a LED (470 nm) via a custom DMD (Andor Technology). Illumination intensities were varied by connecting the LEDs to the analogue outputs of a digital-to-analogue converter and setting the LED voltages using serial commands via custom Python code. The microscope is equipped with two stacked dichroic turrets such that samples can be simultaneously illuminated with LEDs and imaged using a 488-nm long-pass dichroic filter (Chroma Technology Corp.)

Imaging depicted in Fig. 8, migrating cells from Fig. 5 and osmotic shocks from Fig. 6 were performed using a Nikon Ti2-E body scope configured with a CrestOptics X-Light V3 confocal spinning disc system, a Lumencor Celesta light engine, Nikon ×10 CFI Plan Apo Lambda objective (for migrating T cells) or CFI Plan Apo VC 60XC WI objective lens, an Okobox temperature and CO_2_ controlled environment, and a Photometrics Kinetix sCMOS camera. The camera was run in a 2 × 2 binning mode with a set exposure time of 200 ms for all channels. All videos are taken with a frame interval of 30 s between exposures unless otherwise indicated. All videos were taken at 37 °C with 5% CO_2_ for the duration of imaging.

#### Preparation of opto-PI3K and opto-LARG cells for confocal imaging

For experiments in which we monitored HL-60s cells by confocal imaging, cells were seeded in a 96-well no. 1.5 glass-bottom plates (Azenta Life Sciences) in R + B imaging medium, which is RPMI 1640 supplemented with L-glutamine and 25 mM HEPES (Corning) and containing 0.2% BSA (endotoxin-free, fatty acid free; A8806, Sigma) or for epithelial cells DMEM (Corning) containing 10% (v/v) heat-inactivated fetal bovine serum (Gibco). For optogenetic activation, cells were illuminated using DMD (see above) at a chosen location (using custom Python code) in a circular pattern of varying size (~2-μm radius for Opto-PI3K, ~1-μm radius for opto-LARG) for a duration of 90 s.

Most experiments were conducted, at least for one replicate each, together with plasma membrane imaging using the membrane dye CellMask to ensure proper recruitment of different biosensors at the plasma membrane and to control for accurate segmentation. For plasma membrane staining, cells were first incubated with ~2–5 μg ml^−1^ of CellMask Deep Red (C10046, Thermo Fisher) for 3 min at 37 °C/5% CO_2_. Cells were then pelleted at 300*g* for 3 min and resuspended in R + B imaging medium (RPMI + 0.2% BSA). For imaging using HaloTag (Fig. 6a,b,e and Extended Data Figs. 1e,f and 7h,j), cells were stained with 100 nM JF646X for 10 min before being pelleted at 300*g* for 3 min and resuspended in R + B imaging medium (RPMI + 0.2% BSA).

#### Osmotic shock

For osmotic shock experiments cells were stained using CellMask Deep Red (see above) and seeded in a 96-well no. 1.5 glass-bottom plates (Azenta Life Sciences) in imaging medium, which is either RPMI 1640 (for HL-60s) supplemented with L-glutamine and 25 mM HEPES (Corning) and containing 0.2% BSA (endotoxin-free, fatty acid free; A8806, Sigma) or in the case of epithelial cells DMEM (Corning) containing 10% (v/v) heat-inactivated fetal bovine serum (Gibco). Deionized water was then manually added at different amount (50–80% of the final volume, depending) while imaging.

#### Micropipette aspiration

Micropipette aspiration experiments depicted in Figs. 2, 3 and 6 were performed using a micromanipulator (Narishige MM-188NE) mounted on Nikon Eclipse Ti (detailed description above) using a custom 3D printed mount. The micropipettes (Sunlight Medical, SRP-05P-35) were mounted and secured onto the holder using polystyrene foam. Aspiration was performed manually using the CellTram 4r air (Eppendorf). Micropipettes were coated for 10 min with RPMI or DMEM (for epithelial cells) containing 5% BSA to prevent cells sticking to it. The medium was then emptied, and the pipette filled with R + B imaging medium (RPMI or DMEM + 0.2% BSA) for approximately 30–60 min. Cells were plated on round MateK dishes in R + B imaging medium (RPMI or DMEM + 0.2% BSA). Aspiration was monitored live using either brightfield or the membrane dye CellMask Deep Red (see above for staining protocol).

#### Stable blebs

For stable blebs generation (Figs. S5 and 6g, h), cells were seeded on imaging plates pre-coated with poly-L-lysine (Sigma-Aldrich, P4832) (coated for 5 min and washed three times with PBS). Imaging medium used was either R10 growth medium (for HL-60s), which is RPMI 1640 supplemented with L-glutamine and 25 mM HEPES (Corning) and containing 10% (v/v) heat-inactivated fetal bovine serum (GibcoA) together with 10 μM latrunculin B (Sigma-Aldrich, 76343-94-7) or for 293Ts, DMEM (Corning) containing 10% (v/v) heat-inactivated fetal bovine serum (Gibco) together with 10 μM latrunculin B (Sigma-Aldrich, 76343-94-7).

#### Pharmacological treatments

For inhibition of cellular protrusion (Extended Data Fig. 2), cells were seeded in imaging medium (RPMI 1640 supplemented with L-glutamine and 25 mM HEPES (Corning) and containing 0.2% BSA (endotoxin-free, fatty acid free; A8806, Sigma), supplemented with either 10 μM latrunculin B (Sigma-Aldrich, 76343-94-7) or 100 μM CK666 (Sigma-Aldrich, 442633-00-3). For inhibition of the actin cortex during osmotic shock (Extended Data Fig. 3), cells were seeded in imaging medium (as described above) supplemented with 10 μM latrunculin B (Sigma-Aldrich, 76343-94-7). H_2_O also containing 10 μM latrunculin B was then added during imaging to generate a hypo-osmotic shock without diluting the drug. Inhibition of mTOR kinase activity (Extended Data Fig. 4) was performed by treating cells with 1–5 μM KU-0063794 (Selleck Chemical, 50-837-4) for 30–40 min before imaging. For inhibition of contractility (Fig. 4), cells were seeded in imaging medium supplemented with 10 μM blue light resistant para-amino-blebbistatin (Cayman Chemical, 22699). For PI4K inhibition (Extended Data Fig. 7), cells were seeded in imaging medium supplemented with 200 nM GSK-A1 (MedChemExpress, HY-125118) for 30 min before imaging. For PI5K inhibition cells were seeded in imaging medium supplemented with 5 μM PI5K inhibitor PIP5K1C-IN-1 (MedChemExpress, HY-163476) for 30 min before imaging. For PIP_2_ sequestration experiments, cells were seeded in imaging medium supplemented with 10 mM neomycin (Thomas Scientific, C791M46) for 20 min before imaging. For PI3Kδ/γ inhibition (Fig. 5 and Extended Data Figs. 7 and 10), cells were seeded in imaging medium supplemented with 1 μM duvelisib (MedChemExpress, NC2099793). For inhibition of ROCK activity in human primary cells (Fig. 8), cells were seeded in T cell starvation medium consisting of Lonza X-VIVO15 (cat. no. 04-418Q), 0.4% BSA (Sigma-Aldrich, 9048-46-8), 10 mM neutralized *N*-acetyl L-cysteine (Sigma-Aldrich, cat. no. A9165) and 1 mM 2-mercaptoethanol (Gibco, cat. no. 21985-023). The medium mix was filtered and stored at 4 °C. Before use with T cells, 40 U ml^−1^ human IL-2 (NCI BRB preclinical repository) was added in batches of 50 ml and stored at 4 °C. T cell starvation medium was supplemented with 20 μM Y-27632 (MilliporeSigma, 68-800-01MG) approximately 20 min before imaging. For inhibition of Gαi signalling (Fig. 8), cells were incubated overnight in media (starvation for CCL19 drop experiments and culture medium for migration experiments) supplemented with 1 μg ml^−1^ PTX (Thermo Fisher Scientific, PHZ1174). For mTORC2 inhibition cells 10 μM KU-0063794 (Selleck Chemical, 50-837-4) was added approximately 20 min before imaging.

#### CCL19 drop assay

For CCL19 drop assay (Fig. 8 and Extended Data Fig. 10), human primary cells were serum starved overnight in serum starvation medium consisting of Lonza X-VIVO15 (cat. 04-418Q), 0.4% BSA (Sigma-Aldrich, cat. no. 9048-46-8), 10 mM neutralized *N*-acetyl L-cysteine (Sigma-Aldrich, cat. no. A9165) and 1 mM 2-mercaptoethanol (Gibco, cat. no. 21985-023). Media mix was filtered and stored at 4 °C. Before use with T cells, 40 U ml^−1^ human IL-2 (NCI BRB preclinical repository) was added in batches of 50 ml and stored at 4 °C. Cells were then seeded on 96-well no. 1.5 glass-bottom plates (Azenta Life Sciences) pre-coated for 30 min with human fibronectin at 1 mg ml^−1^ (Sigma-Aldrich, F0895). During imaging, starvation medium containing 50 mM CCL19 (R&D Systems, 361MI025CF) was added into the well after 90 s of imaging. To avoid unwanted dilution, drugs such as Y27 and PTX were also added to the CCL19 mix at the same concentration as used during cell plating.

#### T cell migration experimental preparation

On the day of experiment, an ibidi six-channel device was assembled by sticking down a sticky-Slide VI 0.4 (ibidi, cat. no. 80608) to a bare 1.5H full coverslip (ibidi, cat. no. 10812). Care was taken not to press too hard on the device, and a P1000 tip was taken to gently press out air bubbles between the adhesive and glass. The device was placed at 37 °C until use to set. Devices that were not fully used were emptied, the used channel marked and put in the fridge at 4 °C for use of unused channels within a week.

Unactivated human T cells were allowed to culture in TCM + 40 U ml^−1^ IL-2 until day 5–6 before use in the chemotaxis assay. On the day of the assay, 5 μg ml^−1^ of Hoechst 3334 (Invitrogen, cat. no. H3570) was added directly to the culture medium for 15 min at room temperature. Cells were then spun down, resuspended in fresh in TCM + 40 U ml^−1^ IL-2 at around 5 × 10^6^ cells ml^−1^ and put on ice.

Next, we prepared a collagen solution at a 2× concentration to mix with the cells. All steps were carried out on ice until indicated, and the ibidi chip was placed on to an icepack block to get cold before use. First, 18 μl Type I bovine collagen solution (Nutragen, cat. no. 5010-50ML) was pipetted into an ice-cold 1.5-ml Eppendorf tube with a positive displacement pipette. Next, 12 μl of FITC-collagen (Sigma, cat. no. C4361-10ML) was added and mixed with a positive displacement pipette on ice. Following a decent mixture of the two, 14 μl of ice-cold MilliQ filtered water and 10 μl of ice-cold 55 mM NaOH were added and mixed with a normal pipette on ice. The entire 60 μl collagen solution was mixed 1:1 with the chilled concentrated cell solution above and then 100 μl of this collagen/cell mix was immediately injected into a channel of the cold ibidi device. Usually two channels were loaded (control and drug) and the device was left on ice until both channels were loaded. Care was taken to ensure that an even amount of collagen/cell mix was distributed to each side of the channel before the entire device was placed on top of a heat block inside of a 37 °C, 5% CO_2_ incubator. The device was allowed to incubate for 30 min before use in microscopy experiments.

For imaging, the ibidi channel device was moved to the microscope described in microscopy hardware for Fig. 7. While incubating at 37 °C and 5% CO_2_, 50 μl of TCM + 40 U ml^−1^ IL-2 was added to one side of the channel and 50 μl of TCM + 40 U ml^−1^ IL-2 + 5–10 μg ml^−1^ 10 kDa AF647-dextran (Invitrogen, cat. no. D22914) + 200 nM recombinant human CCL19 (Biotechne, cat. no. 361-MI) was added to the other side. Imaging occurred on the edge of the channel port that had the CCL19 added to it.

When drugs were used in this assay, the correct and final treatment concentration was added to all parts of the assay so that the concentration listed in the text was present in the collagen, medium and chemoattractant mixes.

### Quantification and statistical analysis

Data acquired in this study were not randomized.

#### Image analysis

Fiji (NIH)^86^, Excel (Microsoft), custom Python code and Prism (GraphPad Software) were used for image analysis.

For all analysis relying on cell segmentation, each frame was manually verified and the occasional mis-segmented frame was excluded from analysis.

For mean Rac activity (Pak-PBD), Rho activity (AnillinRBD), mSIN1 or Ezrin intensity in opto-PI3K and opto-LARG cells (Figs. 1–6 and Extended Data Figs. 1–6) cells were automatically segmented using CellPose^87^. Every segmentation was manually checked for discrepancy. We then generated kymographs by segmenting the cell body through either the CellMask channel and finding the three-pixel wide boundary pixels that best capture the membrane of the CellMask channel. We then confirmed that our segmentation pipeline could function equally well using directly the Rac, Ezrin or Rho biosensors signal, thus avoiding potential bleed through from the membrane stain. The segmented cell is unravelled and AnillinRBD, Pak-PBD or Ezrin intensity is measured and stored in an array. For cells with poor automatic segmentation due to low signal, cells were manually measured by taking a region of interest at the plasma membrane at the site of activation or directly opposite accordingly. For kymographs arrays were stacked to show the evolution of the signal over time. For line graphs, the array was then divided into four separate quadrants (front, sides and back), aligned to the opto-PI3K input which we defined as the cell front. Mean fluorescence per quadrant was then computed for each quadrant of each cell. All measurements were then normalized per cell to steady state. Occasional outlier data points (usually due to a single frame of a video being mis-segmented) were manually curated from the dataset after confirmation of problematic segmentation (by watching side-by-side segmented outlines versus raw images at every time points). Image segmentation code utilized the Python package Scikit-Image^88^.

For plots of Rho activity during osmotic shock and for plots of Rho activity during micropipette aspiration assays we used a custom Fiji macro which uses the membrane channel (CellMask) to create a mask of the cell membrane which is then used to measure the mean AnillinRBD levels at the cell membrane. All measurements were then normalized per cell to steady state. For micropipette aspiration in Figs. 2 and 6 and Extended Data Figs 3, 4 and 8) we used 30 s before aspiration for steady state (using the first time point for normalization) and 30 s of active aspiration.

Mean Rac activity (Pak-PBD) and PIP2 levels in blebs during opto-contraction (Figs. 3 and 4 and Extended Data Fig. 6), were acquired by manually segmenting blebs using the membrane signal. We then backtracked in time to measure the fluorescence at the same patch of membrane before it blebbed to obtained steady-state measurements. For cells without blebs (Fig. 4, grey), we measured a Rac activity at the patch of membrane directly opposite of the opto-light.

For Fig. 4. We measured bleb angle in relation to the activating light using the angle tool in Fiji as well as record when blebs first appear in response to light. Angles were then computed using Python and Scikit-Image^88^.

For stable blebs, in Extended Data Figs. 5 and 6, we measured mean Pak-PBD activity at blebs and in an adjacent patch of membrane as reference. File names for control and PI3Ki cells were randomized, and analysis was performed blind.

For line-scans in Figs. 3 and 4 and Extended Data Fig 7, the front and back of the cells was defined by the direction of cell migration. A line-scan of 3-pixel thickness was then drawn from the back of the cell until the cell front (as represented by dotted lines in Figs. 3g or 5e). Fluorescence intensity was then measured along the line and normalized across 30 sections (to account for cell length not being constant across cells). Only one measurement was taken for each migrating cell to avoid over-representing some cells in the final plot.

For Extended Data Fig. 6 we used a custom Fiji macro to measure the ratio of the MPAct and the membrane channel (CellMask) using a blurred membrane channel as a mask to only perform the ratio near the plasma membrane. We then measured the MPAct/Membrane ratio at the cell front and back (defined by the location of the opto-light) over time. We then normalized every cell to their steady state.

For Fig. 8, we measured mean Rac activity at the cell front by manually identifying cell protrusions using a fluorescence *z*-stack together with brightfield imaging. We then backtracked pre-CCL19 drop to measure Rac activity at the same patch of membrane to obtained steady state measurements. When a front could not easily be identified, we measured Rac activity at the membrane of the mid-plane of the cell. Because only some of cells express CCR7 (approximately 7–80%), the receptor for CCL19, we only included in the analysis cells that displayed a cell shape change in response to CCL19 (such as protrusion, contraction, blebbing and migration). For Rho measurements, we used a similar approach but measuring the back of the cell (as defined by being opposite of the cell front) which was typically located at the bottom z-plane at the interface between imaging dish and the cells.

For Fig. 8. The T cell chemotaxis image analysis presented in this work was achieved via the use of both Fiji (ImageJ) and Python. Images were pre-processed in Fiji by max projecting the *z*-stack of nuclei taken to simplify the downstream Python analysis. Nuclei were segmented using StarDist, and labelled nuclei were linked into tracks using the scikit-image and TrackPy packages. Tracks were then analysed for their step-wise linear velocity and velocity *X* and *Y* bias over time. All Python analysis for each graph will be provided as open-source code in the form of both scripts and Jupyter notebooks on Github by the time of publication.

#### Statistical analysis

No statistical methods were used to pre-determine sample sizes but our sample sizes are similar to those reported in previous publications^26–38^. Most statistical tests did not assume normal distribution (for example, Wilcoxon paired Student’s *t*-test). For all statistical analysis, PRISM 9 (GraphPad Software) was used. Statistical details can be found in the legend of each figure. *N* represents number of independent biological replicates. Pooled independent experiments are used in dot plots.

### Reporting summary

Further information on research design is available in the Nature Portfolio Reporting Summary linked to this article.

## Online content

Any methods, additional references, Nature Portfolio reporting summaries, source data, extended data, supplementary information, acknowledgements, peer review information; details of author contributions and competing interests; and statements of data and code availability are available at 10.1038/s41556-026-01965-1.

## Supplementary information

Supplementary Information

Reporting Summary

Peer Review File

Supplementary Video 1 Time-lapse confocal images of HL-60 cells expressing opto-PI3k (Opto-Rac) and Rac biosensor (Pak-PBD) showing localized Rac activation upon light activation. Related to Fig. 1c,d. Scale bar, 10 μm.

Supplementary Video 2 Time-lapse confocal images of HL-60 cells expressing opto-PI3k (Opto-Rac) and Rho biosensor (AnillinRBD) showing that light-induced Rac activation elicits a rapid long-range increase in Rho activation at the opposite side of the cell. Related to Fig. 1f,g. Scale bar, 10 μm.

Supplementary Video 3 Time-lapse confocal images of HL-60 cells expressing Rho biosensor (AnillinRBD) before and during osmotic shock performed by directly adding water to the imaging media (70% final). This shows that Rho activation increase in response to increasing membrane tension by osmotic shock. Related to Fig. 2c,d. Scale bar, 10 μm.

Supplementary Video 4 Time-lapse confocal images of an HL-60 cell expressing Rho biosensor (AnillinRBD) and stained with membrane marker CellMask before and during micropipette aspiration assay, showing that Rho activation increase in response to mechanically increasing membrane tension. Related to Fig. 2f,g. Scale bar, 10 μm.

Supplementary Video 5 First video: Time-lapse confocal images of a Rictor KO HL-60 cell expressing opto-PI3k (Opto-Rac) and Rho biosensor (AnillinRBD) showing that light-induced Rac activation elicits no increase in Rho activation at the opposite side of the cell. Related to Fig. 3c,d. Scale bar, 10 μm. Second video: Time-lapse confocal images of a Rictor KO HL-60 cell expressing Rho biosensor (AnillinRBD) before and during osmotic shock performed by directly adding water to the imaging media (70% final) showing minimal change in Rho activation in response to osmotic shock. Related to Extended Data Fig. 4f,g. Scale bar, 10 μm. Third video: Time-lapse confocal images of a Rictor KO HL-60 cells expressing Rho biosensor (AnillinRBD) and stained with membrane marker CellMask before and during micropipette aspiration assay, showing no noticeable change in Rho activity in response to mechanically increasing membrane tension. Related to Extended Data Fig. 4i,j. Scale bar, 10 μm.

Supplementary Video 6 Time-lapse confocal images of an HL-60 cell expressing opto-LARG (Opto-Rho) and the Rac biosensor (Pak-PBD) showing that Rho activation leads to long-range Rac activation at the opposite end of the cell, coinciding with another morphological change— blebbing. Related to Fig. 4b–e. Scale bar, 10 μm.

Supplementary Video 7 Time-lapse confocal images of an HL-60 cell expressing opto-LARG (Opto-Rho) and Ezrin–mCherry showing that upon local Rho activation, ezrin accumulated at the site of contraction and was depleted at the opposite side of the cell. Related to Fig. 4g,h. Scale bar, 10 μm.

Supplementary Video 8 First video: time-lapse confocal images of an HL-60 cell expressing opto-LARG (Opto-Rho) and PIP2 biosensor (Tubby-R332H-HaloTag) showing that local Rho activation leads to a marked increase in PIP_2_ level in blebs. Related to Fig. 5a,b. Second video: time-lapse confocal images of an HL-60 cell expressing opto-LARG (Opto-Rho) and PIP2 biosensor (Tubby-Native-miRFP650). Related to Fig. 5c,d. Scale bar, 10 μm.

Supplementary Video 9 Time-lapse confocal images of migrating differentiated HL-60 cells expressing either PIP2 biosensors (Tubby-R332H-HaloTag on the left and Native-Tubby-miRFP650 on the right). Related to Fig. 5e,f. Scale bar, 10 μm.

Supplementary Video 10 Mechanochemical model of Rac and Rho partitioning following local Rac activation at the cell front. Related to Fig. 7f.

Supplementary Video 11 Time-lapse confocal images of a primary T cell expressing the Rac biosensor (Pak-PBD) before and after adding 25 nM CCL19. Related to Fig. 8c–e.

Supplementary Video 12 Ex vivo assay for human primary T cell chemotaxis. Cells were premixed with bovine dermal collagen and placed into linear channels. After the collagen set, medium was added to one side of the channel (TCM) and medium with human CCL19 (100 ng total) and 10 μg ml^−1^ of Dextran10k-AF647 (Dex647) was added to the other. Average linear velocity of all tracks at each time point was recorded. In order showing control cells, cells treated with Y27 and cells treated with PTX. Related to Fig. 8j–o.

Supplementary Table 1 Resources table.

Supplementary Table 2 Light microscopy reporting table.

Supplementary Table 3 Parameters for the model.

## Source data

Source Data Source data.

## Data Availability

All data supporting the findings of this study will be made available within the manuscript. All other data supporting the findings of this study can be obtained from the corresponding authors upon reasonable request. Source data are provided with this paper.

## Code availability

Unique code generated for this study can be found on GitHub at https://github.com/VirtualEmbryo/mechanochemical_polarization and Zenodo at 10.5281/zenodo.19591544 (ref. ^89^).
