# Supplementary material for: Long-range mutual activation establishes Rho and Rac polarity during cell migration
Source: Nat Cell Biol. 2026 Jun 10;28(6):1244–57. doi: 10.1038/s41556-026-01965-1 (PMC13279283; doi:10.1038/s41556-026-01965-1)
Supplement: Supplementary file 1 — Supplementary Information [file 41556_2026_1965_MOESM1_ESM.pdf]

nature cell biology

Supplementary information https://doi.org/10.1038/s41556-026-01965-1

Long-range mutual activation establishes Rho and Rac polarity during cell migration

In the format provided by the authors and unedited

Supplementary Text

Long range mutual activation establishes Rho and Rac polarity during cell migration

Henry De Belly, Andreu Fernández Gallén, Evelyn Strickland, Dorothy C. Estrada, David Sanchez Godinez, Eric Neiva, Patrick J. Zager, Tamas L. Nagy, Janis K Burkhardt, Hervé Turlier, Orion D. Weiner

1 Model hypotheses

Rho GTPases have emerged as key components of the cell polarization machinery. Among them, Rho and Rac are well-known for their antagonistic and inhibitory relationship, often modeled through reaction-diffusion equations [1]. A well-established paradigm for cell polarization is the concept of wave-pinning [2], where bistability of a single RhoGTPase arises from the interplay between a degradation term and a non-linear activation term, combined with overall mass conservation. Recent models have also incorporated the experimentally observed dependence of Rho on membrane tension [3, 4].

In our model, we propose an alternative polarization mechanism to wave-pinning, in which we neglect the shuttling between active and inactive forms of Rho GTPases. Instead, we model the local mutual inhibition between Rho and Rac [5] as a bistable switch, which naturally exhibits both monostable and bistable regimes depending on the parameter set [6], and couple it cortical and membrane mechanics to enable cell polarization, based on our experimental results. Similar to previous works [3, 4], and in agreement with our experimental findings, we assume that increased membrane tension promotes RhoA activation.

Our mechanical model is based on previous work by some of the authors [7] and integrates both the actin cortex and the plasma membrane. In this model, membrane tension effectively acts as a tangential elasticity (membrane folding/unfolding), while the cortex is represented on considered timescales as a viscous, contractile layer [8, 9, 10, 11, 7]. Their mechanical interaction is implemented through tangential viscous friction, which slows their relative tangential movement. This leads to effective diffusive propagation of membrane tension along the cell surface [12, 13], which homogenizes after perturbations over timescales longer than several minutes, in accordance with our previous experimental results in neutrophils [7].

In our current work, we refine this model by explicitly accounting for the spatiotemporal variations in membrane-cortex attachment (MCA) surface density–in particular of ezrin– which exchanges with its inactive form in the cytoplasm and can be advected by cortical. This variable and inhomogeneous membrane-cortex attachment modulates spatiotemporally the relative friction between the membrane and cortex. Experimentally, we observe that bleb-like protrusions are enhanced in regions of low MCA, and that the subsequent local recruitment of Rac generates protrusions. To implement these couplings, we define a switch-like dependence of protrusion velocity with local Rac concentration and a modulation of active Rac recruitment upon MCA depletion. Conversely, we also experimentally report a recruitment of active RhoA upon membrane tension increase, which is similarly accounted for in our model through a switch-like modulation of RhoA production by membrane tension.

In the following text, we first present the local mutual inhibition biochemical model for Rho and Rac and analyzing its response to external stimulation, then we study an extension including wave-pinning dynamics and demonstrate the hypersensitivity of such class of models to external stimulation (e.g. optogenetic activation of Rho or Rac). We then introduce ou alternative toy mechanochemical model, where we first derive coupled mechanical equations for the membrane and cortex in one dimension that we combine with the Rho-Rac mutual inhibition model and MCA dynamics to investigate the full mechanochemical system's response to external stimulation of Rho or Rac. Finally, we propose a generalization of our integrated mechanochemical model to two dimensions, that we solve using finite-element modeling on a undeformable sphere, showing that it leads to qualitatively similar results than in 1D.

2 Biochemical model of Rho and Rac

In this section, we first describe the coupled dynamics of Rac (R) and Rho (ρ), modeled locally via ordinary equations describing their activation modulated by mutual inhibition and their inactivation, in a similar fashion as a toggle-switch [14]. In contrast to wave-pinning models, which account explicitly for the shuttling between an active form of the protein in the membrane and an inactive form in the cytosol, and rely importantly on a limited total amount of RhoGTPase in the cell, we assume here the membrane in contact with a chemiostat of inactive proteins in the cytosol. As such, we assume effectively a constant cytosolic concentration, that is integrated within the activation rates. This simplifying hypothesis is justified by former experimental work, which demonstrated that in neutrophils, a diffusion-based inhibition or local sequestration mechanism was not sufficient to explain polarization [15].

The two ordinary equations read

$$\frac{\partial R}{\partial t} = \alpha_0 \, \frac{k_R^2}{k_R^2 + \rho^2} + s_R - d_R \, R, \tag{1}$$

$$\frac{\partial \rho}{\partial t} = \beta_0 \, \frac{k_\rho^2}{k_\rho^2 + R^2} + s_\rho - d_\rho \rho, \tag{2}$$

where α_0 and β_0 are the basal activation rates of Rac (R) and Rho (ρ), respectively; k_R and k_ρ set the switch threshold values for R and ρ. We introduce source terms for Rac and Rho s_R and s_ρ, for any optogenetic or chemotaxis activation of a GPTase.

2.1 Non-dimensionalized equations

We non-dimensionalize previous equations defining dimensionless protein concentrations

$$\bar{\rho} = \rho / k_R \tag{3}$$

$$\bar{R} = R / k_\rho \tag{4}$$

and dimensionless activation rates

$$\bar{\alpha}_0 = \frac{\alpha_0}{k_\rho \, d_R}, \tag{5}$$

$$\bar{\beta}_0 = \frac{\beta_0}{k_R \, d_\rho}. \tag{6}$$

The resulting dimensionless equations for the biochemical equations are

$$\frac{1}{d_R} \frac{\partial \bar{R}}{\partial t} = \bar{\alpha}_0 \, \frac{1}{1 + \bar{\rho}^2} + \bar{s}_R - \bar{R}, \tag{7}$$

$$\frac{1}{d_\rho} \frac{\partial \bar{\rho}}{\partial t} = \bar{\beta}_0 \, \frac{1}{1 + \bar{R}^2} + \bar{s}_\rho - \bar{\rho}, \tag{8}$$

2.2 Steady-state solutions

Nullclines and fixed points

Steady-state equations ($\partial / \partial t = 0$) define the nullclines

$$0 = \bar{\alpha}_0 \, \frac{1}{1 + \bar{\rho}^2} - \bar{R} \equiv f(\bar{R}, \bar{\rho}), \tag{9}$$

$$0 = \bar{\beta}_0 \, \frac{1}{1 + \bar{R}^2} - \bar{\rho} \equiv g(\bar{R}, \bar{\rho}). \tag{10}$$

The intersection of the nullclines $f(\bar{R}, \bar{\rho})$, $g(\bar{R}, \bar{\rho})$ define the possible steady-state solutions as function of the values of activation rates $(\bar{\alpha}_0, \bar{\beta}_0)$. As illustrated on Supplementary Text Fig. 1, there are two possible scenarios. Either the nullclines have one intersection - or fixed - point, which is always a stable solution, and corresponds either to a (high Rac/low Rho), or (low Rac/high Rho) situation; or the curves intersect on three fixed points, two of which are stable solutions while the intermediate one is unstable. In the

former case, the system is said monostable, while in the latter, the system displays a bistable behavior where both (high Rac/low Rho) and (low Rac/high Rho) coexist as possible stable solutions.

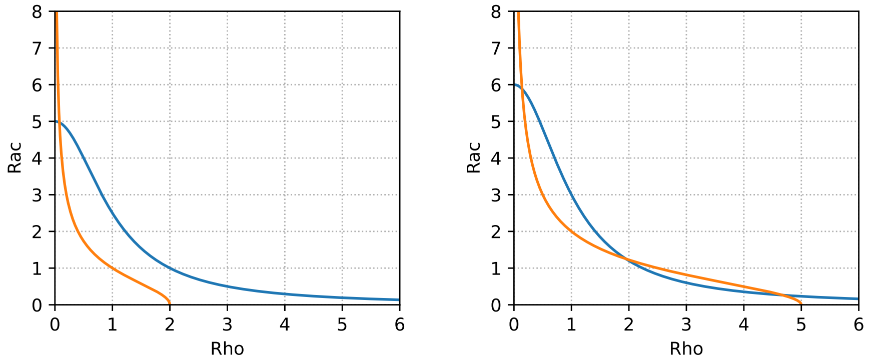

Supplementary Text Figure 1: Plot of eqs (9) and (10) for the values $\bar{\alpha}_0 = 5$ $\bar{\beta}_0 = 2$ and $\bar{\alpha}_0 = 6$ $\bar{\beta}_0 = 5$ respectively. There is a steady state solution for R and ρ where this two lines cross.

Phase diagram

We can compute the regions of parameters $(\bar{\alpha}_0, \bar{\beta}_0)$ of monostability and bistability by looking for the roots $(\bar{R}^*, \bar{\rho}^*)$ of the steady-state equations $(f(\bar{R}, \bar{\rho}) = 0,\ g(\bar{R}, \bar{\rho}) = 0)$, while ensuring that these roots are stable fixed points $(\partial_{\bar{R}} f(\bar{R}, \bar{\rho})|_{\bar{R}^*, \bar{\rho}^*} < 0\ \partial_b g(\bar{R}, \bar{\rho})|_{\bar{R}^*, \bar{\rho}^*} < 0)$.

The transition lines between monostable and bistable stability regions are obtained when

$$f(\bar{R}, \bar{\rho}) = 0, \quad g(\bar{R}, \bar{\rho}) = 0, \quad \partial_{\bar{R}} f(\bar{R}, \bar{\rho}) = 0, \quad \partial_{\bar{\rho}} g(\bar{R}, \bar{\rho}) = 0, \tag{11}$$

which leads to the following polynomial equations for $(\bar{R}, \bar{\rho})$

$$0 = -\bar{\alpha}_0 + \bar{R}\left(1 + \bar{\beta}_0^2\right) - 2\,\bar{R}^2\bar{\alpha}_0 + 2\,\bar{R}^3 - \bar{R}^4\bar{\alpha}_0 + \bar{R}^5 \tag{12}$$

$$0 = -\bar{\beta}_0 + \bar{\rho}\,\left(1 + \bar{\alpha}_0^2\right) - 2\,\bar{\rho}^2\,\bar{\beta}_0 + 2\,\bar{\rho}^3 - \bar{\rho}^4\,\bar{\beta}_0 + \bar{\rho}^5 \tag{13}$$

The phase diagram is plotted on Supplementary Text Fig. 2 as function of $\bar{\alpha}_0$ and $\bar{\beta}_0$.

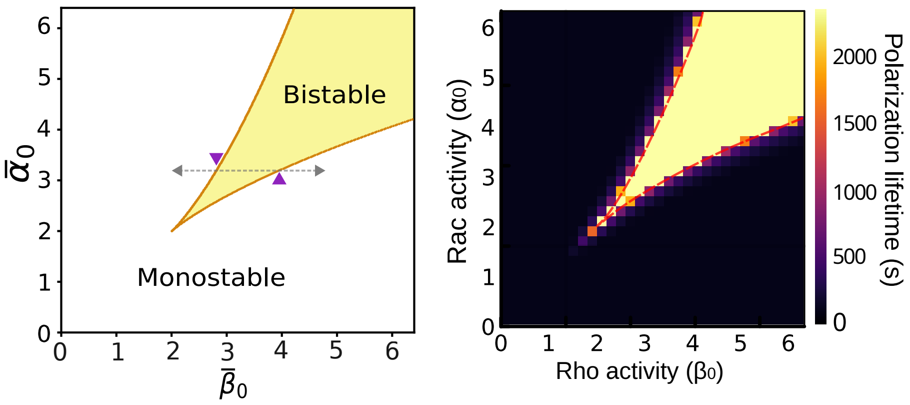

Supplementary Text Figure 2: Phase diagram of mono and bi-stability regions of Rho and Rac, as function of their normalized activation rates $\bar{\beta}_0$ and $\bar{\alpha}_0$. The transition curves are in orange. Plotted in grey arrow the hysteresis represented in Figure 3 for the line $\bar{\alpha} = 3.2$ and represented and with purple triangles each transition point for the hysteresis. We can reproduce the validity of this bi-stability phase diagram with simulations in which we see how inside the bistable region polarized systems never lose polarization, the colorbar represents polarization life-time in seconds.

The transition from low to high Rho/Rac and conversely exhibits a hysteresis, that we illustrate on Supplementary Text Fig. 3. At a given value $\bar{\alpha}_0 = 3.2$, if one increases $\bar{\beta}_0$ from a low value ~ 0, the system will switch from (high Rac, low Rho) to (low Rac, high Rho) only when $\bar{\beta}_0$ reaches the second transition curve at $\bar{\beta}_0 \simeq 3.92$. Traversing the parameter space in the reverse direction, starting from $\bar{\beta}_0 \sim 6$ and decreasing its value the transition from (low Rac, high Rho) will happen this time when goes below $\bar{\beta}_0 \simeq 2.82$

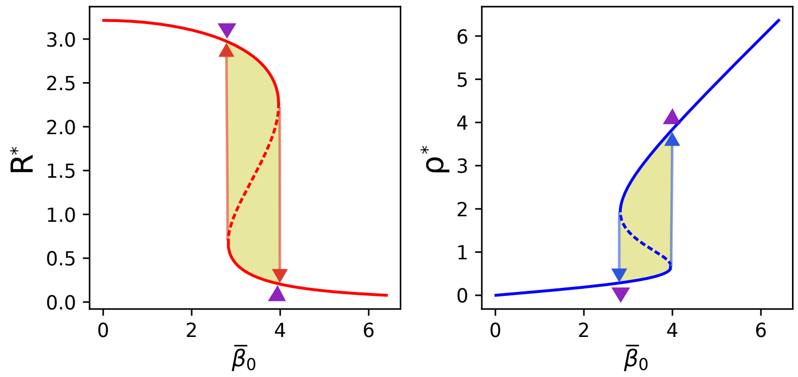

Supplementary Text Figure 3: Steady-state solutions of Rac $\bar{R}^*$ and Rho $\bar{\rho}^*$ as function of $\bar{\beta}_0$ ($\bar{\alpha}_0 = 3.2$ being constant). The solid lines represent stable solutions while the dotted line represents unstable solutions. The hysteresis is materialized through arrows, which delimits the bistability region in yellow $\bar{\beta}_0 \in [2.82, 3.92]$. The purple triangles pointing up and down correspond to the transition line points depicted on Supplementary Text Fig. 2.

2.3 Biochemical model with external stimulation: hysteresis and sensitivity to initial conditions

To model the optogenetic stimulation (or inhibition) of the two GTPase activity, we add source/stimulation (i.e. forcing) terms $\bar{s}_R$, $\bar{s}_\rho$ to our biochemical model:

$$\frac{1}{d_{\bar{R}}}\frac{\partial \bar{R}}{\partial t} = \bar{\alpha}_0 \frac{1}{1+\bar{\rho}^2} + \bar{s}_R - \bar{R}, \quad (14)$$

$$\frac{1}{d_{\bar{\rho}}}\frac{\partial \bar{\rho}}{\partial t} = \bar{\beta}_0 \frac{1}{1+\bar{R}^2} + \bar{s}_\rho - \bar{\rho}. \quad (15)$$

Starting from a given stationary stable state in the bistable region, the external (possibly transient) activation (or inhibition) of one GTPase can make the stationary solution switch to the other stable state. This switch depends on the initial solution and on the type and amplitude of the forcing, which is a manifestation of the hysteresis, as illustrated on Supplementary Text Fig. 4.

The figure 4 illustrates how Rac behaves in response to an external stimulation under distinct basal production rates:

- In the monostable parameter regime (left panel), Rac always converges to the same steady-state value, regardless of the external activation of Rac or Rho (i.e., regardless of the values of $\bar{s}_R$ or $\bar{s}_\rho$).
- In the bistable parameter regime (center and right panels), Rac can switch from one stable steady-state value R^* to the other through transient activation of either Rac or Rho (via $\bar{s}_R$ or $\bar{s}_\rho$ respectively).

In the bistable regime, if the system starts at the lower steady-state value of Rac, applying a sufficiently strong Rac activation ($\bar{s}_R$) can induce a switch to the higher Rac state (center panel, Supplementary Text Fig. 4). However, if the system is initially in the higher Rac state, further Rac activation will not lead to a switch. In this case, transitioning back to the lower state requires Rho activation ($\bar{s}_\rho$), which inhibits Rac and allows the system to fall to the lower stable solution (third panel, Supplementary Text Fig. 4).

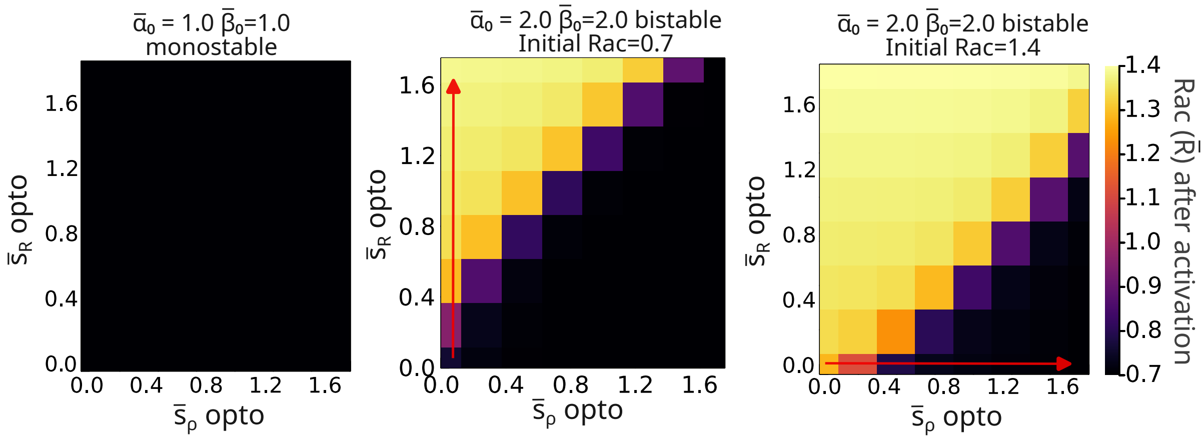

Supplementary Text Figure 4: Heatmap of adimensional Rac concentration $\bar{R}$ at the end of the simulation as a function of Rac and Rho stimulation intensities $\bar{s}_R$ and $\bar{s}_\rho$ in a point-like system. In the bistable regime, the outcome depends on the initial value of $\bar{R}$. During the simulation, localized Rac and Rho stimuli are applied at the same spatial point for 100 seconds. After stimulus removal, the system relaxes, and the final value of $\bar{R}$ is plotted. Arrows indicate the directions of the most effective stimuli for switching Rac from the zero state $\bar{s}_R = \bar{s}_\rho = 0$.

In summary, in a purely biochemical model, the ability to switch between stable states depends strongly on the initial conditions and on which GTPase is activated. Activating only one GTPase may therefore be insufficient to induce a state transition, which limits robustness as a model of cell polarization. Consistent with this, experiments in which cells are prevented from changing tension (drug treatment) indicate that, when only local inhibition is available, optogenetic activation does not reliably trigger polarization, suggesting that cells lie outside the narrow initial-condition regime required for local-inhibition–driven polarization. Motivated by this limitation, we next tested a more classical wave-pinning model as an alternative biochemical mechanism.

2.4 Spatially extended biochemical model

The previous model describes GTPase dynamics at a single point in space, without accounting for their spatial distribution within the cell. To incorporate spatial effects, a mechanism of transport must be introduced. As a first step, we consider the simplest form of transport: the diffusion of GTPases along the cell membrane.

$$\frac{1}{d_{\bar{R}}}\frac{\partial \bar{R}}{\partial t} = \bar{\alpha}_0 \frac{1}{1+\bar{\rho}^2} + \bar{s}_R - \bar{R} + \bar{D}_R \nabla^2 \bar{R}, \tag{16}$$

$$\frac{1}{d_{\bar{\rho}}}\frac{\partial \bar{\rho}}{\partial t} = \bar{\beta}_0 \frac{1}{1+\bar{R}^2} + \bar{s}_\rho - \bar{\rho} + \bar{D}_\rho \nabla^2 \bar{\rho}. \tag{17}$$

Adding diffusion terms to the biochemical system in the bistable regime, and initializing it from a polarized state (with front and back corresponding to steady-state solutions), results in a polarization front that gradually dissipates over time (see Fig. 5). This simple spatially extended biochemical model is therefore insufficient to explain sustained cell polarization, as it fails to maintain a stable polarized state.

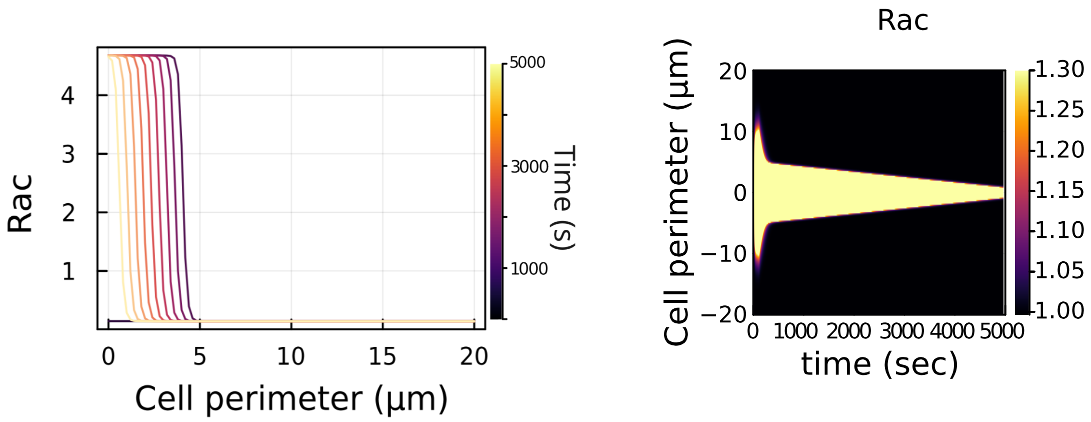

Supplementary Text Figure 5: Absence of steady-state solutions in the presence of diffusion. Left: Successive spatial profiles of membrane-bound Rac concentration $\bar{R}$ over time, showing the decay of polarization. Right: Kymograph of $\bar{R}$, with time on the x-axis and spatial position on the y-axis, illustrating the progressive flattening of the Rac concentration profile.

To overcome this limitation, a class of models known as wave-pinning models has been proposed. These models introduce the key assumption of mass conservation between the active and inactive forms of GTPases, which enables the stabilization of the polarization front. We introduce this framework in the following section.

3 Wave pinning model

We generalize below our spatially extended biochemical model to explicitly account for the reversible switching between active and inactive states of GTPases, while assuming a fixed total amount for each protein. This approach follows the Wave-Pinning (WP) framework introduced in [2].

In this context, bistability for a single diffusive Rho GTPase emerges from the interplay between a nonlinear activation term and the constraint of mass conservation between the active and inactive forms. The governing equations for this system are:

$$\frac{1}{d_R}\frac{\partial \bar{R}}{\partial t} = \bar{\alpha}_0 \frac{1}{1+\bar{\rho}^2}\bar{R}_i + \bar{s}_R - \bar{R} + D_R\nabla^2\bar{R} \tag{18a}$$

$$\frac{1}{d_\rho}\frac{\partial \bar{\rho}}{\partial t} = \bar{\beta}_0 \frac{1}{1+\bar{R}^2}\bar{\rho}_i + \bar{s}_\rho - \bar{\rho} + D_\rho\nabla^2\bar{\rho}. \tag{18b}$$

$$\frac{1}{d_R}\frac{\partial \bar{R}_i(\xi,t)}{\partial t} = -\bar{\alpha}_0 \frac{1}{1+\bar{\rho}^2}\bar{R}_i - \bar{s}_R + \bar{R} + D_{Ri}\nabla^2\bar{R} \tag{18c}$$

$$\frac{1}{d_\rho}\frac{\partial \bar{\rho}_i(\xi,t)}{\partial t} = -\bar{\beta}_0 \frac{1}{1+\bar{R}^2}\bar{\rho}_i - \bar{s}_\rho + \bar{\rho} + D_{\rho i}\nabla^2\bar{\rho}. \tag{18d}$$

where R_i and ρ_i denote the cytoplasmic concentrations of inactive Rac and Rho GTPases at the membrane. Their diffusion coefficients, $D_{\rho i}$ and D_{Ri}, are assumed to be much larger than those of their active membrane-bound counterparts, reflecting the faster diffusion of GTPases in the cytoplasm.

To account for mass conservation, we define the total number of moles of Rac and Rho respectively (active + inactive) as follows

$$\int \Big(R(\xi) + R_i(\xi)\Big) d\xi = N_R, \tag{19}$$

and

$$\int \Big(\rho(\xi) + \rho_i(\xi)\Big) d\xi = N_\rho, \tag{20}$$

Based on the assumption that inactive GTPases diffuse rapidly, we approximate their spatial profiles as uniform. Specifically, we write:

$$R_i(\xi) \simeq \langle R_i \rangle, \tag{21}$$

$$\rho_i(\xi) \simeq \langle \rho_i \rangle, \tag{22}$$

where the angle brackets $\langle \cdot \rangle$ denote the spatial average over the domain. From this approximation, we deduce the following mass conservation relation:

$$\langle R_i \rangle \cdot L = N_R - \int R(\xi)\, d\xi, \tag{23}$$

where L is the total membrane length. An analogous relation holds for the Rho GTPase system:

$$\langle \rho_i \rangle \cdot L = N_\rho - \int \rho(\xi)\, d\xi. \tag{24}$$

This leads to an updated set of equations that incorporate mass conservation explicitly:

$$\frac{1}{d_R}\frac{\partial \bar{R}}{\partial t} = \bar{\alpha}_0 \frac{1}{1+\bar{\rho}^2}\langle \bar{R}_i \rangle \ + \bar{s}_R - \bar{R} + D_R \nabla^2 \bar{R}, \tag{25a}$$

$$\frac{1}{d_\rho}\frac{\partial \bar{\rho}}{\partial t} = \bar{\beta}_0 \frac{1}{1+\bar{R}^2}\langle \bar{\rho}_i \rangle + \bar{s}_\rho - \bar{\rho} + D_\rho \nabla^2 \bar{\rho}, \tag{25b}$$

$$\langle \bar{R}_i \rangle \ = \frac{1}{L}\Big(\bar{N}_R - \int \bar{R}(\xi)\, d\xi\Big), \tag{25c}$$

$$\langle \bar{\rho}_i \rangle = \frac{1}{L}\Big(\bar{N}_\rho - \int \bar{\rho}(\xi)\, d\xi\Big). \tag{25d}$$

3.1 Implementation of the wave-pinning model

The wave-pinning (WP) model demonstrates bistability and the capacity to polarize, as illustrated in Supplementary Text Fig. 6 for a specific set of parameters.

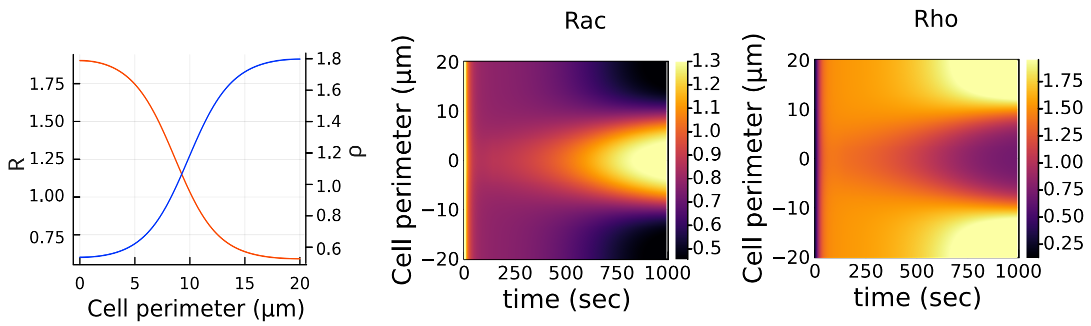

Supplementary Text Figure 6: Wave pinning simulations using $\bar{N}_R = 2$ and $\bar{N}_\rho = 2$ and $\bar{\alpha} = \bar{\beta} = 3.0$. Here we have the final profiles of Rac and Rho (R and ρ) as well as their kymographs over the cell perimeter and time.

However, the bistable region in the extended system becomes highly dependent on the total amount of Rac and Rho, denoted by $\bar{N}_R$ and $\bar{N}_\rho$, in addition to their respective production rates, $\bar{\alpha}_0$ and $\bar{\beta}_0$, as shown in Supplementary Text Fig. 7. For the system to polarize, the total number of moles of each GTPase must be sufficiently large; moreover, the bistable region expands with increasing total mass. Interestingly, each GTPase exhibits a relatively independent bistable parameter region, determined primarily by its own production rate and total mass. This occurs largely independently of the mutual inhibition between Rho and Rac observed in the point-like system, which effectively becomes negligible in this spatially extended context with a wave-pinning mechanism.

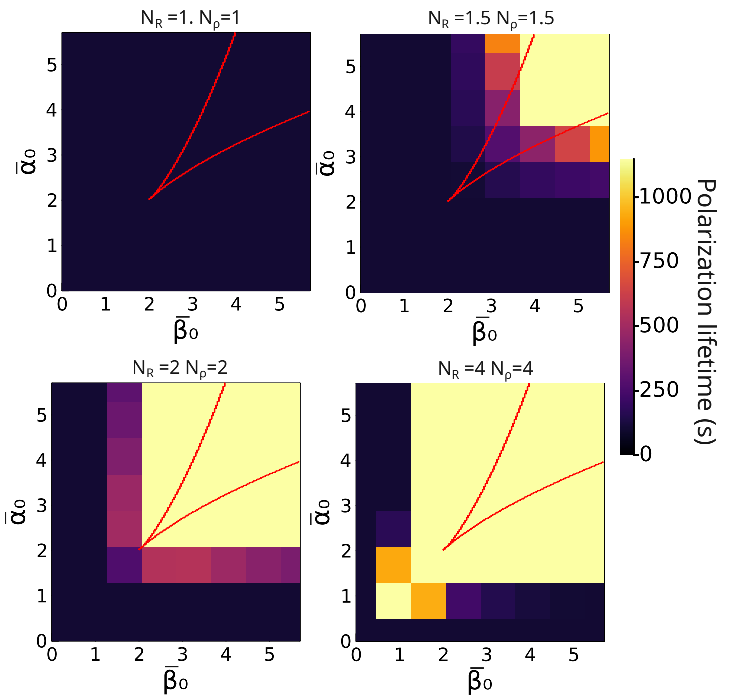

Supplementary Text Figure 7: Heatmap of polarization lifetime in the wave-pinning model as a function of the dimensionless production rates of Rac and Rho, $\bar{\alpha}_0$ and $\bar{\beta}_0$, for four different values of total Rac and Rho levels, N_R and N_ρ. The transition curve for the mutual inhibition model alone is indicated in red.

3.2 Polarization lifetime under external stimulation for the wave-pinning model

The source terms $\bar{s}_R$ and $\bar{s}_\rho$ can be used to simulate optogenetic or chemotactic activation of Rac and Rho within a cell. In Supplementary Text Fig. 8, we examine the persistence of polarization following optogenetic activation under various conditions, while keeping the production rates of Rac and Rho ($\bar{\alpha}_0$ and $\bar{\beta}_0$) fixed.

The results shown in Supplementary Text Fig. 8 highlight key features of Rac–Rho polarization under the WP model. First, polarization is highly sensitive to the total amounts of Rac and Rho (N_R and N_ρ), with polarization failing to occur below a critical threshold. Second, when the WP model permits polarization under external stimulation, it exhibits an effectively binary response to stimuli—either a minimal response or full polarization—with little evidence for intermediate states. Notably, under polarizable conditions, even weak activation can be sufficient to trigger a polarized state, implying a generic hypersensitivity. However, this behavior is not observed experimentally in our system: weak optogenetic activation does not reliably drive polarization under otherwise permissive conditions. This extreme sensitivity may therefore be a limitation for some biological systems, where polarization should require a stimulus of sufficient magnitude to avoid spontaneous activation by stochastic fluctuations, as is typically the case for neutrophils.

3.3 Limitations of wave pinning models for GTPase polarization

The proposed system achieves polarization through a diffusion-driven mechanism coupled with GTPase mass conservation. Former experimental evidence suggested that membrane diffusion alone may be insufficient to drive polarization in cells [15]. While the WP model demonstrates the capacity for spontaneous polarization (which is not observed physiologically in neutrophils in our experimental conditions), this diffusion-based mechanism alone, coupled with the observation of its inherent hypersensitivity to external perturbation, suggests that it may not be physiologically sufficient on its own.

Our experimental observations reveal furthermore that Rac activation is associated with MCA depletion and membrane blebbing, whereas Rho activation correlates with increased membrane tension. These

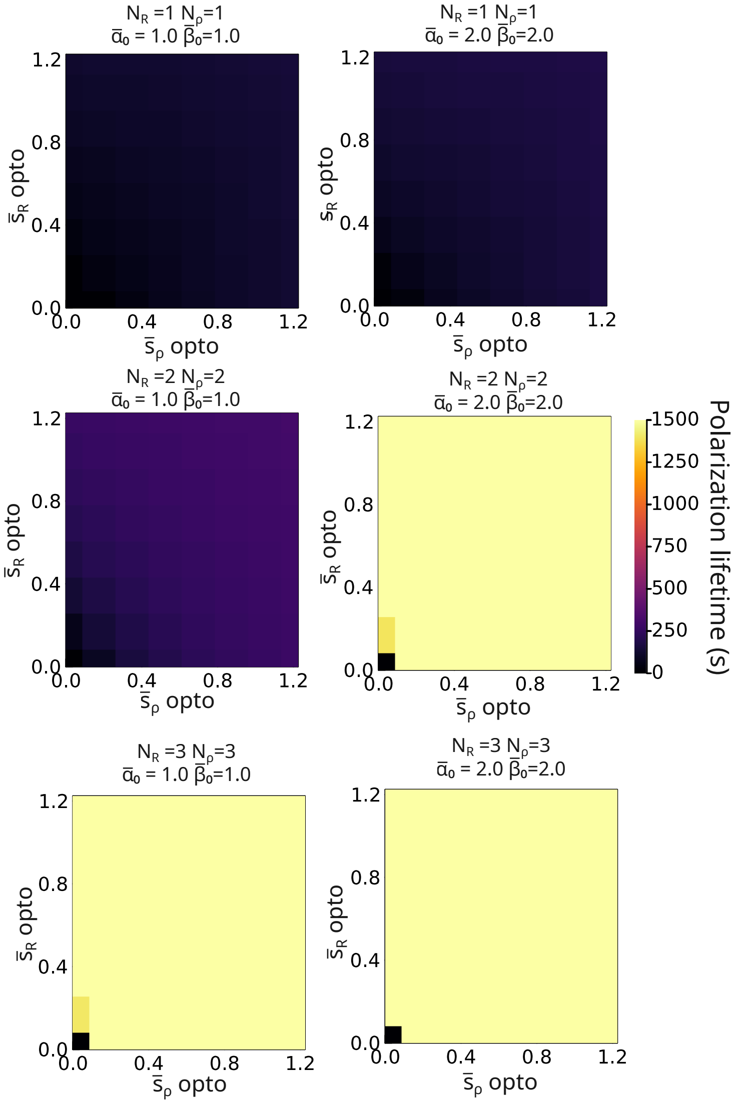

Supplementary Text Figure 8: Heatmap of polarization lifetime in the wave-pinning model as a function of the induced source terms for Rac and Rho, $\bar{s}_R$ and $\bar{s}_\rho$, for three different values of total Rac and Rho levels (N_R and N_ρ). The Rac source is applied at the front of the cell, while the Rho source is applied at the back. Source activation lasts for 100 seconds, after which the polarization lifetime is measured.

new insights motivate the development of a novel integrated model that incorporates mechanical cues alongside the biochemical dynamics of GTPases.

4 Mechanical model

4.1 Hypotheses

To describe the mechanics of the membrane-cortex interaction, we follow a previous composite model [7] that we expand to explicitly account for the inhomogeneous membrane-cortex attachment density (MCA) revealed experimentally (Supp Fig. 5A-D). The membrane mechanics is approximated to a linear elastic response at the macroscopic level, resisting effectively stretching or compressive stress through a membrane tension σ, which may hence be positive or negative. This effective tension is the result of the highly folded structure of the membrane, that results physically from a competition between thermal and active fluctuations, bending rigidity and compressive stress induced by cortical contraction. The cortex is modeled classically as a viscous and contractile shell-like material on the timescales of our experiments $t \simeq 100s$ [10, 11], described by a velocity v and an active contractile stress σ_a. The membrane and cortex interact through MCA proteins, such as ezrin, that are firmly attached to the cortex in their active state but can flow within the membrane plane, that is tangentiallly fluid. The relative movement between the membrane and cortex results therefore in a friction term that is proportional to the relative velocity between the two surfaces and to to the local surface density of MCA proteins. We further account for the binding kinetics of MCA proteins from the membrane to the cortex and therefore distinguish the density of bound proteins ρ_b from unbound density ρ_u. The binding of Ezrin-Radixin-Moesin type of MCA proteins is regulated by their phosphorylation state, they bind to the cortex in the active form and unbinds when it is dephosphorylated [16]. The model components are summarized in the sketch on Supplementary Text Fig. 9.

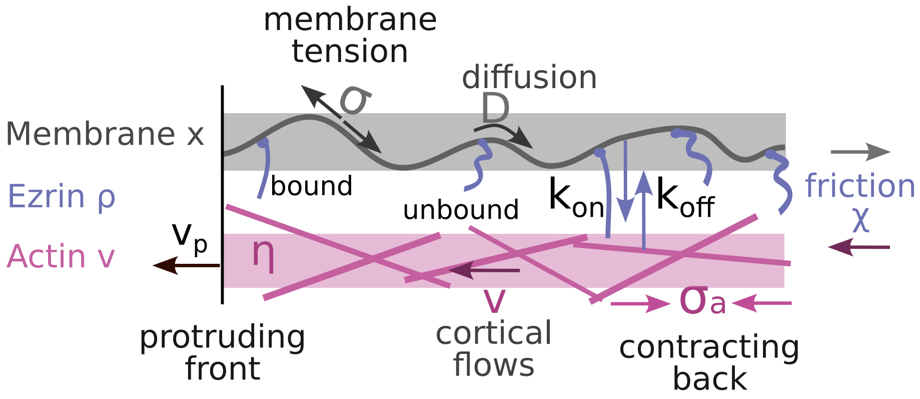

Supplementary Text Figure 9: Representation of the mechanical model.

4.2 One-dimensional formulation

To simplify the description of the cortex-membrane interaction, we start with a one-dimensional description of the membrane as a string of length L. We generalize the model for a 2-dimensional axisymmetric surface later. The composite surface deformation is described in one-dimension with respect to a spatial coordinate ξ.

Let x denote the membrane strain from its relaxed - or reference - state, and $\dot{x} = (\partial x)/(\partial t)$ its Eulerian velocity. We introduce $\xi\,[N.s.m^{-1}]$ the friction per bound MCA linker protein in the membrane and $\sigma_0[N.m^{-1}]$ the two-dimensional membrane elastic modulus associated to its folding/unfolding, and which is homogeneous to a tension. The friction density per unit surface is given by $\rho_b\xi\,[N.s.m^{-3}]$, where $\rho_b\,[m^{-2}]$ is the surface density of bound MCA proteins. Effective tangential force balance in the membrane reads

$$\sigma_0 \nabla^2 x - \chi\rho_b\Big(\dot{x} - v\Big) = 0. \tag{26}$$

Let v denote the local tangential velocity of the cortex. We introduce $\eta\,[N.s.m^{-1}]$ the two-dimensional effective viscosity of the cortex and $\sigma_a\,[N.m^{-1}]$ the two-dimensional active tension in the cortex, regulated

by the local actomyosin contractility. Tangential force balance in the cortex reads

$$\eta\nabla^2 v + \nabla\sigma_a - \chi\rho_b\Big(v - \dot{x}\Big) = 0. \tag{27}$$

We note that the instantaneous local membrane tension may be defined from the local stretch as

$$\sigma = \sigma_0 \nabla x \tag{28}$$

We furthermore introduce the conservation equations for bound and unbound MCA proteins ρ_b and ρ_u at the surface as

$$\frac{\partial \rho_b}{\partial t} + \nabla(v\rho_b) = k_{\text{on}}\rho_u - k_{\text{off}}\rho_b - \lambda_b\rho_b^3, \tag{29a}$$

$$\frac{\partial \rho_u}{\partial t} + \nabla\big(\dot{x}\rho_u\big) = D\nabla^2\rho_u - k_{\text{on}}\rho_u + k_{\text{off}}\rho_b, \tag{29b}$$

where we introduced the binding and unbinding rates $k_{\text{on}}\,[s^{-1}]$ and $k_{\text{off}}\,[s^{-1}]$, a two-dimensional diffusion coefficient $D\,[m.s^{-1}]$ for the unbound MCA linkers within the membrane plane and a non-linear saturation term for the bound protein density λ_b.

$$\sigma_0\frac{\partial^2 x}{\partial \xi^2} - \chi\rho_b(\dot{x} - v) = 0, \tag{30a}$$

$$\eta\frac{\partial^2 v}{\partial \xi^2} + \nabla\sigma_a - \chi\rho_b\Big(v - \dot{x}\Big) = 0, \tag{30b}$$

$$\frac{\partial \rho_b}{\partial t} + \frac{\partial (v\rho_b)}{\partial \xi} = k_{\text{on}}\rho_u - k_{\text{off}}\rho_b - \lambda_b\rho_b^3, \tag{30c}$$

$$\frac{\partial \rho_u}{\partial t} + \frac{\partial (\dot{x}\rho_u)}{\partial \xi} = D\frac{\partial^2 \rho_u}{\partial \xi^2} - k_{\text{on}}\rho_u + k_{\text{off}}\rho_b, \tag{30d}$$

These equations need to be complemented by appropriate boundary and intial conditions. We assume that the protrusive front can be modeled through a Dirichlet boundary conditions as a protrusive velocity v_p for the cortex and a corresponding deformation due to protrusions for the membrane at $\xi = 0$. Since our one-dimensional system shall be seen as a representation of an axisymmetric mmembrane, "unwrapped" into a one-dimensional string, we furthermore impose zero Dirichlet boundary conditions for both the membrane strain and cortex velocity by symmetry at the opposite side of the cell (back) $\xi = L$:

$$v(\xi = 0) = v_p\,, \quad \text{and} \quad v(\xi = L) = 0 \tag{31}$$

$$x(\xi = 0) = \int_0^t v_p(t)\,dt.\,, \quad \text{and} \quad x(\xi = L) = 0 \tag{32}$$

One drawback of the simplified one-dimensional formulation is that the membrane may not relax even when the velocity reaches zero. To address this issue, we introduced an additional slow linear relaxation over time for $x(\xi = 0)$, accounting not only for protrusion retraction but also for other membrane relaxation mechanisms, such as membrane addition via endocytosis or exocytosis.

For MCA surface density, we assume zero flux boundary conditions at membrane edges by symmetry again, which translate into zero Neumann boundary conditions at $\xi = 0$ and $\xi = L$

$$\frac{\partial \rho_b}{\partial \xi}\bigg|_{0,L} = 0 \quad \text{and} \quad \frac{\partial \rho_u}{\partial \xi}\bigg|_{0,L} = 0. \tag{33}$$

The initial values of bound and unbound densities are homogeneous and set such that their sum equals a conserved total number of MCA proteins, defined as $M_\rho = \int(\rho_b + \rho_u)d\xi$, which allows us to define a characteristic mean MCA density in one dimension

$$\rho_0 \equiv \frac{M_\rho}{L} \tag{34}$$

Unless specified, all other variables are set initially to their homogeneous basal or stationary values.

Although the parameters in the one-dimensional formulation of the model have different dimensions than their two-dimensional counterparts, the dimensionless parameters we will define further remain unchanged, and their numerical values can therefore be directly inferred from experimental measurements.

5 Mechanochemical model

5.1 Mechanochemical couplings

The mechanochemical model combines the local biochemical model for Rho and Rac described in Section 2 and the composite membrane-cortex mechanics described in Section 4. The control of Rho and Rac on cortical contractility and protrusion activity respectively are well described in the literature [17]. The feedbacks of mechanics on biochemical regulation are in contrary formulated based on experimental results from this manuscript.

Cortical tension
The dependence of the contractile cortical tension on Rho concentration in the membrane is assumed to be linear[18]:

$$\sigma_a = \sigma_a^0 \, \frac{b}{k_R}, \tag{35}$$

where σ_a^0 is a basal contractile tension in the cortex.

Protrusive velocity
For the protrusive activity, we assume that the protrusion velocity is activated with Rac and we introduce a switch mechanism where protrusion occurs above a given Rac concentration threshold R_{th}, while also accounting for the non-linear resistance exerted by membrane tension to protrusion σ [19]

$$v_p \simeq \frac{v_0}{(1+\sigma^2/\sigma_0^2)} \text{th}\big(R - R_{\text{th}}\big), \tag{36}$$

$$\text{where} \qquad \text{th}\big(R - R_{\text{th}}\big) = \frac{1}{2}\Big[\tanh\Big(\frac{R - R_{\text{th}}}{k_R}\Big) + 1\Big].$$

Rac activation
Based on experimental results on Fig. 4, we furthermore assume that the Rac activation rate depends on the bound MCA concentration ρ_b in a switch like manner, where it is increased below a threshold value ρ_{th}. We do not model explicitly stochastic blebbing and the competition for PIP2 binding sites for simplicity. The spatially-resolved equation for Rac dynamics is modified accordingly as follows

$$\frac{\partial R}{\partial t} = \Big(\alpha_0 + \alpha \, \text{th}(\rho_b - \rho_{\text{th}})\Big) \frac{k_R^2}{k_R^2 + \rho^2} - d_R R + D_R \nabla^2 R, \tag{37}$$

$$\text{where} \qquad \text{th}(\rho_b - \rho_{\text{th}}) = \frac{1}{2}\Big[1 - \tanh\Big(\frac{\rho_b - \rho_{\text{th}}}{\rho_0}\Big)\Big],$$

where we introduced a membrane diffusion coefficient D_R for Rac, a basal value ρ_0 for the MCA surface density that will be defined later, and an additional activation rate α for the dependence on MCA surface density.

Rho activation
Based on experimental results on Fig. 2E-L, we finally assume that the Rho activation rate depends on the membrane tension σ in a switch like manner, where it is increased above a threshold value σ_{th}. The spatially-resolved equation for Rho dynamics is modified accordingly as follows

$$\frac{\partial \rho}{\partial t} = \Big(\beta_0 + \beta \, \text{th}(\sigma - \sigma_{th})\Big) \frac{k_\rho^2}{k_\rho^2 + R^2} - d_\rho \rho + D_\rho \nabla^2 \rho \tag{38}$$

$$\text{where} \qquad \text{th}(\sigma - \sigma_{\text{th}}) = \frac{1}{2}\Big[\tanh\Big(\frac{\sigma - \sigma_{\text{th}}}{\sigma_0}\Big) + 1\Big],$$

where we introduced a membrane diffusion coefficient D_ρ for Rho, and an additional activation rate β for the dependence on membrane tension.

5.2 Coupled dynamic equations

The full one-dimensional mechanochemical model has six dynamical variables R, ρ, v, x, ρ_b and ρ_u, the dynamics of which is governed by six partial differential equations

$$\frac{\partial R}{\partial t} = \Big(\alpha_0 + \alpha\, \mathrm{th}(\rho_b - \rho_{\mathrm{th}})\Big)\frac{k_R^2}{k_R^2+\rho^2} - d_R R + D_R \nabla^2 R, \tag{39a}$$

$$\frac{\partial \rho}{\partial t} = \Big(\beta_0 + \beta\, \mathrm{th}(\sigma - \sigma_{th})\Big)\frac{k_\rho^2}{k_\rho^2+R^2} - d_\rho \rho + D_\rho \nabla^2 \rho, \tag{39b}$$

$$\sigma_0 \nabla^2 x - \chi\, \rho_b \Big(\dot{x} - v\Big) = 0, \tag{39c}$$

$$\eta \nabla^2 v + \sigma_a^0 \nabla \left(\frac{\rho}{k_R}\right) - \chi\, \rho_b \Big(v - \dot{x}\Big) = 0, \tag{39d}$$

$$\frac{\partial \rho_b}{\partial t} + \nabla(v \cdot \rho_b) = k_{\mathrm{on}}\rho_u - k_{\mathrm{off}}\rho_b, \tag{39e}$$

$$\frac{\partial \rho_u}{\partial t} + \nabla\big(\dot{x} \cdot \rho_u\big) = -k_{\mathrm{on}}\rho_u + k_{\mathrm{off}}\rho_b + D\nabla^2 \rho_u, \tag{39f}$$

To these equations, one has to add the boundary conditions, which have been made explicit in one dimension in (32), (31), (33). Additional zero-flux boundary conditions for Rac and Rho surface density R and ρ can be expressed identically to those for ρ_b and ρ_u (33) as Neumann boundary conditions. Finally, the explicit coupling of the protrusion velocity with other variables is defined in (36).

5.3 Non-dimensionalization

We non-dimensionalize the previous equations as follows:

We set a basal contractile tension in the cortex σ_a^0, which allows us to non-dimensionalize the membrane tension and to define a characteristic timescale associated to the active-viscous cortex relaxation that will serve to non-dimensionalize all other times

$$\bar{\sigma}_0 = \frac{\sigma_0}{\sigma_a^0}, \quad \tau_a \equiv \frac{\eta}{\sigma_a^0}, \quad \bar{t} = \frac{t}{\tau_a}. \tag{40}$$

We define a hydrodynamic length λ measuring the spatial extent of cortical viscous flows slowed down by friction and we non-dimensionalize all lengths by a characteristic size L of the cell

$$\lambda \equiv \sqrt{\frac{\eta}{\chi\,\rho_0}}, \quad \bar{\lambda} = \frac{\lambda}{L}, \quad \bar{\xi} = \frac{\xi}{L}, \quad \bar{x} = \frac{x}{L}, \quad \bar{v} = v\frac{\tau_a}{L}. \tag{41}$$

MCA surface densities are non-dimensionalized using the mean density ρ_0 defined in (34)

$$\rho_b = \frac{\rho_b}{\rho_0}, \quad \bar{\rho}_u = \frac{\rho_u}{\rho_0}, \quad \bar{\lambda}_b = \lambda_b \frac{\rho^2}{k_{\mathrm{off}}} \tag{42}$$

We further define a dimensionless reaction rate constant as ratio of binding and unbinding rates and an effective Peclet number

$$K \equiv \frac{k_{\mathrm{on}}}{k_{\mathrm{off}}}, \quad \mathcal{P}e \equiv \frac{L}{\tau_a}\frac{L}{D} = \frac{v_a L}{D}, \tag{43}$$

where $v_a \equiv L/\tau_a$ is a basal advection velocity by cortical flows.

For Rac and Rho surface densities, we choose k_R and k_ρ as characteristic concentrations and non-dimensionalize other parameters using previously defined time and lengthscales

$$\bar{R} = \frac{a}{k_\rho}, \quad \bar{\rho} = \frac{b}{k_R}, \quad \bar{\alpha}_{(0)} = \frac{\alpha_{(0)}}{k_\rho\, d_R}, \quad \bar{\beta}_{(0)} = \frac{\beta_{(0)}}{k_R\, d_\rho}, \quad \bar{d}_{R,\rho} = d_{R,\rho}\tau_a, \quad \bar{D}_{R,\rho} = D_{R,\rho}\frac{\tau_a}{L^2}. \tag{44}$$

The resulting dimensionless set of equations become

$$\frac{1}{\bar{d}_R}\frac{\partial \bar{R}}{\partial \bar{t}} - \bar{D}_R \bar{\nabla}^2 R = \Big(\bar{\alpha}_0 + \bar{\alpha}\, \mathrm{th}(\bar{\rho}_b - \bar{\rho}_{th})\Big)\frac{1}{1+\bar{\rho}^2} - \bar{R} \tag{45a}$$

$$\frac{1}{\bar{d}_\rho}\frac{\partial\bar{\rho}}{\partial\bar{t}} - \bar{D}_\rho\bar{\nabla}^2\rho = \left(\bar{\beta}_0 + \bar{\beta}\,\mathrm{th}(\bar{\sigma}-\bar{\sigma}_{th})\right)\frac{1}{1+\bar{R}^2} - \bar{\rho} \tag{45b}$$

$$\bar{\sigma}_0\nabla^2\bar{x} - \frac{1}{\bar{\lambda}^2}\bar{\rho}_b\left(\dot{\bar{x}} - \bar{v}\right) = 0 \tag{45c}$$

$$\bar{\nabla}^2\bar{v} + \bar{\nabla}\bar{\rho} - \frac{1}{\bar{\lambda}^2}\bar{\rho}_b\left(\bar{v} - \dot{\bar{x}}\right) = 0 \tag{45d}$$

$$\frac{1}{\bar{k}_{\mathrm{off}}}\left(\frac{\partial\bar{\rho}_b}{\partial\bar{t}} + \nabla(\bar{v}\cdot\bar{\rho}_b)\right) = K\bar{\rho}_u - \bar{\rho}_b - \bar{\lambda}_b\,\bar{\rho}_b^3 \tag{45e}$$

$$\frac{1}{\bar{k}_{\mathrm{off}}}\left(\frac{\partial\bar{\rho}_u}{\partial\bar{t}} + \nabla(\dot{\bar{x}}\cdot\bar{\rho}_u) - \frac{1}{\mathcal{P}e}\nabla^2\bar{\rho}_u\right) = -K\bar{\rho}_u + \bar{\rho}_b \tag{45f}$$

5.4 Temporal persistence of polarization under optogenetic stimulation

In this section, we investigate the temporal persistence of cell polarization when optogenetic forcing of Rac or Rho is applied, while keeping the basal production rates $\bar{\alpha}_0$ (for Rac) and $\bar{\beta}_0$ (for Rho) fixed.

In the main text (Fig. 7C), we identify three distinct regions in the ($\bar{\alpha}_0$, $\bar{\beta}_0$) parameter space:

1. A **biochemically bistable region**, where the system supports two stable steady states based on biochemical interactions alone.
2. A **mechanochemically polarizable region**, where persistent polarization arises due to feedback from mechanical processes, even though the biochemical system by itself is not bistable.
3. A **non-polarizable region**, where polarization cannot be sustained under any input.

We illustrate the influence of optogenetic Rho/Rac forcing across these three regions in Supplementary Text Fig. 10, from left to right:

- **Non-polarizable region:** In this regime, no level of optogenetic input is sufficient to maintain a steady polarized state. This scenario is representative of neutrophil-like cells, which exhibit only transient polarization and ultimately return to an unpolarized state.
- **Mechanochemically polarizable region:** Starting from an unpolarized state, a sufficiently strong input—especially when both Rac and Rho are optogenetically activated—can drive the system toward persistent polarization. Notably, the response is asymmetric with respect to Rac and Rho inputs ($\bar{s}_R$, $\bar{s}_\rho$), reflecting differences in how Rac and Rho interact with the mechanical feedback. This asymmetry arises from differences in mechanical coupling strength, activation thresholds, and the nonlinearities in the biochemical-mechanical interaction. Importantly, the degree of asymmetry is sensitive to parameter changes within this coupling.
- **Biochemically bistable region:** Here, the system becomes ultrasensitive to small perturbations in Rac or Rho levels. Even minimal optogenetic stimulation can induce spontaneous polarization due to the inherent bistability of the biochemical network.

A key distinction between the mechanochemical model and a purely biochemical model (as depicted in Supplementary Text Fig. 4) lies in their dependence on initial conditions. In the mechanochemical model, polarization occurs robustly regardless of the initial distribution of active GTPases. Once the system enters the bistable region, mechanical feedback ensures symmetry breaking and sustained polarization, whether the activated GTPase initially resides in a high or low concentration state.

In contrast, in a purely biochemical system, the outcome strongly depends on initial conditions. If optogenetic activation targets only the GTPase already in a high concentration regime, the system may not respond or evolve toward a new polarized state, underscoring the importance of mechanochemical feedback for robust symmetry breaking.

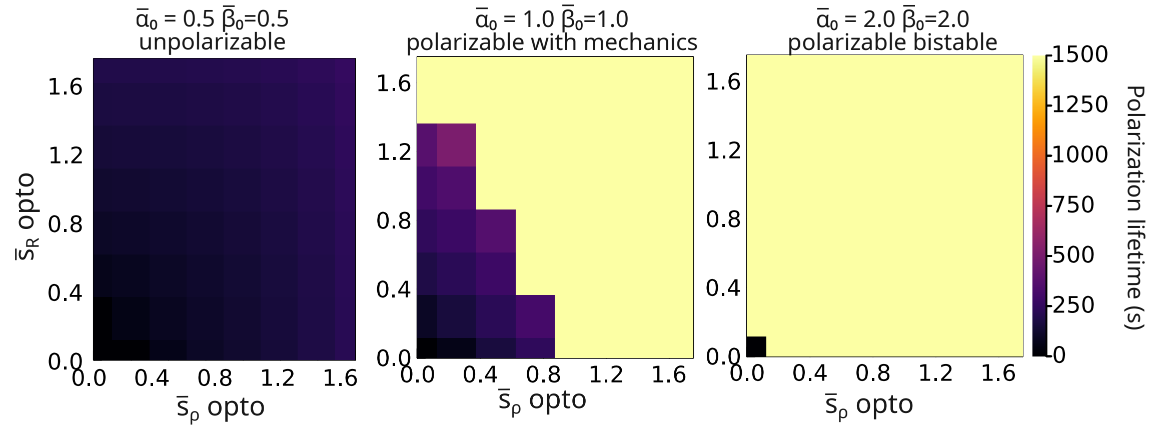

Supplementary Text Figure 10: **Temporal persistence of cell polarization following optogenetic stimulation of Rac and Rho.** The heatmap shows how long the cell remains polarized after optogenetic exposure of a given intensity, with inputs s_R (Rac) and s_ρ (Rho). Simulations are based on the mechanochemical model without mass conservation of GTPases. Optogenetic stimulation is applied for 100 seconds at each end of the cell, after which the input is removed. The total simulation time is 1500 seconds; polarization durations of 1500 seconds or more are shown in yellow, as the colormap saturates at this upper limit.

6 Axisymmetric Two-Dimensional Model

6.1 Derivation of the Mechanical Equations for a Spherical Membrane

To model an axisymmetric membrane, the governing equations must account for surface curvature. Following a mechanical framework adapted from [20], we introduce the corresponding modifications to the membrane equations. In this section, we derive the membrane stress tensor needed for the in-plane tension balance. Owing to the spherical geometry, we use spherical coordinates (θ, φ) for the derivation.

6.1.1 Preliminary Geometrical Parametrization

We define the membrane material points by the position vector $\boldsymbol{X}_0$ in the natural (stress-free) configuration and by $\boldsymbol{x}(\boldsymbol{X}_0, t)$ in the current configuration. The deformation from the reference configuration is characterized by the deformation gradient tensor $\underline{\underline{F}} = \partial \boldsymbol{x} / \partial \boldsymbol{X}_0$. The metric change induced by deformation is described by the right Cauchy–Green tensor $\underline{\underline{C}} = \underline{\underline{F}}^T \cdot \underline{\underline{F}}$.

In the reference configuration, the membrane lies on the sphere $\boldsymbol{X}_0 = R_0 \boldsymbol{e}_r(\theta, \varphi)$. We first apply an isotropic inflation to an intermediate (prestretched) configuration,

$$\boldsymbol{X} = R\, \boldsymbol{e}_r = \xi\, \boldsymbol{X}_0, \qquad \xi \equiv R/R_0, \tag{46}$$

where ξ denotes the isotropic biaxial stretch (prestretch). The current membrane shape is then written as

$$\boldsymbol{x}(\boldsymbol{X}_0, t) = \boldsymbol{X} + \boldsymbol{u}(\boldsymbol{X}_0, t) = \xi \boldsymbol{X}_0 + \boldsymbol{u}(\boldsymbol{X}_0, t)\,, \tag{47}$$

where $\boldsymbol{u}(\theta, \varphi, t) = (u_r, u_\theta, u_\varphi)$ is the displacement from the intermediate configuration.

The deformation gradient $\underline{\underline{F}}$ maps the reference tangent plane to $\mathbb{R}^3$ and can be represented as a 3×2 matrix:

$$\underline{\underline{F}} = \frac{\partial \boldsymbol{x}}{\partial \boldsymbol{X}_0} = \frac{\partial \boldsymbol{x}}{\partial \boldsymbol{X}} \cdot \frac{\partial \boldsymbol{X}}{\partial \boldsymbol{X}_0} = \xi \left(\mathbb{1}_{3\times2} + \underline{\underline{\boldsymbol{\nabla} u}} \right)\,, \tag{48}$$

where $\mathbb{1}_{3\times2}$ denotes the canonical embedding of the surface tangent plane into $\mathbb{R}^3$, and $\underline{\underline{\boldsymbol{\nabla} u}}$ is the (surface) displacement gradient taken with respect to the intermediate configuration. The right Cauchy–Green tensor becomes

$$\underline{\underline{C}} = \xi^2 \left[\mathbb{1}_{2\times2} + \underline{\underline{\boldsymbol{\nabla}_{\mathrm{s}} \boldsymbol{u}}} + \underline{\underline{\boldsymbol{\nabla}_{\mathrm{s}} \boldsymbol{u}}}^T + \underline{\underline{\boldsymbol{\nabla} \boldsymbol{u}}}^T \cdot \underline{\underline{\boldsymbol{\nabla} \boldsymbol{u}}} \right] \equiv \xi^2 \left[\mathbb{1}_{2\times2} + 2 \underline{\underline{e}} \right]\,, \tag{49}$$

which defines the incremental finite strain tensor

$$\underline{\underline{e}} = \frac{1}{2} \left(\underline{\underline{\boldsymbol{\nabla}_{\mathrm{s}} u}} + \underline{\underline{\boldsymbol{\nabla}_{\mathrm{s}} u}}^T \right) + \frac{1}{2} \underline{\underline{\boldsymbol{\nabla} u}}^T \cdot \underline{\underline{\boldsymbol{\nabla} u}}\,. \tag{50}$$

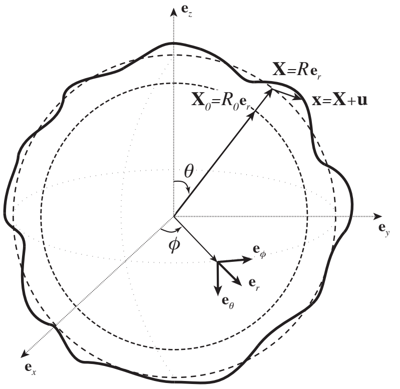

Supplementary Text Figure 11: The fluctuating elastic membrane (bold line) is described by the position vector $\boldsymbol{x} = \boldsymbol{X} + \boldsymbol{u}$, where the displacement $\boldsymbol{u}$ from the intermediate configuration is expressed in the spherical basis $(\boldsymbol{e}_r, \boldsymbol{e}_\theta, \boldsymbol{e}_\varphi)$. In the reference configuration, the membrane lies on the sphere $\boldsymbol{X}_0 = R_0\boldsymbol{e}_r$, and in the intermediate prestretched configuration it is $\boldsymbol{X} = R\boldsymbol{e}_r = \xi R_0 \boldsymbol{e}_r$.

Here, $\boldsymbol{\nabla}_\mathrm{s}$ denotes the surface gradient operator on the prestretched (intermediate) sphere.

6.1.2 Incremental Linear Elastic Energy Density

For a 2D isotropic elastic material, the energy density $\mathcal{E}$ depends on the invariants $I_1 = \mathrm{tr}\,(\underline{\underline{C}})$ and $I_2 = \det(\underline{\underline{C}})$. Expressed in terms of the prestretch ξ and the incremental strain $\underline{\underline{e}}$, these invariants read

$$I_1 = 2\xi^2\Big[1 + \mathrm{tr}\,(\underline{\underline{e}})\Big]\,, \tag{51a}$$

$$I_2 = \xi^4\Big[1 + 2\mathrm{tr}\,(\underline{\underline{e}}) + 2\mathrm{tr}^2(\underline{\underline{e}}) - 2\mathrm{tr}\,(\underline{\underline{e}}^2)\Big]\,. \tag{51b}$$

Expanding $\mathcal{E}(I_1, I_2)$ to second order in $\underline{\underline{e}}$ yields a Hookean law with prestress [21]:

$$\mathcal{E} = \mathcal{E}_\mathrm{i} + S\,\mathrm{tr}\,(\underline{\underline{e}}) + \frac{\Lambda}{2}\,\mathrm{tr}^2(\underline{\underline{e}}) + M\,\mathrm{tr}\,(\underline{\underline{e}}^2)\,, \tag{52}$$

where the coefficients S, Λ, and M are determined by derivatives of the constitutive law evaluated in the prestretched state.

6.1.3 Homogenization of an Hexagonal Spring Network

To bridge the 2D and 1D descriptions, we homogenize an isotropic hexagonal network of filaments with natural length ℓ_0. Following the observation by Kantor [22] that sixfold symmetry implies isotropic elasticity, the discrete elastic energy is averaged over filament directions α. Using the filament stretch $\lambda = \xi(1 + 2e_{tt})^{1/2}$, the resulting (local) energy density takes the form

$$\mathcal{E} = \mathcal{E}_\mathrm{i} + S\,\mathrm{tr}\,(\underline{\underline{e}}) + \left(\frac{\Lambda}{2}\mathrm{tr}^2(\underline{\underline{e}}) + M\,\mathrm{tr}\,(\underline{\underline{e}}^2)\right)\,, \tag{53}$$

and the surface element in the intermediate configuration satisfies $dA = \xi^2 dA_0$. For a hexagonal network of linear springs (with $e(\lambda) = \frac{k}{2}\ell_0^2(\lambda - 1)^2$), the effective moduli are

$$\mathcal{E}_\mathrm{i}(\xi) = k\sqrt{3}\big(1 - 1/\xi\big)^2\,, \tag{54a}$$

$$S(\xi) = k\sqrt{3}\,\big(1 - 1/\xi\big)\,, \tag{54b}$$

$$\Lambda(\xi) = M(\xi) = \frac{\sqrt{3}}{4}\frac{k}{\xi}\,. \tag{54c}$$

In the reference state ($\xi = 1$), these satisfy the Lamé relations $\lambda_0 = \mu_0 = \frac{\sqrt{3}}{4}k$.

6.1.4 Energy in Axisymmetric Geometry with

In spherical coordinates, the (surface) displacement gradient involves derivatives with respect to (θ, φ). Under axisymmetry we assume $\partial_\varphi(\cdot) = 0$, $u_\varphi = 0$, and, for simplicity, we neglect radial deformation in this model ($u_r = 0$), retaining only the tangential displacement $u_\theta(\theta, t)$. The displacement gradient then simplifies to

$$\underline{\underline{\nabla u}} = \frac{1}{R}\begin{pmatrix} -u_\theta & 0 \\ \frac{\partial u_\theta}{\partial \theta} & 0 \\ 0 & \cot\theta\, u_\theta \end{pmatrix} . \tag{55}$$

To second order in $\underline{\underline{\nabla u}}$, the incremental strain $\underline{\underline{e}}$ and the relevant traces are

$$\underline{\underline{e}} = \frac{1}{R}\begin{pmatrix} u_{\theta\theta} & 0 \\ 0 & u_{\varphi\varphi} \end{pmatrix} + \frac{1}{2R^2}\begin{pmatrix} u_{\theta\theta}^2 & 0 \\ 0 & u_{\varphi\varphi}^2 \end{pmatrix} , \tag{56a}$$

$$\operatorname{tr}(\underline{\underline{e}}) = \frac{1}{R}\left[(u_{\theta\theta} + u_{\varphi\varphi}) + \frac{1}{2R}\left(u_{\theta\theta}^2 + u_{\varphi\varphi}^2\right)\right] , \tag{56b}$$

$$\operatorname{tr}^2(\underline{\underline{e}}) = \frac{1}{R^2}\left(u_{\theta\theta} + u_{\varphi\varphi}\right)^2 , \qquad \operatorname{tr}(\underline{\underline{e}}^2) = \frac{1}{R^2}\left(u_{\theta\theta}^2 + u_{\varphi\varphi}^2\right) , \tag{56c}$$

where $u_{\theta\theta} = \partial_\theta u_\theta$ and $u_{\varphi\varphi} = u_\theta \cot\theta$. These relations define the energy density $\mathcal{E}(\boldsymbol{u})$ as a function of the membrane displacement.

6.1.5 Membrane tension balance

From the energy density (53), we obtain the two-dimensional membrane stress tensor (valid for small deformations) as

$$\underline{\underline{N}}^{\mathrm{mbr}} = \frac{\partial \mathcal{E}}{\partial \underline{\underline{e}}} = S\,\mathbb{1}_s + \Lambda \operatorname{tr}(\underline{\underline{e}})\,\mathbb{1}_s + 2M\,\underline{\underline{e}} , \tag{57}$$

where $\mathbb{1}_s$ denotes the in-surface identity tensor.

The in-plane membrane tension along a unit tangent direction τ is computed as

$$\sigma^{\mathrm{mbr}} = \tau \cdot \left(\underline{\underline{N}}^{\mathrm{mbr}} \cdot \tau\right) . \tag{58}$$

Balancing the in-plane stress divergence with membrane–cortex friction yields the membrane tension balance equation

$$\boldsymbol{\nabla}_s \cdot \underline{\underline{N}}^{\mathrm{mbr}} = \rho_b \chi \left(\dot{\mathbf{u}} - \mathbf{v}\right) , \tag{59}$$

where $\dot{\mathbf{u}}$ denotes the time derivative of the membrane displacement. This equation replaces (39c).

6.2 Governing Equations for the Integrated 2D Mechanochemical model

6.2.1 Two-dimensional cortical mechanics governing equations

Following [10, 11, 23], we model the cortex as a two-dimensional viscous and contractile layer. It is characterized by an in-plane velocity field $\boldsymbol{v}$, a 2D surface viscosity η, and a tangentially isotropic active (contractile) tension σ_a. Under the simplifying assumption adopted here of zero radial deformation, the cortical in-plane stress tensor is written as

$$\underline{\underline{N}}^{\mathrm{cor}} = \eta\left(\underline{\underline{\nabla_s \boldsymbol{v}}} + \underline{\underline{\nabla_s \boldsymbol{v}}}^T\right) + \sigma_a \mathbb{1}_s . \tag{60}$$

Balancing the in-plane stress divergence with membrane–cortex friction yields the cortical tension balance equation

$$\boldsymbol{\nabla}_s \cdot \underline{\underline{N}}^{\mathrm{cor}} = \rho_b\,\chi \left(\mathbf{v} - \dot{\mathbf{u}}\right) , \tag{61}$$

where $\dot{\mathbf{u}}$ denotes the time derivative of the membrane displacement. This equation replaces (39d).

6.2.2 Governing equations for biochemical species

We now generalize the 1D reaction–diffusion–advection system for the biochemical species to the present 2D axisymmetric setting on a spherical surface. The regulatory species Rac (R) and rho (ρ), as well as the bound and unbound MCA linker densities ρ_b and ρ_u, are modeled as scalar surface fields defined on the (prestretched) membrane/cortex spherical surface. Because we assume zero radial deformation ($u_r = 0$), material transport occurs purely in the tangent plane: the cortical flow $\boldsymbol{v}$ and the membrane velocity $\dot{\mathbf{u}}$ are tangential vector fields. Spatial spreading is captured by surface diffusion through the Laplace–Beltrami operator Δ_s, and advective transport is written in conservative form using the surface divergence $\boldsymbol{\nabla}_s \cdot (\cdot)$. This yields the following governing equations, replacing respectively equations (39a), (39b), (39e), and (39f):

$$\frac{\partial R}{\partial t} = \Big(\alpha_0 + \alpha\,\mathrm{th}(\rho_b - \rho_{\mathrm{th}})\Big)\frac{k_R^2}{k_R^2 + \rho^2} - d_R R + D_R\,\Delta_s R, \tag{62}$$

$$\frac{\partial \rho}{\partial t} = \Big(\beta_0 + \beta\,\mathrm{th}(\sigma - \sigma_{\mathrm{th}})\Big)\frac{k_\rho^2}{k_\rho^2 + R^2} - d_\rho \rho + D_\rho\,\Delta_s \rho, \tag{63}$$

$$\frac{\partial \rho_b}{\partial t} + \boldsymbol{\nabla}_s\cdot(\rho_b\,\boldsymbol{v}) = k_{\mathrm{on}}\rho_u - k_{\mathrm{off}}\rho_b, \tag{64}$$

$$\frac{\partial \rho_u}{\partial t} + \boldsymbol{\nabla}_s\cdot(\rho_u\,\dot{\mathbf{u}}) = -k_{\mathrm{on}}\rho_u + k_{\mathrm{off}}\rho_b + D\,\Delta_s \rho_u. \tag{65}$$

Remarks:
(i) The operators $\boldsymbol{\nabla}_s$ and $\Delta_s = \boldsymbol{\nabla}_s \cdot \boldsymbol{\nabla}_s$ are defined on the prestretched spherical surface (intermediate configuration). Under axisymmetry, all fields are independent of φ and only depend on θ and t, but we keep the intrinsic surface form for compactness and to facilitate extensions beyond axisymmetry.
(ii) The frictional exchange term in the activation kinetics or Rac (R) is controlled by ρ_b, the local density of bound linkers, which also appears in the mechanical membrane–cortex coupling.
(iii) The mechanochemical feedback in the production of ρ involves a scalar tension measure σ. In the 2D framework, σ must be defined from the in-plane stress. A possible choice is the in-plane tangent membrane tension

$$\sigma \equiv \tau \cdot \left(\underline{\underline{N}}^{\mathrm{mbr}} \cdot \tau\right) , \tag{66}$$

although other scalar measures (e.g. isotropic membrane tension, or the mean of principal tensions) can be used depending on the intended biological interpretation.

7 Numerical Implementation

The coupled mechanical and biochemical equations are nondimensionalized as in the one-dimensional model. For readability, we however present the implemented equations in their dimensionalized version below.

7.1 Computational domain, geometry, and surface measure

We consider a spherical cell of radius R and formulate the problem on an axisymmetric spherical surface parameterized by the polar angle θ. On a full sphere, regularity/continuity requirements at the poles preclude imposing non-zero tangential Dirichlet boundary conditions at $\theta = 0$, which is problematic on the protruding side where the tangential cortical velocity must be prescribed. Two strategies are then possible: (1) explicitly model the protrusion as a geometrical surface deformation (at the cost of substantial mathematical and computational complexity), or (2) truncate the protruding region and impose Dirichlet boundary conditions at the resulting interface. In the following, we adopt the latter approach.

Concretely, we remove a spherical cap around the protruding pole and solve on the truncated interval

$$\theta \in [\theta_{\min}, \pi], \qquad \theta_{\min} = 25.8^\circ, \tag{67}$$

which defines the computational surface $\Gamma \subset \mathbb{S}^2$. The boundary $\partial\Gamma$ corresponds to the circular cut at $\theta = \theta_{\min}$, where protrusive boundary conditions can be applied.

In axisymmetry, surface integrals reduce to one-dimensional integrals with the surface measure

$$dA = R^2 \sin\theta \, d\theta, \tag{68}$$

where the azimuthal integration is omitted (equivalently, all surface integrals are understood per unit azimuthal angle).

The implementation is performed in *Julia* using the finite-element library *Gridap* [24, 25]. The numerical framework follows the unfitted strategy described in [23], adapted here to the present mechanochemical system. The code is available at GitHub.

7.2 Weak formulation

The weak form is obtained from the strong form by multiplying each equation by an admissible test function and integrating over Γ. We use $\boldsymbol{\nabla}_s$ for the surface gradient and $\Delta_s = \boldsymbol{\nabla}_s \cdot \boldsymbol{\nabla}_s$ for the Laplace–Beltrami operator. The unknowns are two tangential vector fields, the membrane displacement $\mathbf{u}$ and the cortical velocity $\mathbf{v}$ (with $u_r = 0$), and scalar surface fields R, ρ, ρ_b and ρ_u(biochemical species and MCA linker densities).

7.2.1 Membrane mechanics

Let $\mathbf{w}$ be a tangential vector test function respecting the same boundary conditions as the membrane displacement $\boldsymbol{u}$. The weak form of the membrane tension balance (59) reads

$$\int_\Gamma \Big[\Lambda \,\mathrm{tr}\big(\underline{\underline{e}}(\mathbf{u})\big)\,\mathrm{tr}\big(\underline{\underline{e}}_s(\mathbf{w})\big) + M\,\underline{\underline{e}}(\mathbf{u}) : \underline{\underline{e}}_s(\mathbf{w})\Big]\,dA = \int_\Gamma \chi\,\rho_b\,\Big(\mathbf{w}\cdot\dot{\mathbf{u}} - \mathbf{w}\cdot\mathbf{v}\Big)\,dA, \tag{69}$$

where $\underline{\underline{e}}_s(\mathbf{w}) = \frac{1}{2}\left(\underline{\underline{\nabla_s \mathbf{w}}} + \underline{\underline{\nabla_s \mathbf{w}}}^T\right) + \frac{1}{2}\underline{\underline{\nabla_s \mathbf{w}}}^T \cdot \underline{\underline{\nabla_s \mathbf{w}}}$ is the linear surface strain in the test function. This form results from integration by parts (placing the test function inside the surface strain operator); see [23] for details. The constant prestress term $S\,\mathbb{1}_s$ does not contribute to $\boldsymbol{\nabla}_s \cdot \underline{\underline{N}}^{\mathrm{mbr}}$ and therefore drops out of the weak form.

7.2.2 Cortical mechanics

The cortical stress is $\underline{\underline{N}}^{\mathrm{cor}} = \eta\left(\underline{\underline{\nabla_s \boldsymbol{v}}} + \underline{\underline{\nabla_s \boldsymbol{v}}}^T\right) + \sigma_a \mathbb{1}_s$, (60) and the cortical tension balance is (61). Let $\mathbf{w}$ be a tangential vector test function with same boundary conditions as the velocity $\boldsymbol{v}$. Multiplying by $\mathbf{w}$, integrating over Γ, and integrating by parts the viscous term yields

$$\int_\Gamma 2\eta\,\underline{\underline{\varepsilon}}_s(\mathbf{v}) : \underline{\underline{\varepsilon}}_s(\mathbf{w})\,dA \;+\; \int_\Gamma \chi\,\rho_b\,(\mathbf{v}\cdot\mathbf{w})\,dA = -\int_\Gamma \sigma_a\,(\boldsymbol{\nabla}_s\cdot\mathbf{w})\,dA \;+\; \int_\Gamma \chi\,\rho_b\,(\dot{\mathbf{u}}\cdot\mathbf{w})\,dA \tag{70}$$

where $\underline{\underline{\varepsilon}}_s(\mathbf{w}) = \frac{1}{2}\left(\underline{\underline{\nabla_s \mathbf{w}}} + \underline{\underline{\nabla_s \mathbf{w}}}^T\right)$ and $\boldsymbol{n}_\Gamma$ is the outward unit normal to $\partial\Gamma$ lying in the tangent plane of the surface, and ds is the line element along $\partial\Gamma$.

In our implementation, the protrusive velocity is imposed as a Dirichlet condition on the cut boundary:

$$\mathbf{v} = \mathbf{v}_p \quad \text{on } \partial\Gamma, \tag{71}$$

so that the boundary term in (70) vanishes for admissible test functions $\mathbf{w}$ that satisfy $\mathbf{w} = 0$ on $\partial\Gamma$.

7.2.3 Biochemical species and MCA linker densities

For a generic scalar surface field c advected by a tangential velocity field $\mathbf{a}$ and diffusing on Γ,

$$\frac{\partial c}{\partial t} + \boldsymbol{\nabla}_s \cdot (c\,\mathbf{a}) = f(c,\ldots) + D\,\Delta_s c, \tag{72}$$

the conservative weak form with scalar test function w is

$$\int_\Gamma w\,\frac{\partial c}{\partial t}\,dA \;-\; \int_\Gamma c\,\mathbf{a}\cdot\boldsymbol{\nabla}_s w\,dA \;+\; \int_\Gamma D\,\boldsymbol{\nabla}_s c\cdot\boldsymbol{\nabla}_s w\,dA = \int_\Gamma w\,f(c,\ldots)\,dA \;+\; \int_{\partial\Gamma} w\,(D\,\boldsymbol{\nabla}_s c - c\,\mathbf{a})\cdot\boldsymbol{n}_\Gamma\,ds. \tag{73}$$

In our model, R and ρ are purely reaction–diffusion fields ($\mathbf{a} = \mathbf{0}$), while ρ_b is advected by the cortical flow $\mathbf{v}$ and ρ_u is advected by the membrane velocity $\dot{\mathbf{u}}$:

$$\partial_t R = F_R(\rho, \rho_b, \ldots) + D_R \Delta_s R, \tag{74}$$

$$\partial_t \rho = F_\rho(R, \sigma, \ldots) + D_\rho \Delta_s \rho, \tag{75}$$

$$\partial_t \rho_b + \boldsymbol{\nabla}_s \cdot (\rho_b \mathbf{v}) = k_{\text{on}} \rho_u - k_{\text{off}} \rho_b, \tag{76}$$

$$\partial_t \rho_u + \boldsymbol{\nabla}_s \cdot (\rho_u \dot{\mathbf{u}}) = -k_{\text{on}} \rho_u + k_{\text{off}} \rho_b + D\, \Delta_s \rho_u. \tag{77}$$

Boundary conditions and mass conservation. To ensure mass conservation for the transported species on the truncated surface, we impose no-flux boundary conditions on $\partial\Gamma$ in the sense of the total (advective + diffusive) surface flux:

$$(D\, \boldsymbol{\nabla}_s c - c\, \mathbf{a})\, |_{\partial\Gamma} \cdot \boldsymbol{n}_\Gamma = 0 \quad \text{on } \partial\Gamma, \tag{78}$$

for each advected/diffusing species c with advective velocity $\mathbf{a}$ (in particular for ρ_u, and for ρ_b when interpreted in conservative flux form). For R and ρ this reduces to the standard homogeneous Neumann condition $(\boldsymbol{\nabla}_s R)|_{\partial\Gamma} \cdot \boldsymbol{n}_\Gamma = 0$ and $(\boldsymbol{\nabla}_s \rho)|_{\partial\Gamma} \cdot \boldsymbol{n}_\Gamma = 0$.

Tension signal used in mechanochemical feedback. The production of ρ depends on a scalar tension measure σ that we defined as

$$\sigma \equiv \tau \cdot \left(\underline{\underline{N}}^{\text{mbr}} \cdot \tau \right) ., \tag{79}$$

7.3 Spatial discretization

We use a Cartesian discretization and Lagrange finite element spaces of polynomial order 2 for the scalar fields. The tangential vector fields $\mathbf{u}$ and $\mathbf{v}$ are discretized in a vector-valued finite element space consistent with the axisymmetric setting and the tangential constraint.

7.4 Temporal discretization

Time discretization is performed using a forward Euler scheme. For each variable y, we use

$$\frac{\partial y}{\partial t} \approx \frac{y^n - y^{n-1}}{\Delta t}, \tag{80}$$

where Δt is the time step. We choose Δt small enough to ensure stability and convergence. In practice, we tested a range of time steps and found that $\Delta t = 1$ provides good numerical accuracy while maintaining acceptable computational cost.

7.5 Numerical parameter values

There is a wide array of parameters to be set up in this model, which are summarized in the Supplementary Table 1. The membrane tension σ_0 [N/m] can be measured from membrane tether pulling [26, 7]. The friction coefficient is estimated as $\mu = \chi \rho_0$, where $\chi \approx 10^{-6}$ Pa.s.m [27] is the drag coefficient for an individual linker within the membrane and $\rho = 10^{14} m^{-2}$ is a typical surface density of linkers [28].

8 Two-dimensional results

Solving these non-linear equations on a curved surface introduces several key differences compared to the 1D model. Notably, continuity requirements at the poles preclude the imposition of Dirichlet boundary conditions. Consequently, two strategies arise: (1) modeling the protrusion as an explicit surface deformation (which significantly increases the mathematical and computational complexity), or (2) truncating the protruding region and applying Dirichlet boundary conditions at the resulting interface. As shown in Supplementary Text Figure 12, we have adopted the latter approach.

The results for the 2D axisymmetry shown in Supplementary Text Figure 12 are qualitatively consistent with the 1D model. Simulations outside the chemical bistable region can be polarized through mechanical feedback, mirroring the behavior of the 1D model. Furthermore, as in the 1D case, polarization without the mechanochemical feedback loop will eventually dissipate due to Rac and Rho diffusion.

Therefore, one can extrapolate that the phase diagrams computed in 1D would reproduce the same behaviour if using the 2D model.

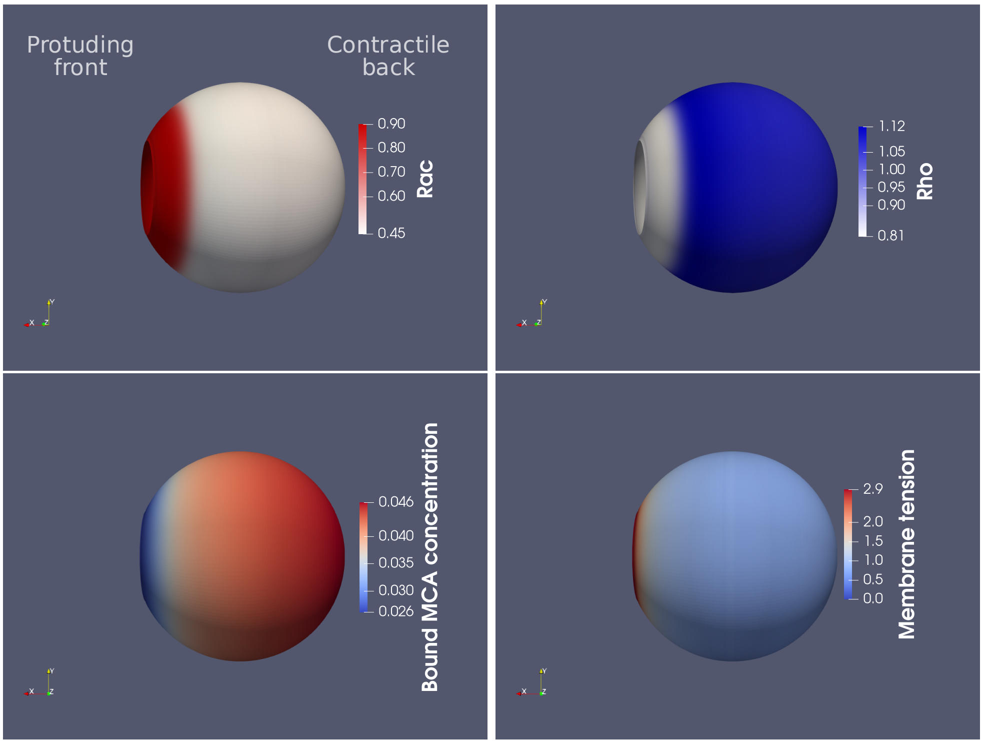

Supplementary Text Figure 12: Simulations in 2D axisymmetry using the mechanochemical model. Here, the local depletion of MCA promotes Rac activation, while the resulting increment in tension promotes Rho activation throughout the cell, establishing a mechanochemical feedback loop.

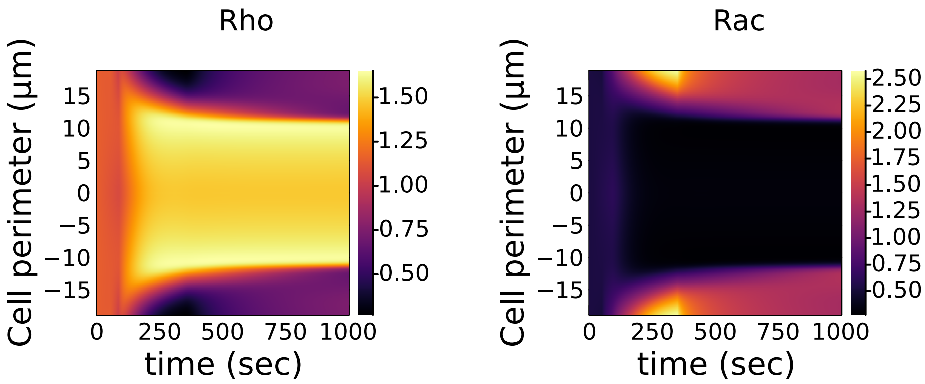

Supplementary Text Figure 13: Simulations in 2D axisymmetry using the mechanochemical model. Kymographs of the time evolution over the surface of the cell.

References

[1] M. Otsuji, S. Ishihara, C. Co, K. Kaibuchi, A. Mochizuki, and S. Kuroda, "A mass conserved reaction–diffusion system captures properties of cell polarity," *PLoS computational biology*, vol. 3, no. 6, p. e108, 2007.

[2] Y. Mori, A. Jilkine, and L. Edelstein-Keshet, "Wave-pinning and cell polarity from a bistable reaction-diffusion system," *Biophysical journal*, vol. 94, no. 9, pp. 3684–3697, 2008.

[3] C. Zmurchok, D. Bhaskar, and L. Edelstein-Keshet, "Coupling mechanical tension and gtpase signaling to generate cell and tissue dynamics," *Physical biology*, vol. 15, no. 4, p. 046004, 2018.

[4] K. H. Kopfer, W. Jäger, and F. Matthäus, "A mechanochemical model for rho gtpase mediated cell polarization," *Journal of theoretical biology*, vol. 504, p. 110386, 2020.

[5] L. K. Nguyen, B. N. Kholodenko, and A. Von Kriegsheim, "Rac1 and rhoa: Networks, loops and bistability," *Small GTPases*, vol. 9, no. 4, pp. 316–321, 2018.

[6] A. Jilkine, A. F. Marée, and L. Edelstein-Keshet, "Mathematical model for spatial segregation of the rho-family gtpases based on inhibitory crosstalk," *Bulletin of mathematical biology*, vol. 69, pp. 1943–1978, 2007.

[7] H. De Belly, S. Yan, H. B. da Rocha, S. Ichbiah, J. P. Town, P. J. Zager, D. C. Estrada, K. Meyer, H. Turlier, C. Bustamante *et al.*, "Cell protrusions and contractions generate long-range membrane tension propagation," *Cell*, vol. 186, no. 14, pp. 3049–3061, 2023.

[8] G. Salbreux, J. Prost, and J.-F. Joanny, "Hydrodynamics of cellular cortical flows and the formation of contractile rings," *Physical review letters*, vol. 103, no. 5, p. 058102, 2009.

[9] M. Mayer, M. Depken, J. S. Bois, F. Jülicher, and S. W. Grill, "Anisotropies in cortical tension reveal the physical basis of polarizing cortical flows," *Nature*, vol. 467, no. 7315, pp. 617–621, 2010.

[10] H. Turlier, B. Audoly, J. Prost, and J.-F. Joanny, "Furrow constriction in animal cell cytokinesis," *Biophysical journal*, vol. 106, no. 1, pp. 114–123, 2014.

[11] H. Borja Da Rocha, J. Bleyer, and H. Turlier, "A viscous active shell theory of the cell cortex," *Journal of the Mechanics and Physics of Solids*, vol. 164, p. 104876, 2022.

[12] Z. Shi, Z. T. Graber, T. Baumgart, H. A. Stone, and A. E. Cohen, "Cell membranes resist flow," *Cell*, vol. 175, no. 7, pp. 1769–1779, 2018.

[13] E. Laborie and A. Callan-Jones, "Power-law decay of force on cell membrane tethers reflects long-ranged relaxation of membrane tension," *arXiv preprint arXiv:2502.05601*, 2025.

[14] T. S. Gardner, C. R. Cantor, and J. J. Collins, "Construction of a genetic toggle switch in escherichia coli," *Nature*, vol. 403, no. 6767, pp. 339–342, 2000.

[15] A. R. Houk, A. Jilkine, C. O. Mejean, R. Boltyanskiy, E. R. Dufresne, S. B. Angenent, S. J. Altschuler, L. F. Wu, and O. D. Weiner, "Membrane tension maintains cell polarity by confining signals to the leading edge during neutrophil migration," *Cell*, vol. 148, no. 1, pp. 175–188, 2012.

[16] R. G. Fehon, A. I. McClatchey, and A. Bretscher, "Organizing the cell cortex: the role of erm proteins," *Nature reviews Molecular cell biology*, vol. 11, no. 4, pp. 276–287, 2010.

[17] C. D. Nobes and A. Hall, "Rho gtpases control polarity, protrusion, and adhesion during cell movement," *The Journal of cell biology*, vol. 144, no. 6, pp. 1235–1244, 1999.

[18] P. Bohec, D. Khoromskaia, M. Kelkar, E. Ferber, G. Duprez, G. Lavoie, L. Valon, P. P. Roux, G. Salbreux, and G. Charras, "Control of cellular cortical tension and shape by rhogtpase signalling," *bioRxiv*, pp. 2025–12, 2025.

[19] D. Raucher and M. P. Sheetz, "Cell spreading and lamellipodial extension rate is regulated by membrane tension," *The Journal of cell biology*, vol. 148, no. 1, pp. 127–136, 2000.

[20] H. Turlier, D. A. Fedosov, B. Audoly, T. Auth, N. S. Gov, C. Sykes, J.-F. Joanny, G. Gompper, and T. Betz, “Equilibrium physics breakdown reveals the active nature of red blood cell flickering,” *Nature physics*, vol. 12, no. 5, pp. 513–519, 2016.

[21] M. A. Biot, *Mechanics of incremental deformations*, 1965.

[22] Y. Kantor, “Entropic elasticity of tethered solids,” *Physical Review A*, vol. 39, no. 12, p. 6582, 1989.

[23] E. Neiva and H. Turlier, “Unfitted finite element modelling of surface-bulk viscous flows in animal cells,” *arXiv preprint arXiv:2505.05723*, 2025.

[24] S. Badia and F. Verdugo, “Gridap: An extensible finite element toolbox in julia,” *Journal of Open Source Software*, vol. 5, no. 52, p. 2520, 2020. [Online]. Available: https://doi.org/10.21105/joss.02520

[25] F. Verdugo and S. Badia, “The software design of gridap: A finite element package based on the julia JIT compiler,” *Computer Physics Communications*, vol. 276, p. 108341, Jul. 2022. [Online]. Available: https://doi.org/10.1016/j.cpc.2022.108341

[26] P. Sens and J. Plastino, “Membrane tension and cytoskeleton organization in cell motility,” *Journal of Physics: Condensed Matter*, vol. 27, no. 27, p. 273103, 2015.

[27] F. Brochard-Wyart, N. Borghi, D. Cuvelier, and P. Nassoy, “Hydrodynamic narrowing of tubes extruded from cells,” *Proceedings of the National Academy of Sciences*, vol. 103, no. 20, pp. 7660–7663, 2006. [Online]. Available: https://www.pnas.org/doi/abs/10.1073/pnas.0602012103

[28] R. Alert, J. Casademunt, J. Brugués, and P. Sens, “Model for probing membrane-cortex adhesion by micropipette aspiration and fluctuation spectroscopy,” *Biophysical journal*, vol. 108, no. 8, pp. 1878–1886, 2015.
