# Supplementary material for: Long-range mutual activation establishes Rho and Rac polarity during cell migration
Source: Nat Cell Biol. 2026 Jun 10;28(6):1244–57. doi: 10.1038/s41556-026-01965-1 (PMC13279283; doi:10.1038/s41556-026-01965-1)
Supplement: Supplementary file 3 — Peer Review File [file 41556_2026_1965_MOESM3_ESM.pdf]

nature portfolio

Peer Review File

Long range mutual activation establishes Rho and Rac polarity during cell migration

Corresponding Author: Professor Orion Weiner

Version 0:

Decision Letter:

*Please delete the link to your author homepage if you wish to forward this email to co-authors.

Dear Professor Weiner,

Please accept our sincerest apologies for the length of time your manuscript has been under consideration at our journal. Despite our best efforts, we have been unable to receive the comments of reviewer #3, who is an expert in signalling and modelling.

Please note that if/when we do receive the comments of the missing referee, we will pass them on to you; should we be in touch with the missing comments, your revised manuscript should address all of the referees' concerns including those sent subsequently.

Your manuscript, "Long range mutual activation establishes Rho and Rac polarity during cell migration", has now been seen by 2 referees, who are experts in cell migration, mechanobiology, and modelling (referee 1);and RhoGTPases (referee 2). As you will see from their comments (attached below) they find this work of potential interest, but have raised substantial concerns, which in our view would need to be addressed with considerable revisions before we can consider publication in Nature Cell Biology.

Nature Cell Biology editors discuss the referee reports in detail within the editorial team, including the chief editor, to identify key referee points that should be addressed with priority, and requests that are overruled as being beyond the scope of the current study. To guide the scope of the revisions, I have listed these points below. We are committed to providing a fair and constructive peer-review process, so please feel free to contact me if you would like to discuss any of the referee comments further.

In particular, it would be essential to:

A) Further experimentally assess the role of the different RhoGTPases (Reviewers #1 and #2) as well as have additional tools to further validate the claims of the RhoGTPases (both reviewers) with additional data

B) Further experimentally test the role of various phosphoinositides in your proposed mechanism (both Reviewers)

C) Provide further experimental data on the precise role and location o frelevant MTOR activity (Reviewer #2)

D) All other referee concerns pertaining to strengthening existing data, providing controls, methodological details, clarifications and textual changes, should also be addressed.

E) Finally please pay close attention to our guidelines on statistical and methodological reporting (listed below) as failure to do so may delay the reconsideration of the revised manuscript. In particular please provide:

- a Supplementary Figure including unprocessed images of all gels/blots in the form of a multi-page pdf file. Please ensure that blots/gels are labeled and the sections presented in the figures are clearly indicated.

- a Supplementary Table including all numerical source data in Excel format, with data for different figures provided as different sheets within a single Excel file. The file should include source data giving rise to graphical representations and statistical descriptions in the paper and for all instances where the figures present representative experiments of multiple independent repeats, the source data of all repeats should be provided.

We would be happy to consider a revised manuscript that would satisfactorily address these points, unless a similar paper is published elsewhere, or is accepted for publication in Nature Cell Biology in the meantime.

When revising the manuscript please:

- ensure that it conforms to our format instructions and publication policies (see below and https://www.nature.com/nature/for-authors).

- provide a point-by-point rebuttal to the full referee reports verbatim, as provided at the end of this letter.

- for any revision that includes light microscopy data, we ask our authors to please include a completed light microscopy reporting table https://www.nature.com/documents/Light_microscopy_reporting_table.xlsx to ensure the methods are described thoroughly. The table will be available to reviewers and ultimately published should the manuscript be accepted at the journal.

- provide the completed Reporting Summary (found here https://www.nature.com/documents/nr-reporting-summary.pdf). This is essential for reconsideration of the manuscript will be available to editors and referees in the event of peer review. For more information see http://www.nature.com/authors/policies/availability.html or contact me.

When submitting the revised version of your manuscript, please pay close attention to our href="https://www.nature.com/nature-portfolio/editorial-policies/image-integrity">Digital Image Integrity Guidelines. and to the following points below:

-- that unprocessed scans are clearly labelled and match the gels and western blots presented in figures.
-- that control panels for gels and western blots are appropriately described as loading on sample processing controls
-- all images in the paper are checked for duplication of panels and for splicing of gel lanes.

Finally, please ensure that you retain unprocessed data and metadata files after publication, ideally archiving data in perpetuity, as these may be requested during the peer review and production process or after publication if any issues arise.

Nature Cell Biology is committed to improving transparency in authorship. As part of our efforts in this direction, we are now requesting that all authors identified as ‘corresponding author’ on published papers create and link their Open Researcher and Contributor Identifier (ORCID) with their account on the Manuscript Tracking System (MTS), prior to acceptance. ORCID helps the scientific community achieve unambiguous attribution of all scholarly contributions. You can create and link your ORCID from the home page of the MTS by clicking on ‘Modify my Springer Nature account’. For more information please visit please visit www.springernature.com/orcid.

This journal strongly supports public availability of data. Please place the data used in your paper into a public data repository, or alternatively, present the data as Supplementary Information. If data can only be shared on request, please explain why in your Data Availability Statement, and also in the correspondence with your editor. Please note that for some data types, deposition in a public repository is mandatory - more information on our data deposition policies and available repositories appears below.

Please submit the revised manuscript files and the point-by-point rebuttal to the referee comments using this link:

Link Redacted

*This url links to your confidential home page and associated information about manuscripts you may have submitted or be reviewing for us. If you wish to forward this email to co-authors, please delete the link to your homepage.

We would like to receive a revised submission within six months.

We hope that you will find our referees' comments, and editorial guidance helpful. Please do not hesitate to contact me if there is anything you would like to discuss.

Best wishes,

Daryl

Daryl Jason Verzosa David, PhD

Senior Editor, Nature Cell Biology
Advisory Editor, npj Biological Physics and Mechanics

Nature Portfolio

Heidelberger Platz 3, 14197 Berlin, Germany
Email: daryl.david@nature.com
ORCID: https://orcid.org/0000-0002-9253-4805

Reviewers' Comments:

Reviewer #1 (Remarks to the Author):

In this manuscript, De Belly et al. investigate the mechanisms underlying the polarization of the GTPases Rho and Rac at opposite poles of migrating cells. Although previous studies have explored potential polarization mechanisms, this work provides new interesting insights through a combination of optical stimulation techniques, live protein biosensors and mathematical modeling. Specifically, the authors argue that the polarization of one GTPase facilitates reciprocal polarization of the other at the opposite cellular pole via a mechanism involving membrane tension-based activation of mTORC2 and cortical flow-based regulation of phosphoinositide signaling. Overall, this excellent study advances our understanding of the coordinated spatial regulation of Rho and Rac during cell migration. However, a few key issues need to be addressed prior to publication.

Major Comments:
1. In this manuscript, the authors used an opto-iSH2, which regulates PI3K/Akt. However, optogenetic activation of PI3K influences various downstream signals including Rac. Therefore, the authors should verify their key findings using a more targeted optogenetic tool for Rac, such as opto-TIAM (PMID: 37449837).

2. The authors show that hypotonic shock increases Rho activity through elevated membrane tension, independently of actin-based protrusions. While mTORC is identified as a potential mediator of this response, it is worth noting that cell swelling upon hypotonic shock involves water influx through aquaporins and the ion transporter NHE1, as reported by the authors and others (PMID: 24726433). The authors should investigate whether Rho activation is also functionally linked to these volume-regulating channels.

3. According to Figure 3C, stable blebs form at the opposite end of RhoA stimulation. However, high RhoA activity also induces the formation of small blebs at the cell rear (Figure 4b). The stable blebs formed after adding Lat B show robust Rac enrichment (Fig. S5D and S6J). Do these large blebs completely lack RhoA? Also, LatB-induced blebs do not exhibit Rac enrichment following Duvelisib treatment (Fig. S6J). Are they also devoid of RhoA?

4. In Figure 4, the authors show that optogenetic activation of RhoA at the cell rear depletes MCA proteins at the front, releasing PIP2, which is then converted by PI3K into PIP3 to activate Rac. The authors should use a PIP3 reporter to validate this mechanism.

5. In Figure 4D, the authors report PIP2 polarization at the cell front following opto-Rho stimulation. However, previously published work (PMID: 36202973) shows PIP2 enrichment at the rear and PIP3 localization at the front in both Dictyostelium and RAW 264.7 macrophages. How do the authors reconcile their findings with prior work?

6. Based on the MCA-PIP2-Rac model, how do the authors explain the data reported in Figures 4C and 4F, where ezrin remains localized at the rear for over 300 seconds after stimulation, while the increase in Rac activity lasts less than 30 seconds?

7. In Figure 5, the authors aim to extend their mechanism to non-migratory cells. To validate the Rho-Blebbing-PIP2-Rac axis cells, they should examine Rac activity instead of only PIP2 levels.
a. In Figure 5, they should consider using opto-Rac and opto-Rho in one cell type, rather than relying solely on hypotonic shock and LatB treatment.

8. The authors should validate some of the important experiments using anillin-RBD with a RhoA-2xRBD-tdTomato sensor (PMID: 34357388).

9. The authors should consider using a fluorophore lifetime-based membrane tension sensor (PMID: 36323783) to validate their claims in the paper.

Reviewer #1 (Remarks on Protocol(s)):

Appropriate.

Reviewer #2 (Remarks to the Author):

This study by De Belly and colleagues addresses an important unanswered question in the regulation of cellular

polarisation: how the GTPases RAC and RHO regulate each other from spatially distinct regions of the cell. They report a mechanism by which membrane tension caused by RAC-induced membrane projections causes the activation of RHO on the membrane at the opposite side of the cell, an effect they report to be dependent on MTORC2 signalling. They also report an inverse regulation where RAC is activated at the leading edge in response to RHO activation at the rear of the cell and report that this is due to increases in PIP¬2 concentrations leading to a PI3K dependent activation of RAC. The authors then demonstrate the presence of these reported mechanisms in epithelial cells and primary T-cells.
The work is explained well (there are some great visual aids throughout the figures and well-annotated legends) and understanding the long-range regulation of these GTPases by each other is important for research into normal development and multiple diseases including immune deficiencies and cancer. However, I have concerns about the mechanisms they report, and the tools used in this work. My specific concerns are detailed below:
1. The primary tools used in this work are fluorescent protein-tagged binding domains that bind to the active form of specific GTPases. It is, however, unclear which GTPases they are referring to in this paper as the specificity of these probes is broader than indicated. For example, the Pak Binding Domain (PBD) will bind to RAC1, RAC2, RAC3 and CDC42. However, the authors only refer to "RAC" without specifying which RAC family member. This is important because RAC1 and 2 are both expressed and have distinct roles in immune cells. The authors therefore need to demonstrate which RAC family member is performing the reported function and be specific in the manuscript, that is to say replace "RAC" with reference to a specific RAC family member. They also need to discount the role of CDC42 as the probe will also be binding CDC42-GTP. Likewise, simply writing "RHO" is not informative; are the authors referring to RHOA? Does this probe bind any other RHO family members? As the work is centred on the use of these probes, it is essential that their specificity is confirmed and the specific proteins performing the functions identified referred to throughout.
2. The mechanism the authors show in figure 2 for membrane tension activating RHO in a RICTOR-dependent (mTORC2) manner needs further elaboration. MTOR signalling to RHOA has been reported before, it is therefore important to deduce whether this function of MTOR occurs throughout the cell, the periphery of the cell (locally activating RHO) or alternatively at the site of membrane protrusion.
3. The authors report that RHO activation leads to blebbing at the opposite side of the cell to cause RAC activation. They identify a mechanism where Membrane-to-Cortex-Attachment proteins dissociate from the membrane and move to the periphery causing blebbing and a local increase in PIP2 at the front of the cell, leading in turn to PI3K activity and RAC activation. This mechanism is not convincing. The loss of EZR mCherry at the leading edge occurs on a longer time scale than the generation of PIP2, which is very rapid (fig 4B-E). This implies that the PIP2 increase is occurring prior to the loss of EZR at this site. The connection to PI3K and RAC activation is also not robust. The inference that PI3K is activated by an excess of PIP2 is not supported by data and not a recognised mechanism of PI3K activation. To support this, the authors need to demonstrate that a local increase in PIP2 in this system is required and sufficient to activate PI3K, leading to a localised increase in PIP3 and subsequent RAC activation. The cause and effect of this mechanism is also not clear. For example, it is conceivable that an intracellular signalling cascade caused by RHOA activation results in the PI3K dependent activation of RAC1 on the opposite side of the cell which subsequently triggers blebbing and relocalisation of EZR. In my mind this is the most likely mechanism of action and needs to be investigated by the authors.
4. The authors seek to demonstrate the identified mechanisms in epithelial cells. However, there are some problems with this approach. Firstly, the origin of HEK293 cells is debatable and considered by many not to be epithelial. Moreover, neither HEK293 or HeLa cells polarise well in culture. Therefore, these are not appropriate cells to study the polarisation of epithelial cells. Furthermore, the approach used, where cells are in suspension, has problems due to the induction of anoikis in epithelial cells. HeLa cells are more resistant to anoikis than primary epithelial cells, yet they still exhibit some sensitivity. When applying this approach to an appropriate epithelial cell line anoikis will be a prominent issue. It is notable that RHOA/ROCK signalling is known to have roles in anoikis and inhibition of this pathway is often used to prevent it. Therefore, the authors need to demonstrate that the identified mechanisms occur in adherent epithelial cells and that these mechanisms are required for the maintenance of epithelial cell polarisation.
5. The authors then suggest that these mechanisms occur in primary T-cells and show that the inhibition of Gαi or actomyosin can inhibit T-cell migration. With regard to this, it is not novel that inhibiting Gαi signalling or actomyosin prevents migration and these experiments do not exclusively support the reported mechanisms functioning in T-cells.

Minor points
A. The authors refer to RAC and RHO activity. However, the probes they use sense the activation of the GTPases and not their enzymatic activity. This should be changed to "activation" or "signalling activity".
B. Pak-PBD = Pak-Pak-binding domain. I assume the authors simply mean PBD or alternatively, Pak-CRIB.
C. In fig C and D of Fig 1, the increase in signal for the PBD probe is clearly increasing at the front, however, the loss in signal at the back is not evident in the representative image.
D. (E) (F) onward are wrongly written in the legend for Fig. 4.

Reviewer #2 (Remarks on Protocol(s)):

From a quick look they seem to have the expected level of detail.

GUIDELINES FOR MANUSCRIPT SUBMISSION TO NATURE CELL BIOLOGY

READABILITY OF MANUSCRIPTS – Nature Cell Biology is read by cell biologists from diverse backgrounds, many of whom are not native English speakers. Authors should aim to communicate their findings clearly, explaining technical jargon that might be unfamiliar to non-specialists, and avoiding non-standard abbreviations. Titles and abstracts should concisely communicate the main findings of the study, and the background, rationale, results and conclusions should be clearly explained in the manuscript in a manner accessible to a broad cell biology audience. Nature Cell Biology uses British spelling.

MANUSCRIPT FORMAT – please follow the guidelines listed in our Guide to Authors regarding manuscript formats at Nature Cell Biology.

TITLE – should be no more than 100 characters including spaces, without punctuation and avoiding technical terms, abbreviations, and active verbs..

AUTHOR NAMES – should be given in full.

AUTHOR AFFILIATIONS – should be denoted with numerical superscripts (not symbols) preceding the names. Full addresses should be included, with US states in full and providing zip/post codes. The corresponding author is denoted by: "Correspondence should be addressed to [initials]."

ABSTRACT AND MAIN TEXT – please follow the guidelines that are specific to the format of your manuscript, as listed in our Guide to Authors (http://www.nature.com/ncb/pdf/ncb_gta.pdf) Briefly, Nature Cell Biology Articles, Resources and Technical Reports have 3500 words, including a 150 word abstract, and the main text is subdivided in Introduction, Results, and Discussion sections. Nature Cell Biology Letters have up to 2500 words, including a 180 word introductory paragraph (abstract), and the text is not subdivided in sections.

ACKNOWLEDGEMENTS – should be kept brief. Professional titles and affiliations are unnecessary. Grant numbers can be listed.

AUTHOR CONTRIBUTIONS – must be included after the Acknowledgements, detailing the contributions of each author to the paper (e.g. experimental work, project planning, data analysis etc.). Each author should be listed by his/her initials.

FINANCIAL AND NON-FINANCIAL COMPETING INTERESTS – the authors must include one of three declarations: (1) that they have no financial and non-financial competing interests; (2) that they have financial and non-financial competing interests; or (3) that they decline to respond, after the Author Contributions section. This statement will be published with the article, and in cases where financial and non-financial competing interests are declared, these will be itemized in a web supplement to the article. For further details please see https://www.nature.com/licenceforms/nrg/competing-interests.pdf.

REFERENCES – are limited to a total of 70 for Articles, Resources, Technical Reports; and 40 for Letters. This includes references in the main text and Methods combined. References must be numbered sequentially as they appear in the main text, tables and figure legends and Methods and must follow the precise style of Nature Cell Biology references. References only cited in the Methods should be numbered consecutively following the last reference cited in the main text. References only associated with Supplementary Information (e.g. in supplementary legends) do not count toward the total reference limit and do not need to be cited in numerical continuity with references in the main text. Only published papers can be cited, and each publication cited should be included in the numbered reference list, which should include the manuscript titles. Footnotes are not permitted.

METHODS – Nature Cell Biology publishes methods online. The methods section should be provided as a separate Word document, which will be copyedited and appended to the manuscript PDF, and incorporated within the HTML format of the paper.

Methods should be written concisely, but should contain all elements necessary to allow interpretation and replication of the results. As a guideline, Methods sections typically do not exceed 3,000 words. The Methods should be divided into subsections listing reagents and techniques. When citing previous methods, accurate references should be provided and any alterations should be noted. Information must be provided about: antibody dilutions, company names, catalogue numbers and clone numbers for monoclonal antibodies; sequences of RNAi and cDNA probes/primers or company names and catalogue numbers if reagents are commercial; cell line names, sources and information on cell line identity and authentication. Animal studies and experiments involving human subjects must be reported in detail, identifying the committees approving the protocols. For studies involving human subjects/samples, a statement must be included confirming that informed consent was obtained. Statistical analyses and information on the reproducibility of experimental results should be provided in a section titled “Statistics and Reproducibility”.

All Nature Cell Biology manuscripts submitted on or after March 21 2016 must include a Data availability statement as a separate section after Methods but before references, under the heading "Data Availability”. . For Springer Nature policies on data availability see http://www.nature.com/authors/policies/availability.html; for more information on this particular policy see http://www.nature.com/authors/policies/data/data-availability-statements-data-citations.pdf. The Data availability statement should include:

• Accession codes for primary datasets (generated during the study under consideration and designated as "primary accessions") and secondary datasets (published datasets reanalysed during the study under consideration, designated as "referenced accessions"). For primary accessions data should be made public to coincide with publication of the manuscript. A list of data types for which submission to community-endorsed public repositories is mandated (including sequence, structure, microarray, deep sequencing data) can be found here http://www.nature.com/authors/policies/availability.html#data.

• Unique identifiers (accession codes, DOIs or other unique persistent identifier) and hyperlinks for datasets deposited in an approved repository, but for which data deposition is not mandated (see here for details http://www.nature.com/sdata/data-policies/repositories).

• At a minimum, please include a statement confirming that all relevant data are available from the authors, and/or are included with the manuscript (e.g. as source data or supplementary information), listing which data are included (e.g. by figure panels and data types) and mentioning any restrictions on availability.

• If a dataset has a Digital Object Identifier (DOI) as its unique identifier, we strongly encourage including this in the Reference list and citing the dataset in the Methods.

We recommend that you upload the step-by-step protocols used in this manuscript to protocols.io. More details can found at https://www.protocols.io/help/publish-articles.

DISPLAY ITEMS – main display items are limited to 6-8 main figures and/or main tables for Articles, Resources, Technical Reports; and 5 main figures and/or main tables for Letters. For Supplementary Information see below.

FIGURES – Colour figure publication costs $600 for the first, and $300 for each subsequent colour figure. All panels of a multi-panel figure must be logically connected and arranged as they would appear in the final version. Unnecessary figures and figure panels should be avoided (e.g. data presented in small tables could be stated briefly in the text instead).

All imaging data should be accompanied by scale bars, which should be defined in the legend.
Cropped images of gels/blots are acceptable, but need to be accompanied by size markers, and to retain visible background signal within the linear range (i.e. should not be saturated). The boundaries of panels with low background have to be demarked with black lines. Splicing of panels should only be considered if unavoidable, and must be clearly marked on the figure, and noted in the legend with a statement on whether the samples were obtained and processed simultaneously. Quantitative comparisons between samples on different gels/blots are discouraged; if this is unavoidable, it should only be performed for samples derived from the same experiment with gels/blots were processed in parallel, which needs to be stated in the legend.

Figures should be provided at approximately the size that they are to be printed at (single column is 86 mm, double column is 170 mm) and should not exceed an A4 page (8.5 x 11"). Reduction to the scale that will be used on the page is not necessary, but multi-panel figures should be sized so that the whole figure can be reduced by the same amount at the smallest size at which essential details in each panel are visible. In the interest of our colour-blind readers we ask that you avoid using red and green for contrast in figures. Replacing red with magenta and green with turquoise are two possible colour-safe alternatives. Lines with widths of less than 1 point should be avoided. Sans serif typefaces, such as Helvetica (preferred) or Arial should be used. All text that forms part of a figure should be rewritable and removable.

We accept files from the following graphics packages in either PC or Macintosh format:

- For line art, graphs, charts and schematics we prefer Adobe Illustrator (.AI), Encapsulated PostScript (.EPS) or Portable Document Format (.PDF). Files should be saved or exported as such directly from the application in which they were made, to allow us to restyle them according to our journal house style.

- We accept PowerPoint (.PPT) files if they are fully editable. However, please refrain from adding PowerPoint graphical effects to objects, as this results in them outputting poor quality raster art. Text used for PowerPoint figures should be Helvetica (preferred) or Arial.

- We do not recommend using Adobe Photoshop for designing figures, but we can accept Photoshop generated (.PSD or .TIFF) files only if each element included in the figure (text, labels, pictures, graphs, arrows and scale bars) are on separate layers. All text should be editable in 'type layers' and line-art such as graphs and other simple schematics should be preserved and embedded within 'vector smart objects' - not flattened raster/bitmap graphics.

- Some programs can generate Postscript by 'printing to file' (found in the Print dialogue). If using an application not listed above, save the file in PostScript format or email our Art Editor, Allen Beattie for advice (a.beattie@nature.com).

Regardless of format, all figures must be vector graphic compatible files, not supplied in a flattened raster/bitmap graphics format, but should be fully editable, allowing us to highlight/copy/paste all text and move individual parts of the figures (i.e. arrows, lines, x and y axes, graphs, tick marks, scale bars etc.). The only parts of the figure that should be in pixel raster/bitmap format are photographic images or 3D rendered graphics/complex technical illustrations.

All placed images (i.e. a photo incorporated into a figure) should be on a separate layer and independent from any superimposed scale bars or text. Individual photographic images must be a minimum of 300+ DPI (at actual size) or kept constant from the original picture acquisition and not decreased in resolution post image acquisition. All colour artwork should be RGB format.

EXTENDED DATA FIGURES - When re-submitting your manuscript, please ensure that any supplementary figures and tables that are crucial to the manuscript's conclusions are converted into Extended Data figures and tables to increase visibility of these data. Extended Data figures and tables are online-only (present in the online PDF and full-text HTML versions of the paper), peer-reviewed display items that provide essential background to the article but are not included in the main article due to space constraints. A maximum of ten Extended Data display items (figures and tables) is permitted.

FIGURE LEGENDS – must not exceed 350 words for each figure to allow fit on a single printed NCB page together with the figure. They must include a brief title for the whole figure, and short descriptions of each panel with definitions of the symbols used, but without detailing methodology.

TABLES – main tables should be provided as individual Word files, together with a brief title and legend. For supplementary tables see below.

SUPPLEMENTARY INFORMATION – Supplementary information is material directly relevant to the conclusion of a paper, but which cannot be included in the printed version in order to keep the manuscript concise and accessible to the general reader. Supplementary information is an integral part of a Nature Cell Biology publication and should be prepared and presented with as much care as the main display item, but it must not include non-essential data or text, which may be removed at the editor's discretion. All supplementary material is fully peer-reviewed and published online as part of the HTML version of the manuscript. Supplementary Figures and Supplementary Notes are appended at the end of the main PDF of the published manuscript.

Supplementary items should relate to a main text figure, wherever possible, and should be mentioned sequentially in the main manuscript, designated as Supplementary Figure, Table, Video, or Note, and numbered continuously (e.g. Supplementary Figure 1, Supplementary Figure 2, Supplementary Table 1, Supplementary Table 2 etc.).

Unprocessed scans of all key data generated through electrophoretic separation techniques need to be presented in a supplementary figure that should be labelled and numbered as the final supplementary figure, and should be mentioned in every relevant figure legend. This figure does not count towards the total number of figures and is the only figure that can be displayed over multiple pages, but should be provided as a single file, in PDF or TIFF format. Data in this figure can be displayed in a relatively informal style, but size markers and the figures panels corresponding to the presented data must be indicated.

The total number of Supplementary Figures (not including the "unprocessed scans" Supplementary Figure) should not exceed the number of main display items (figures and/or tables (see our Guide to Authors and March 2012 editorial http://www.nature.com/ncb/authors/submit/index.html#suppinfo; http://www.nature.com/ncb/journal/v14/n3/index.html#ed). No restrictions apply to Supplementary Tables or Videos, but we advise authors to be selective in including supplemental data.

Each Supplementary Figure should be provided as a single page and as an individual file in one of our accepted figure formats and should be presented according to our figure guidelines (see above). Supplementary Tables should be provided as individual Excel files. Supplementary Videos should be provided as .avi or .mov files up to 50 MB in size. Supplementary Figures, Tables and Videos much be accompanied by a separate Word document including titles and legends.

GUIDELINES FOR EXPERIMENTAL AND STATISTICAL REPORTING

REPORTING REQUIREMENTS – We are trying to improve the quality of methods and statistics reporting in our papers. To that end, we are now asking authors to complete a reporting summary that collects information on experimental design and reagents. The Reporting Summary can be found here https://www.nature.com/documents/nr-reporting-summary.pdf)If you would like to reference the guidance text as you complete the template, please access these flattened versions at http://www.nature.com/authors/policies/availability.html.

STATISTICS – Wherever statistics have been derived the legend needs to provide the n number (i.e. the sample size used to derive statistics) as a precise value (not a range), and define what this value represents. Error bars need to be defined in the legends (e.g. SD, SEM) together with a measure of centre (e.g. mean, median). Box plots need to be defined in terms of minima, maxima, centre, and percentiles. Ranges are more appropriate than standard errors for small data sets. Wherever statistical significance has been derived, precise p values need to be provided and the statistical test used needs to be stated in the legend. Statistics such as error bars must not be derived from n<3. For sample sizes of n<5 please plot the individual data points rather than providing bar graphs. Deriving statistics from technical replicate samples, rather than biological replicates is strongly discouraged. Wherever statistical significance has been derived, precise p values need to be provided and the statistical test stated in the legend.

Information on how many times each experiment was repeated independently with similar results needs to be provided in

the legends and/or Methods for all experiments, and in particular wherever representative experiments are shown.

We strongly recommend the presentation of source data for graphical and statistical analyses as a separate Supplementary Table, and request that source data for all independent repeats are provided when representative experiments of multiple independent repeats, or averages of two independent experiments are presented. This supplementary table should be in Excel format, with data for different figures provided as different sheets within a single Excel file. It should be labelled and numbered as one of the supplementary tables, titled “Statistics Source Data”, and mentioned in all relevant figure legends.

--------- Please don't hesitate to contact NCB@nature.com should you have queries about any of the above requirements ---------

Version 1:

Decision Letter:

Our ref: NCB-A57820A

24th February 2026

Dear Dr. Weiner,

Thank you for submitting your revised manuscript "Long range mutual activation establishes Rho and Rac polarity during cell migration" (NCB-A57820A). It has now been seen by the original referees and their comments are below. The reviewers find that the paper has improved in revision, and therefore we'll be happy in principle to publish it in Nature Cell Biology, pending minor revisions to comply with our editorial and formatting guidelines.

We are now performing detailed checks on your paper and will send you a checklist detailing our editorial and formatting requirements in about a week. Please do not upload the final materials and make any revisions until you receive this additional information from us.

Thank you again for your interest in Nature Cell Biology. Please do not hesitate to contact me if you have any questions.

Sincerely,

Daryl

Daryl Jason Verzosa David, PhD

Senior Editor, Nature Cell Biology
Advisory Editor, npj Biological Physics and Mechanics
Nature Portfolio

Heidelberger Platz 3, 14197 Berlin, Germany
Email: daryl.david@nature.com
ORCID: https://orcid.org/0000-0002-9253-4805

Reviewer #1 (Remarks to the Author):

The authors have satisfactorily addressed this reviewer's remarks. As such, our recommendation is: ACCEPT.

Reviewer #2 (Remarks to the Author):

The authors have performed a lot of work and responded to this reviewer's concerns satisfactorily.

Reviewer #2 (Remarks on Protocol(s)):

No for this submission, but yes for the previous one.

Version 2:

Decision Letter:

Dear Dr Weiner,

I am pleased to inform you that your manuscript, "Long range mutual activation establishes Rho and Rac polarity during cell migration", has now been accepted for publication in Nature Cell Biology.

Thank you for sending us the final manuscript files to be processed for print and online production, and for returning the manuscript checklists and other forms. Your manuscript will now be passed to our production team who will be in contact with you if there are any questions with the production quality of supplied figures and text.

Over the next few weeks, your paper will be copyedited to ensure that it conforms to Nature Cell Biology style. Once your paper is typeset, you will receive an email with a link to choose the appropriate publishing options for your paper and our Author Services team will be in touch regarding any additional information that may be required.

After the grant of rights is completed, you will receive a link to your electronic proof via email with a request to make any corrections within 48 hours. If, when you receive your proof, you cannot meet this deadline, please inform us at rjsproduction@springernature.com immediately.

You will not receive your proofs until the publishing agreement has been received through our system.

Due to the importance of these deadlines, we ask that you please let us know now whether you will be difficult to contact over the next month. If this is the case, we ask you provide us with the contact information (email, phone and fax) of someone who will be able to check the proofs on your behalf, and who will be available to address any last-minute problems.

If you have any questions about our publishing options, costs, Open Access requirements, or our legal forms, please contact ASJournals@springernature.com

Once your paper has been scheduled for online publication, the Nature press office will be in touch to confirm the details. An online order form for reprints of your paper is available at https://www.nature.com/reprints/author-reprints.html. All co-authors, authors' institutions and authors' funding agencies can order reprints using the form appropriate to their geographical region.

Publication is conditional on the manuscript not being published elsewhere and on there being no announcement of this work to any media outlet until the online publication date in Nature Cell Biology.

You may wish to make your media relations office aware of your accepted publication, in case they consider it appropriate to organize some internal or external publicity. Once your paper has been scheduled you will receive an email confirming the publication details. This is normally 3-4 working days in advance of publication. If you need additional notice of the date and time of publication, please let the production team know when you receive the proof of your article to ensure there is sufficient time to coordinate. Further information on our embargo policies can be found here: https://www.nature.com/authors/policies/embargo.html

Please note that *Nature Cell Biology* is a Transformative Journal (TJ). Authors may publish their research with us through the traditional subscription access route or make their paper immediately open access through payment of an article-processing charge (APC). Authors will not be required to make a final decision about access to their article until it has been accepted. Find out more about Transformative Journals

Authors may need to take specific actions to achieve compliance with funder and institutional open access mandates. If your research is supported by a funder that requires immediate open access (e.g. according to Plan S principles or the NIH public access policy) then you should select the gold OA route, and we will direct you to the compliant route where possible. Because authors warrant under our subscription licensing terms that they haven’t committed to licensing any version of their article under a licence inconsistent with the terms of our agreement – including the applicable embargo period – publication under the subscription model isn’t suitable for authors whose funders require no embargo.

To assist our authors in disseminating their research to the broader community, our SharedIt initiative provides you with a unique shareable link that will allow anyone (with or without a subscription) to read the published article. Recipients of the link with a subscription will also be able to download and print the PDF.

If your paper includes color figures, please be aware that in order to help cover some of the additional cost of four-color reproduction, Nature Portfolio charges our authors a fee for the printing of their color figures. Please contact our offices for exact pricing and details.

As soon as your article is published, you will receive an automated email with your shareable link.

If you have not already done so, we strongly recommend that you upload the step-by-step protocols used in this manuscript to protocols.io (https://protocols.io), an open online resource that allows researchers to share their detailed experimental know-how. All uploaded protocols are made freely available and are assigned DOIs for ease of citation. Protocols and Nature Portfolio journal papers in which they are used can be linked to one another, and this link is clearly and prominently visible in the online versions of both. Authors who performed the specific experiments can act as primary authors for the Protocol as they will be best placed to share the methodology details, but the Corresponding Author of the present research paper should be included as one of the authors. By uploading your Protocols onto protocols.io, you are enabling researchers to more readily reproduce or adapt the methodology you use, as well as increasing the visibility of your protocols and papers. You can also establish a dedicated workspace to collect your lab Protocols. Further information can be found at https://www.protocols.io/help/publish-articles.

You can use a single sign-on for all your accounts, view the status of all your manuscript submissions and reviews, access usage statistics for your published articles and download a record of your refereeing activity for the Nature Portfolio.

Nature Cell Biology encourages authors presenting evidence for cell, biological, molecular, and genetic interactions to consider communicating these findings using Biofactoid (https://biofactoid.org/). This tool helps users share a searchable representation of interactions (e.g. binding, gene expression, post-translational modification) between genes, gene products, or chemicals. Information added to Biofactoid, with author attribution, is shared on social media and public databases, such as Pathway Commons, where it can be discovered and analyzed in the context of a large and growing corpus of knowledge.

Please feel free to contact us if you have any questions.

With kind regards,

Daryl

Daryl Jason Verzosa David, PhD

Senior Editor, Nature Cell Biology
Advisory Editor, npj Biological Physics and Mechanics
Nature Portfolio

Heidelberger Platz 3, 14197 Berlin, Germany
Email: daryl.david@nature.com
ORCID: https://orcid.org/0000-0002-9253-4805

Click here if you would like to recommend Nature Cell Biology to your librarian
http://www.nature.com/subscriptions/recommend.html#forms

** Visit the Springer Nature Editorial and Publishing website at www.springernature.com/editorial-and-publishing-jobs for more information about our career opportunities. If you have any questions please click here.**

Open Access This Peer Review File is licensed under a Creative Commons Attribution 4.0 International License, which permits use, sharing, adaptation, distribution and reproduction in any medium or format, as long as you give appropriate credit to the original author(s) and the source, provide a link to the Creative Commons license, and indicate if changes were made.
In cases where reviewers are anonymous, credit should be given to 'Anonymous Referee' and the source.
The images or other third party material in this Peer Review File are included in the article’s Creative Commons license, unless indicated otherwise in a credit line to the material. If material is not included in the article’s Creative Commons license and your intended use is not permitted by statutory regulation or exceeds the permitted use, you will need to obtain permission directly from the copyright holder.
To view a copy of this license, visit https://creativecommons.org/licenses/by/4.0/

Reviewer #1 (Remarks to the Author):

In this manuscript, De Belly et al. investigate the mechanisms underlying the polarization of the GTPases Rho and Rac at opposite poles of migrating cells. Although previous studies have explored potential polarization mechanisms, this work provides new interesting insights through a combination of optical stimulation techniques, live protein biosensors and mathematical modeling. Specifically, the authors argue that the polarization of one GTPase facilitates reciprocal polarization of the other at the opposite cellular pole via a mechanism involving membrane tension-based activation of mTORC2 and cortical flow-based regulation of phosphoinositide signaling. Overall, this excellent study advances our understanding of the coordinated spatial regulation of Rho and Rac during cell migration. However, a few key issues need to be addressed prior to publication.

Major Comments:

1. In this manuscript, the authors used an opto-iSH2, which regulates PI3K/Akt. However, optogenetic activation of PI3K influences various downstream signals including Rac. Therefore, the authors should verify their key findings using a more targeted optogenetic tool for Rac, such as opto-TIAM (PMID: 37449837).

We thank the Reviewer for this idea. Accordingly, we have repeated our key experiments with opto-TIAM, and these Rac-based protrusions elicit Rho activation at the opposite end of the cell (HL60s), as observed with our opto-PI3K-induced protrusions (**Figure S1H, I**).

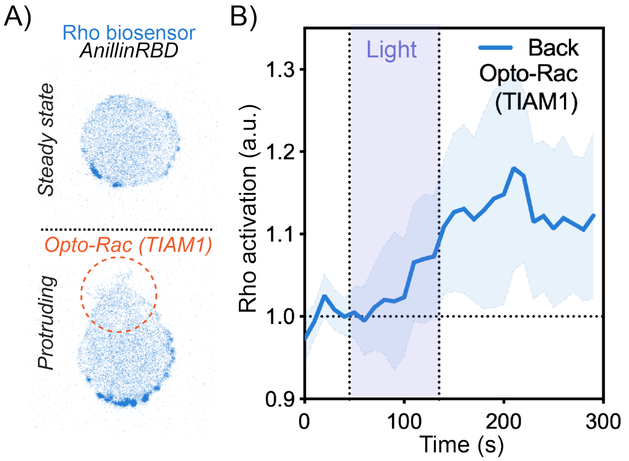

Figure S1H, I: __Direct opto-Rac activation leads to long-range Rho activation__. *A) Time-lapse confocal images of an unpolarized cell before and during opto-Rac (TIAM1) stimulation. Rho activation was monitored using the biosensor AnillinRBD. (B) Average time trace of Rho activiation at the plasma membrane for the front versus back of the cell following opto-Rac (TIAM1) activation. (mean ±95%CI; n>16, N= 2).*

More generally, our experiments suggest that neither Rac nor PI3K are directly needed for Rho activation at the opposite end of the cell, but the membrane protrusions generated by these tools elicits the membrane stretch needed to activate Rho, as can be seen by the PI3K

and Rac-independent tools we use to stretch membranes such as micropipette aspiration **(Figure 2E-G)** and osmotic-based cell swelling **(Figure 2B-D).**

2. The authors show that hypotonic shock increases Rho activity through elevated membrane tension, independently of actin-based protrusions. While mTORC is identified as a potential mediator of this response, it is worth noting that cell swelling upon hypotonic shock involves water influx through aquaporins and the ion transporter NHE1, as reported by the authors and others (PMID: 24726433). The authors should investigate whether Rho activation is also functionally linked to these volume-regulating channels.

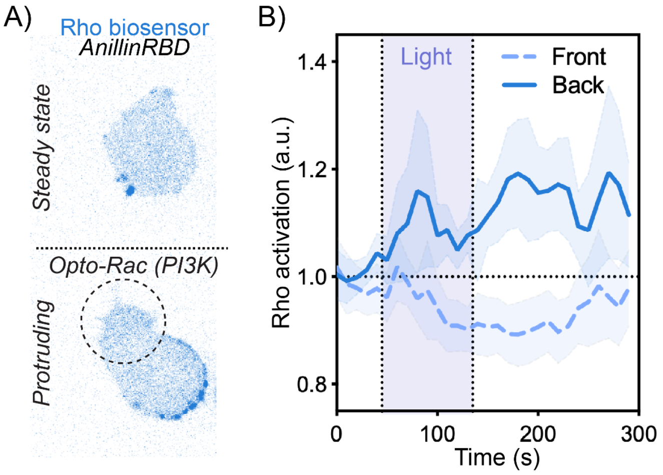

Reviewer Figure 1: <u>NHE1 inhibition does not impair the long-range Rho activation by Rac</u> *A) Time-lapse confocal images of an unpolarized cell treated with 5 μM of the NEH1 inhibitor BIX before and during opto-Rac (PI3K) stimulation. (B) Average time trace of Rho activity at the plasma membrane for the front versus back of cells treated with 5 μM of the NEH1 inhibitor following opto-Rac (PI3K) activation. (mean $\pm$95%CI; n=20, N= 2).*

We find that pharmacological inhibition of NHE1 has no effect on the ability of opto-PI3K to induce Rho-activation at the opposite end of the cell, indicating that water influx through these channels is not required for opto-Rac-induced Rho activation (**Reviewer Figure 1**).

3. According to Figure 3C, stable blebs form at the opposite end of RhoA stimulation. However, high RhoA activity also induces the formation of small blebs at the cell rear (Figure 4b). The stable blebs formed after adding Lat B show robust Rac enrichment (Fig. S5D and S6J). Do these large blebs completely lack RhoA? Also, LatB-induced blebs do not exhibit Rac enrichment following Duvelisib treatment (Fig. S6J). Are they also devoid of RhoA?

Blebs have somewhat complex dynamics of GTPase regulation. We typically observe Rac activation early in blebs, followed by Rho activation at later time points (potentially reflecting a role of Rho in retracting the blebs and maintaining cell coherence) see Reviewer Figure 3 & previous literature on the subject (PMID: 18628785; PMID: 29233866). By contrast, blebs forming close to the site of contraction typically do not show Rac activation, consistent with local Rho activation inhibiting Rac activation, even in the context of blebbing. (**Reviewer Figure 2**).

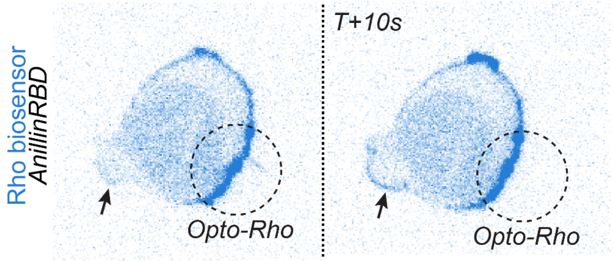

Reviewer figure 2: <u>Rho is activated in blebs.</u> *A) Time-lapse confocal images of an unpolarized opto-Rac HL-60 cell expressing the Rho biosensor (AnillinRBD) during opto-Rho stimulation.*

4. In Figure 4, the authors show that optogenetic activation of RhoA at the cell rear depletes MCA proteins at the front, releasing PIP2, which is then converted by PI3K into PIP3 to activate Rac. The authors should use a PIP3 reporter to validate this mechanism.

We thank the reviewer for this good suggestion to buttress our PI3K inhibition experiments. As expected, we observe PIP3 increases (as assayed by a PHAKT biosensor) at sites of blebbing across the cell from opto-Rho activation and at the front of migrating cells as was previously reported in Dictyostelium and macrophages (**Figure S7H-K**).

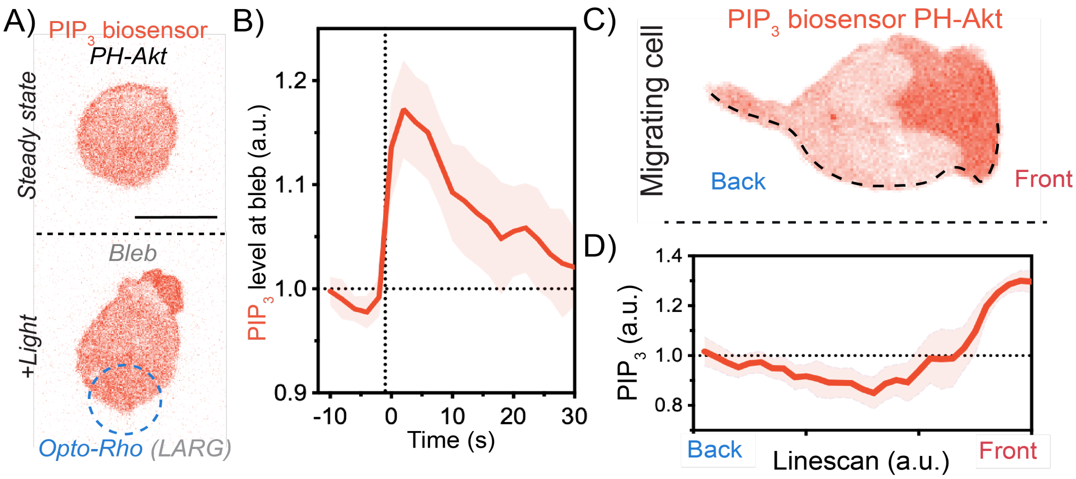

Figure S7H-K: <u>Contraction-induced blebbing leads to PIP_3 at the cell front.</u> *A) Time-lapse confocal images of an unpolarized opto-Rac HL-60 cell expressing the PIP3 biosensor (PH-Akt) at steady state and during opto-Rho stimulation. B) Average time trace of PIP3 level at the plasma membrane at the protrusive blebs, showing PIP3. Time 0 = blebbing. For times before blebbing, PIP3 activity is measured at the same membrane spot where the bleb will appear. (mean ±95%CI; n > 20, N = 2). C) Confocal image of a migrating neutrophil-like HL-60 cell expressing the PIP3 biosensor PH-Akt. D) Average line-scan of PIP3 level across migrating HL-60s, showing enrichment of PIP3 at the cell front (mean ±95%CI; n = 15, N = 2).*

5. In Figure 4D, the authors report PIP2 polarization at the cell front following opto-Rho stimulation. However, previously published work (PMID: 36202973) shows PIP2 enrichment at the rear and PIP3 localization at the front in both Dictyostelium and RAW 264.7 macrophages. How do the authors reconcile their findings with prior work?

We thank the Reviewer for this insightful suggestion, which led to an unexpected observation that is likely to be of broad interest to others in the field. For our experiments, we are using a mutated domain of tubby that is a low-affinity binder to PI(4,5)P2 and is thought to primarily recognize newly-generated/exposed PI(4,5)P2. In contrast, the higher-affinity full tubby domain can recognize total PI(4,5)P2. When we compare the distribution of these probes in migrating HL-60 cells, the low-affinity tubby domain (newly exposed PI(4,5)P2) localizes to flashes at the leading edge of the cell, whereas the high-affinity tubby domain (total PI(4,5)P2) localizes to the rear of the cell, potentially indicating unique localization of the newly-exposed versus total PI(4,5)P2 (**Figure 5D-I & Video S8, 9**).

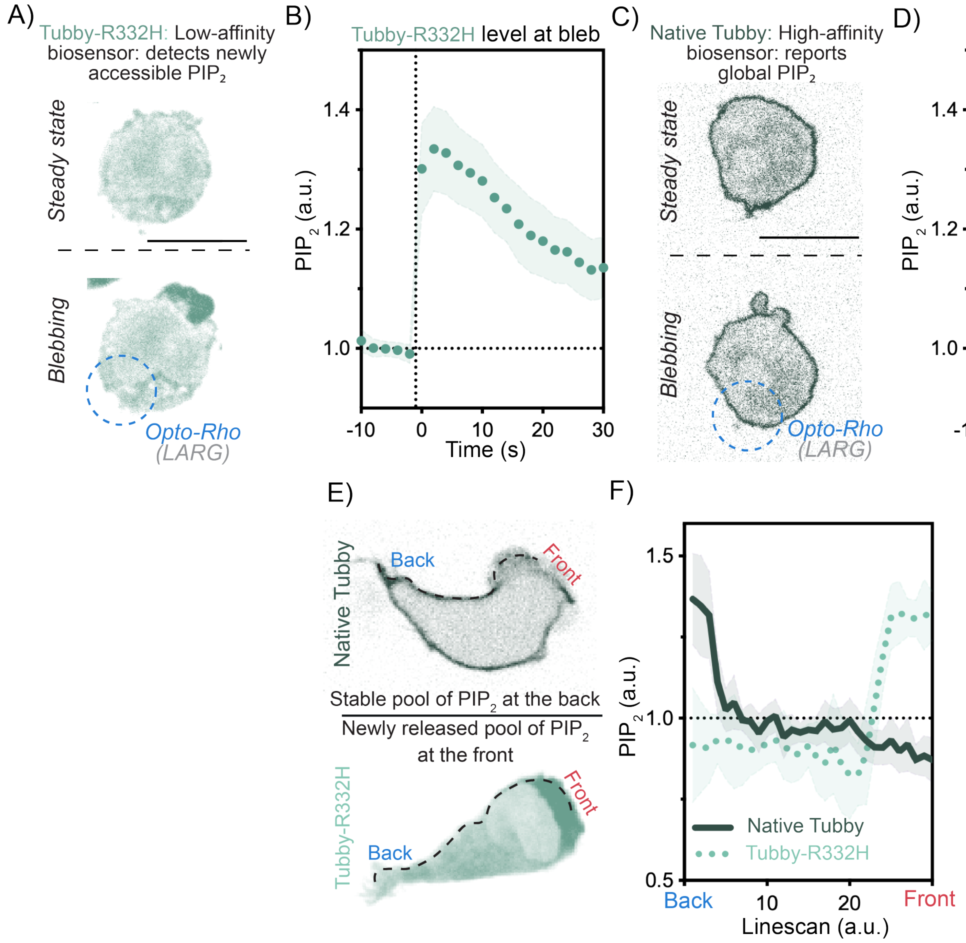

***Figure 5D-I**: **<u>Newly accessible PI(4,5)P_2 is enriched at the cell front while total PI(4,5)P_2 is enriched at the cell back.</u>** A) B) Time-lapse confocal images of an unpolarized opto-Rac HL-60 cell expressing the low-affinity PIP_2 biosensor (Tubby-R332H) before and during opto-Rho stimulation demonstrates PIP_2 enrichment in bleb-based protrusions. C) D) In contrast cells expressing a high affinity PIP_2 biosensor (Native-Tubby) which stains global PIP_2 show only a mild increase at the cell front. E) Confocal image of a migrating neutrophil-like HL-60 cell expressing either PIP2 biosensors Native Tubby or Tubby-R332H. F) Average line-scan of PIP2 level across migrating HL-60s, showing enrichment of newly accessible PIP2 at the cell front while overall level of PIP2 is higher at the cell back, as previously reported in the literature (PMID: 36202973) (mean ±95%CI; n = 10, N = 2).*

We speculated that the transient increases in PI(4,5)P2 observed at the cell front could arise from two possible mechanisms: (i) release of pre-existing PI(4,5)P2 from PI(4,5)P2-binding proteins, such as MCA proteins, or (ii) de novo synthesis of PI(4,5)P2. To distinguish between these possibilities, we treated cells either with inhibitors of PI(4,5)P2 synthesis or with the PI(4,5)P2-sequestering drug neomycin. Notably, inhibition of PI(4,5)P2 synthesis did not reduce PI(4,5)P2 levels at the cell front, whereas neomycin treatment caused a pronounced decrease in PI(4,5)P2 at the front (**Figure S7B-F**). These results support the release-based mechanism as the more likely origin of the transient PI(4,5)P2 increase. In future work, we aim to directly test this model by manipulating local PI(4,5)P2 availability with more targeted approaches (e.g. optogenetic control). We anticipate that this may require perturbations beyond PI5K translocation —such as targeted MCA release— and is therefore beyond the scope of the current manuscript.

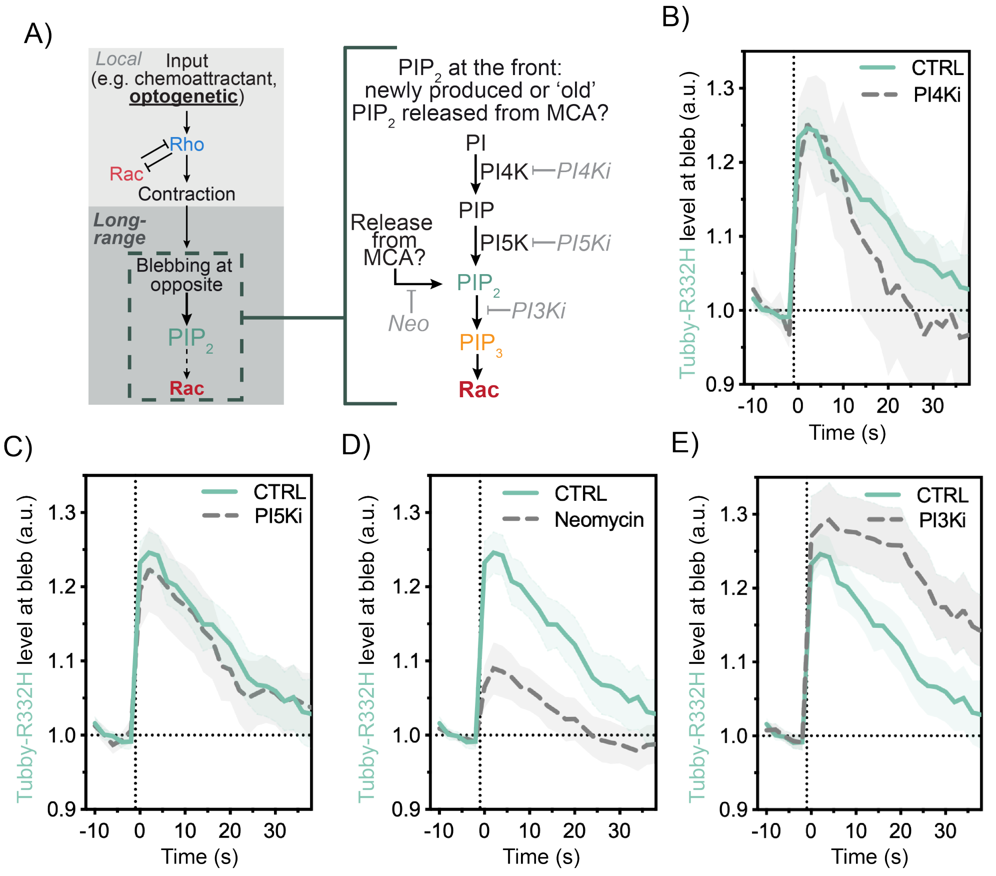

Figure S7B-F: <u>PI(4,5)P2 Synthesis Inhibition Fails to Suppress Front Enrichment, Whereas Sequestration Abolishes It</u>. *Average time traces of PI(4,5)P2 levels (assayed with Tubby-R332H) at the plasma membrane at the protrusive blebs in control and cells treated with either 200 nM of PI4K inhibitor (GSK-A1), 5 µM of PI5K inhibitor (PIP5K1C-IN-1), 10mM of Neomycin or 1 µM PI3Kδ/γ inhibitor Duvelisib (mean ±95%CI; n>30, N= 3).*

6. Based on the MCA-PIP2-Rac model, how do the authors explain the data reported in Figures 4C and 4F, where ezrin remains localized at the rear for over 300 seconds after stimulation, while the increase in Rac activity lasts less than 30 seconds?

We hypothesize that Rac is not responding to the absolute concentration of MCA proteins at a given region, but depletion of MCA increases the probability of blebs (**Figure S6C**), and the act of blebbing is what generates the PIP2 increases and Rac activation (so small changes in MCA have the potential to induce large changes in PIP2 and Rac if they trigger blebs). Wholescale redistribution of Ezrin takes many minutes, but it can start to be depleted much more rapidly.

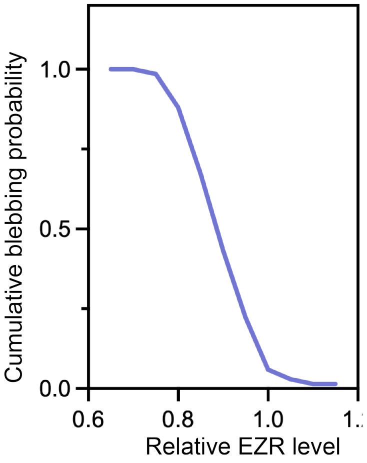

***Figure S6C**: __Depletion of MCA increases the probability of blebs.__ Cumulative blebbing probability in function of relative Ezrin level. (N = 3, n >40).*

It is also important to note that we are using Ezrin as a possible readout of MCA. However, tagged Ezrin does not allow us to distinguish between phosphorylated (engaging with membrane and cortex) and non phosphorylated Ezrin. In addition, we know that there are many other proteins that mediate MCA in these cells, and other proteins may display a different dynamic of redistribution. To address this, we used another approach to visualize close interactions of the cortex and membrane by using MPAct (PMID: 32527825). This tool confirmed that local contraction enriches MCA at the contraction site while depleting it from the opposite side of the cell (**Figure S6D-F**).

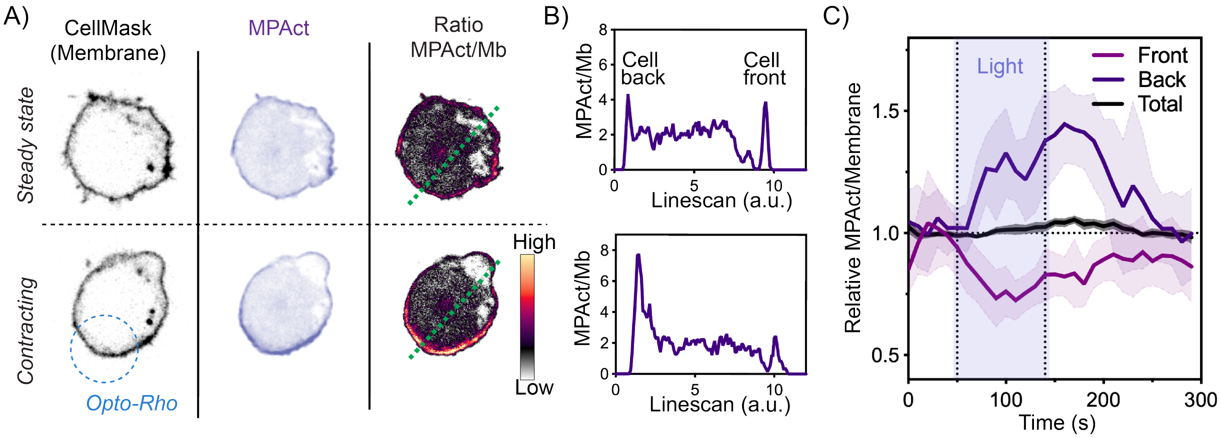

***Figure S6D-F**: __Local contraction enriches MCA at the back while depleting it at the front.__ A) Time-lapse confocal images of unpolarized cells expressing the MPact, a membrane-actin proximal sensor, and stained with the membrane marker CellMask before and during opto-Rho stimulation. The ratio between MPact and membrane channel is used to measure the approximative MCA level intensity. EB) Line-scan of MPAct/Membrane ratio of cell displayed in A), see green dotted line. C) Average time trace of relative MPAct/Membrane ratio (normalized to before light) at the front and back of the cell. (N = 2, n >10, means ± 95%CI).*

7. In Figure 5, the authors aim to extend their mechanism to non-migratory cells. To validate the Rho-Blebbing-PIP2-Rac axis cells, they should examine Rac activity instead of only PIP2 levels. a. In Figure 5, they should consider using opto-Rac and opto-Rho in one cell type, rather than relying solely on hypotonic shock and LatB treatment.

We now perform our Opto-Rho in multiple cell types (Hela, 293Ts and MDCKs) and read out Rac activation via Pak-PBD and find Rac enrichment in blebs, consistent with our observations in HL60s (**Figure 6I, J & S8**).

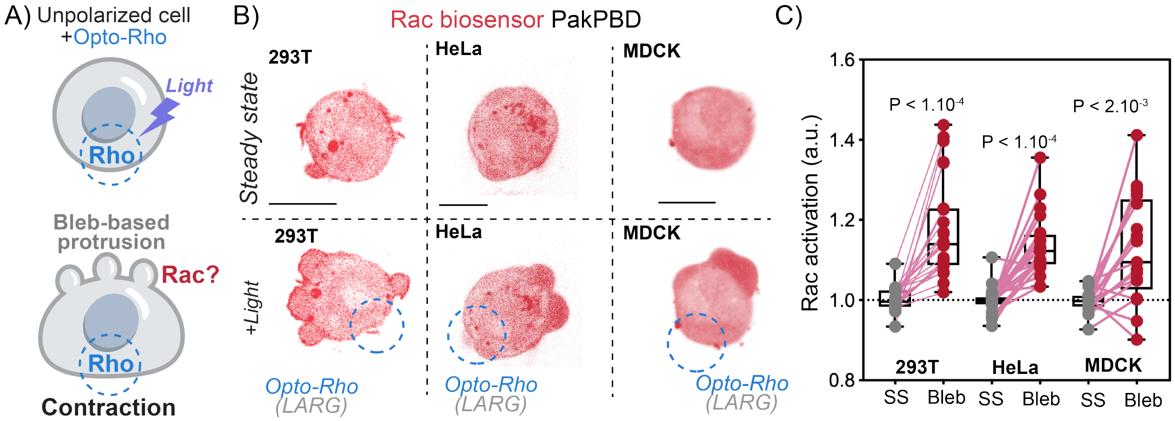

***Figure 6I, J & S8**: <u>The long-range activation of Rac by Rho is conserved in non-migratory cells.</u> A) B) Time-lapse confocal images of an unpolarized opto-Rac 293T, HeLa and MDCK cell expressing the Rac biosensor (Pak-PBD) at steady state and during opto-Rho stimulation. Local Rho activation induces blebs at the opposite end of the cell, and these blebs are enriched in Rac. C) Relative Rac level at the plasma membrane at steady state and at protrusive blebs, showing Rac enrichment in blebs. Box and whiskers: median and min to max; p values from Wilcoxon paired Student's t test.*

Opto-Rac was not sufficiently potent to drive robust protrusions in epithelial cells (it did function in HL-60 cells, see **Figure S1H, I**), so we used micropipette aspiration to generate membrane deformations directly and assayed the resulting Rho activation (via Anillin-RBD) in 293T, HeLa, and MDCK cells (**Figure 6D, E & S8**).

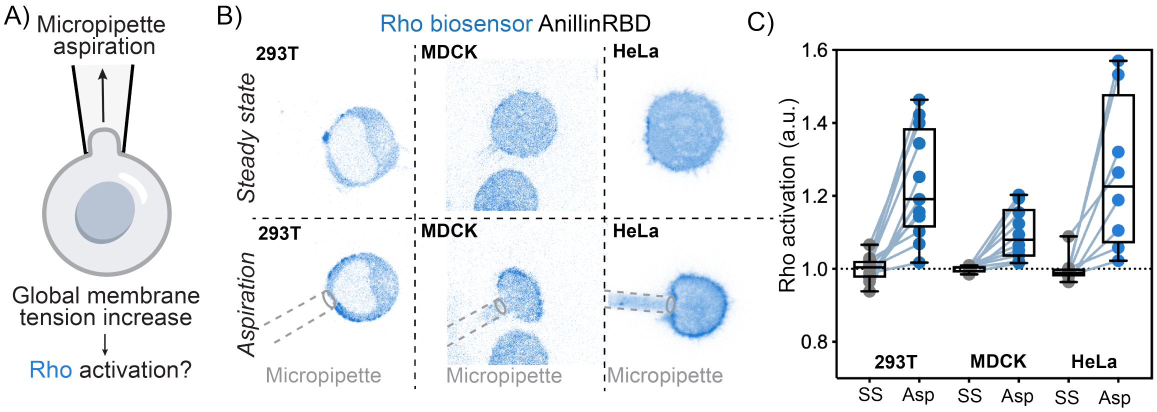

***Figure 6D, E & S8**: <u>The membrane tension dependent activation of Rho is conserved in non-migratory cells.</u> A) B) Time-lapse confocal images of 293T, MDCK and HeLa cells expressing the Rho biosensor (AnillinRBD) before and after micropipette aspiration. (B) Average Rho activity before (steady state) and during aspiration. Box and whiskers: median and min to max; p values from Wilcoxon paired Student's t test.*

8. The authors should validate some of the important experiments using anillin-RBD with a RhoA-2xRBD-tdTomato sensor (PMID: 34357388).

We tested the RhoA-2xRBD-tdTomato sensor, but it did not give sufficient signal-to-noise in our hands (we tested it in both HL60s and HEK293Ts). As a more direct readout of the functional cytoskeletal signaling downstream of Rho activation, we analyzed the dynamics of a fluorescently-tagged myosin regulatory light chain and found it to parallel the increases in Rho activation, as assayed by our Anillin sensor following opto-PI3K activation at the other end of the cell (**Figure S1J, K**).

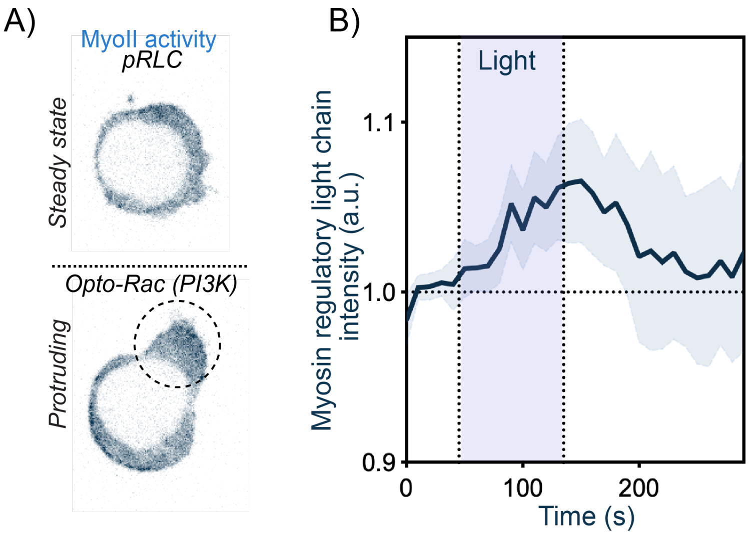

Figure S1J, K: <u>Local Rac activation leads to myosin activation at the opposite side of the cell.</u> *A) Time-lapse confocal images of an unpolarized cell. Myosin II activity was monitored using the biosensor pRLC. (B) Average time trace of pRLC level at the plasma membrane for the back of cells following opto-Rac (PI3K) activation. (mean ±95%CI; n=15, N= 2).*

9. The authors should consider using a fluorophore lifetime-based membrane tension sensor (PMID: 36323783) to validate their claims in the paper.

In our hands, the Flipper-based membrane tension sensors impair cell motility at the doses needed for visualization. We've had much better luck with the direct optical-trap-based (or AFM-based) readouts of membrane tension (PMID: 27280401, 3731145 & : 2226541)

Reviewer #2 (Remarks to the Author):

This study by De Belly and colleagues addresses an important unanswered question in the regulation of cellular polarisation: how the GTPases RAC and RHO regulate each other from spatially distinct regions of the cell. They report a mechanism by which membrane tension caused by RAC-induced membrane projections causes the activation of RHO on the membrane at the opposite side of the cell, an effect they report to be dependent on MTORC2 signalling. They also report an inverse regulation where RAC is activated at the leading edge in response to RHO activation at the rear of the cell and report that this is due to increases in PIP¬2 concentrations leading to a PI3K dependent activation of RAC. The authors then demonstrate the presence of these reported mechanisms in epithelial cells and

primary T-cells.The work is explained well (there are some great visual aids throughout the figures and well-annotated legends) and understanding the long-range regulation of these GTPases by each other is important for research into normal development and multiple diseases including immune deficiencies and cancer. However, I have concerns about the mechanisms they report, and the tools used in this work. My specific concerns are detailed below:

1. The primary tools used in this work are fluorescent protein-tagged binding domains that bind to the active form of specific GTPases. It is, however, unclear which GTPases they are referring to in this paper as the specificity of these probes is broader than indicated. For example, the Pak Binding Domain (PBD) will bind to RAC1, RAC2, RAC3 and CDC42. However, the authors only refer to "RAC" without specifying which RAC family member. This is important because RAC1 and 2 are both expressed and have distinct roles in immune cells. The authors therefore need to demonstrate which RAC family member is performing the reported function and be specific in the manuscript, that is to say replace "RAC" with reference to a specific RAC family member. They also need to discount the role of CDC42 as the probe will also be binding CDC42-GTP. Likewise, simply writing "RHO" is not informative; are the authors referring to RHOA? Does this probe bind any other RHO family members? As the work is centred on the use of these probes, it is essential that their specificity is confirmed and the specific proteins performing the functions identified referred to throughout.
We thank the Reviewer for raising this point and we have revised the manuscript to be explicit about what each biosensor can and cannot report. For Rac activation, we hypothesize that both Rac1 and Rac2 play a role, as both are activated downstream of PI3K and Tiam, both are required for protrusion generation in neutrophils, and both suffice to induce cell protrusions. We modified the text accordingly to explain this point at the outset. In addition to leveraging opto-PI3K to generate protrusions, we now also make use of opto-TIAM, which activates both Rac1 and Rac2 (**Figure S1H, I**).

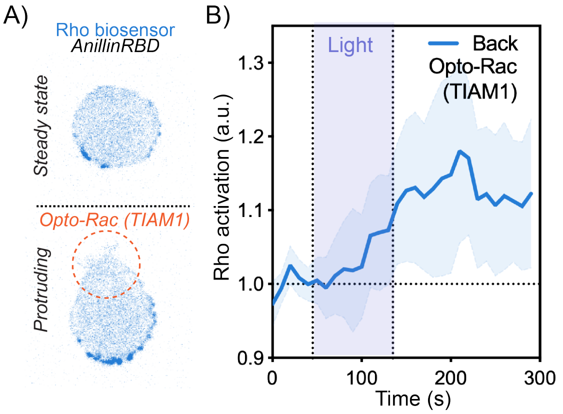

***Figure S1H, I*: *<u>Direct opto-Rac activation leads to long-range Rho activation</u>*.** *A) Time-lapse confocal images of an unpolarized cell before and during opto-Rac (TIAM1) stimulation. Rho activity was monitored using the biosensor AnillinRBD. (B) Average time trace of Rho activity at the plasma membrane for the front versus back of the cell following opto-Rac (TIAM1) activation. (mean ±95%CI; n>16, N= 2).*

To address potential Cdc42 contributions to Pak-PBD recruitment, we used the well characterized Cdc42 inhibitor ML141 and found that it does not affect Opto-LARG-driven Pak-PBD recruitment at the opposite end of the cell (**Figure S5B**), arguing that our Pak-PBD signal in this context is not dominated by Cdc42-GTP.

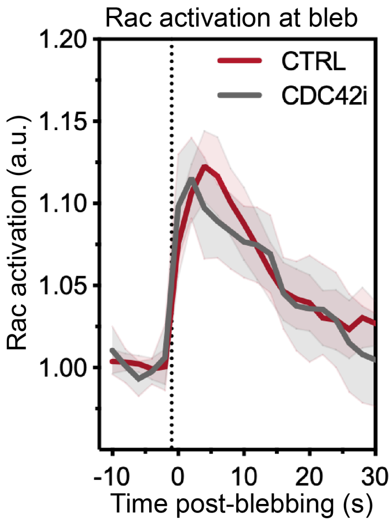

***Figure S5B**: __CDC42 inhibition fails to impair PBD recruitment in blebs.__* *Average time trace of Rac activity measured by Pak-PBD at the plasma membrane of control cells or cells treated with 10 micromolar of the CDC42 inhibitor ML141following opto-Rho (LARG) activation. (mean ±95%CI; n>20, N= 2).*

Anillin RBD primarily binds to active RhoA (PMCID: PMC4449299, PMID: 18158243), we thus use it as purported activation readout of RhoA. We have edited the text to make this clarification. As an additional means to assay Rho via its downstream effector, we analyzed the dynamics of a fluorescently-tagged myosin regulatory light chain and found it to closely parallel the increases in Rho activation as assayed by our Anillin sensor (**Figure S1J, K**) following opto-PI3K activation at the other end of the cell.

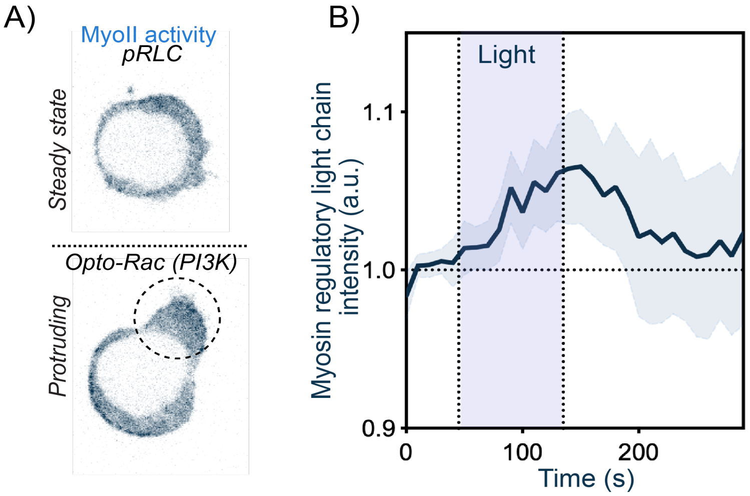

***Figure S1J, K**: __Local Rac activation leads to myosin activation at the opposite side of the cell.__* *A) Time-lapse confocal images of an unpolarized cell. Myosin II activity was monitored using the biosensor pRLC. (B) Average time trace of pRLC level at the plasma membrane for the back of cells following opto-Rac (PI3K) activation. (mean ± 95%CI; n=15, N= 2).*

2. The mechanism the authors show in figure 2 for membrane tension activating RHO in a RICTOR-dependent (mTORC2) manner needs further elaboration. MTOR signalling to RHOA has been reported before, it is therefore important to deduce whether this function of MTOR occurs throughout the cell, the periphery of the cell (locally activating RHO) or alternatively at the site of membrane protrusion.

We thank the Reviewer for this suggestion. As an additional way to show mTORC2 involvement beyond genetic and pharmacological inhibition, we potentiated mTORC2

activation with insulin and found that this enhances Rho activation following membrane stretch (**Figure S4E-G**).

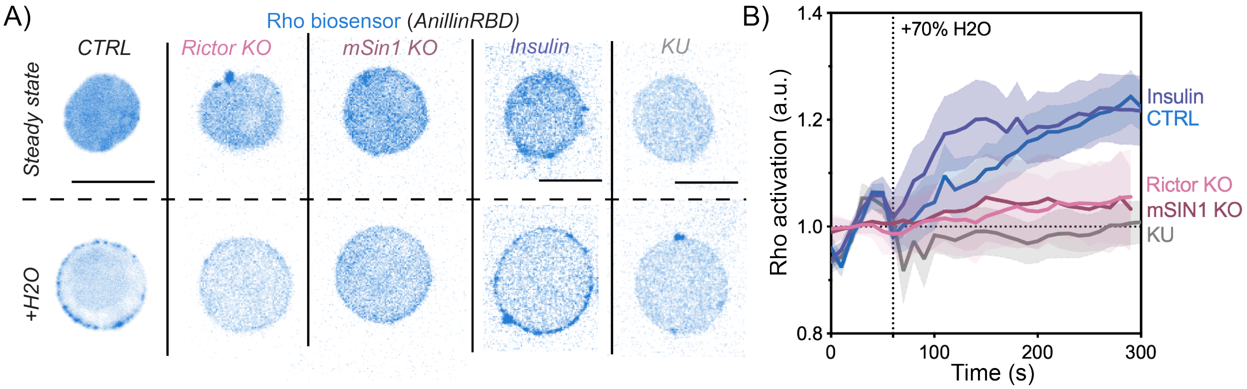

***Figure S4E-G*: <u>Potentiation of mTORC2 by Insulin increases Rho activity following membrane stretch by osmotic shock.</u>** *A) Time-lapse confocal images of unpolarized control, Rictor KO, mSIN1 KO cells or cells treated with 10uM of the mTOR inhibitor KU-0063794, or with Insulin (10ug/ml) expressing AnillinRBD-mCherry before and during hypotonic shock (70% Water). (B) Average time trace of Rho activity at the plasma membrane before and after hypo-osmotic shock in control demonstrate a requirement for mTORC2 in membrane-tension mediated activation of RhoA (mean ±95%CI; n>35, N= 3).*

We were initially hesitant to analyze mTORC2 dynamics given the difficulty of doing so in the *Dictyostelium* field where even fluorescently-tagged components that rescue the knockouts not showing obvious localization. However, based on work performed in HeLa cells (PMID: 28143890), we successfully tagged the mTORC2-specific subunit mSIN1 to analyze mTORC2 dynamics in HL60 cells and found it to localize to the leading edge of migrating cells, to the site of protrusions in opto-PI3K-simulated cells, and at the plasma membrane in osmotically swollen cells (**Figure 3E-H & S4K-N**)

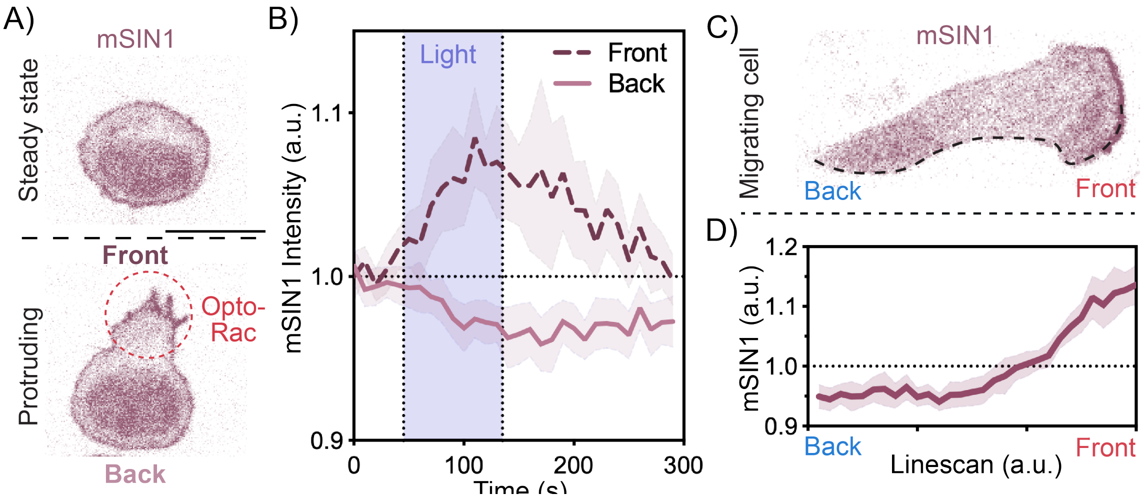

***Figure 3E-H & S4K-N*: <u>The mTORC2 Subunit mSIN1 Localizes at the cell front.</u>** *A) Time-lapse confocal images of an unpolarized cell before and during opto-Rac stimulation. mTORC2 location was monitored via mSIN1. B) Average time trace of mSIN1 level at the plasma membrane at the site of opto-Rac activation (cell front) compared to the opposite side of the cell (cell back). (mean ±95%CI; n=20, N= 3). C) Confocal image of a migrating neutrophil-like HL-60 cell expressing mSIN1. D) Average line-scan of mSIN1 level across migrating HL-60s, showing enrichment at the cell front (mean ±95%CI; n = 13, N = 2).*

This is a very interesting result and could suggest that mTORC2 is responding to the protrusion-induced increases in membrane tension. If these increases in mTORC2 localization reflect an increase in activity, it would suggest that soluble effectors must be needed to link the increases in mTORC2 activity at protrusions to Rho activation at the other end of the cell; there is a precedent for this sort of cross-cell communication in Dictyostelium (PMID: 10545500 & 11389841). We now expand on this fascinating new results in the discussion and hope to explore it further in future work.

3. The authors report that RHO activation leads to blebbing at the opposite side of the cell to cause RAC activation. They identify a mechanism where Membrane-to-Cortex-Attachment proteins dissociate from the membrane and move to the periphery causing blebbing and a local increase in PIP2 at the front of the cell, leading in turn to PI3K activity and RAC activation. This mechanism is not convincing. The loss of EZR mCherry at the leading edge occurs on a longer time scale than the generation of PIP2, which is very rapid (fig 4B-E). This implies that the PIP2 increase is occurring prior to the loss of EZR at this site.

Reviewer 1 had a similar concern. We hypothesize that Rac is not responding to the absolute concentration of MCA proteins at a given region, but depletion of MCA increases the probability of blebs (see **Figure S6C**), and the act of blebbing is what generates the PIP2 increases and Rac activation (so small changes in MCA have the potential to induce large changes in PIP2 and Rac if they trigger blebs). Wholescale redistribution of Ezrin takes many minutes, but it can start to be depleted much more rapidly.

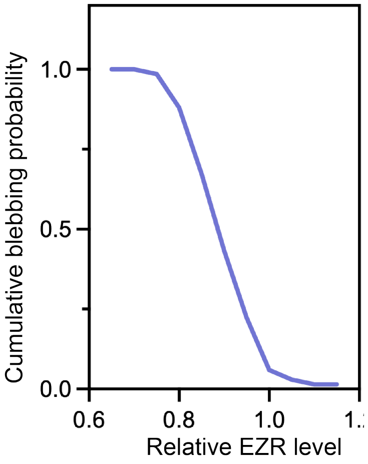

***Figure S6C**: __Depletion of MCA increases the probability of blebs.__ Cumulative blebbing probability in function of relative Ezrin level. (N = 3, n >40).*

It is also important to note that we are using Ezrin as a possible readout of MCA, but we know that there are many other proteins that mediate MCA in these cells, and other proteins may display a different dynamics of redistribution. To address this, we used another approach to visualize close interactions of the cortex and membrane by using MPAct (PMID: 32527825).This tool confirmed that local contraction enriches MCA at the contraction site while depleting it from the opposite side of the cell (**Figure S6D-F**).

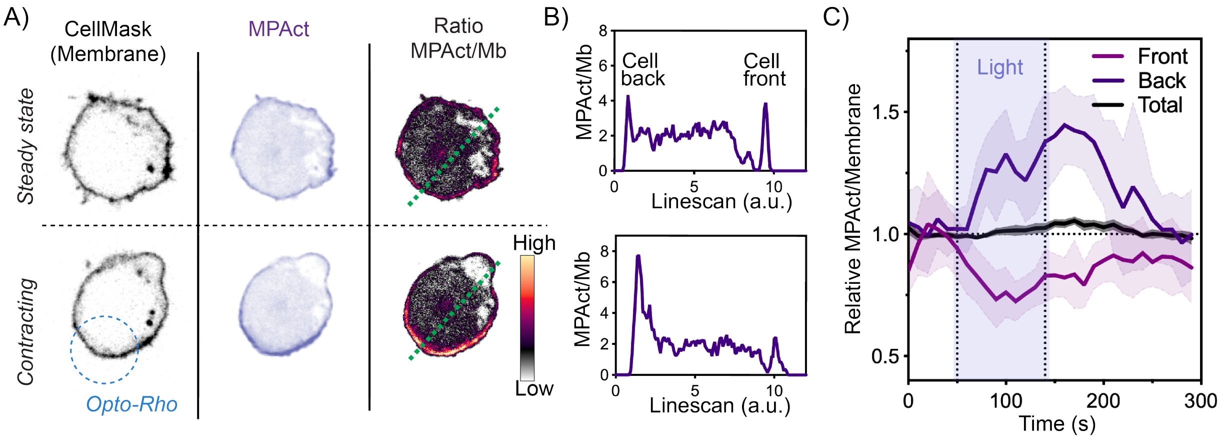

Figure S6D-F: ***Local contraction enriches MCA at the back while depleting it at the front.*** *A) Time-lapse confocal images of unpolarized cells expressing the MPact, a membrane-actin proximal sensor, and stained with the membrane marker CellMask before and during opto-Rho stimulation. The ratio between MPact and membrane channel is used to measure the approximative MCA level intensity. EB) Line-scan of MPAct/Membrane ratio of cell displayed in A), see green dotted line. C) Average time trace of relative MPAct/Membrane ratio (normalized to before light) at the front and back of the cell. (N = 2, n >10, means ± 95%CI).*

The connection to PI3K and RAC activation is also not robust. The inference that PI3K is activated by an excess of PIP2 is not supported by data and not a recognised mechanism of PI3K activation. To support this, the authors need to demonstrate that a local increase in PIP2 in this system is required and sufficient to activate PI3K, leading to a localised increase in PIP3 and subsequent RAC activation.

We agree with the Reviewer that our wording should be more careful. We do not mean to imply that an "excess of PIP2" is a non-canonical activator of PI3K. Rather, our interpretation is that a locally accessible pool of PI(4,5)P2 can be rate-limiting as the substrate of PI3K, such that transient increases in available/newly exposed PI(4,5)P2 enable robust, localized PI(3,4,5)P3 production once PI3K is engaged by canonical inputs. This idea is consistent with prior work showing that perturbing PI(4,5)P2 synthesis can disproportionately reduce PI(3,4,5)P3/Akt signaling, consistent with locally organized PI(4,5)P2 pools feeding PI(3,4,5)P3 generation (PMID: 25617736), and with precedent that increased PI(4,5)P2 can enhance PI(3,4,5)P3 production (PMID: 31616009). We quote below PMID: 2517736: "In this particular study, knockdown of PIPKIα reduces approximately 40% of global PI4,5P2 levels in keratinocytes, but the reduction of PI3,4,5P3 levels and Akt activation is much greater (up to approximately 90% reduction) suggesting that PI4,5P2 and PI3,4,5P3 synthesis might be locally organized and that only a specific pool of PI4,5P2 (but not all) is available and responsible for PI3,4,5P3 generation."

To specifically address the Reviewer's "required vs sufficient" request in our system, we added experiments and revised the text to focus on accessibility of PI(4,5)P2. We distinguish "newly exposed" versus "total" PI(4,5)P2 using low- vs high-affinity Tubby probes: the low affinity probe reports leading-edge flashes, whereas the high-affinity probe reports rear-enriched bulk PI(4,5)P2 (**Figure 5D-I & Video S8, 9**), supporting the notion that distinct accessible pool is present at the front.

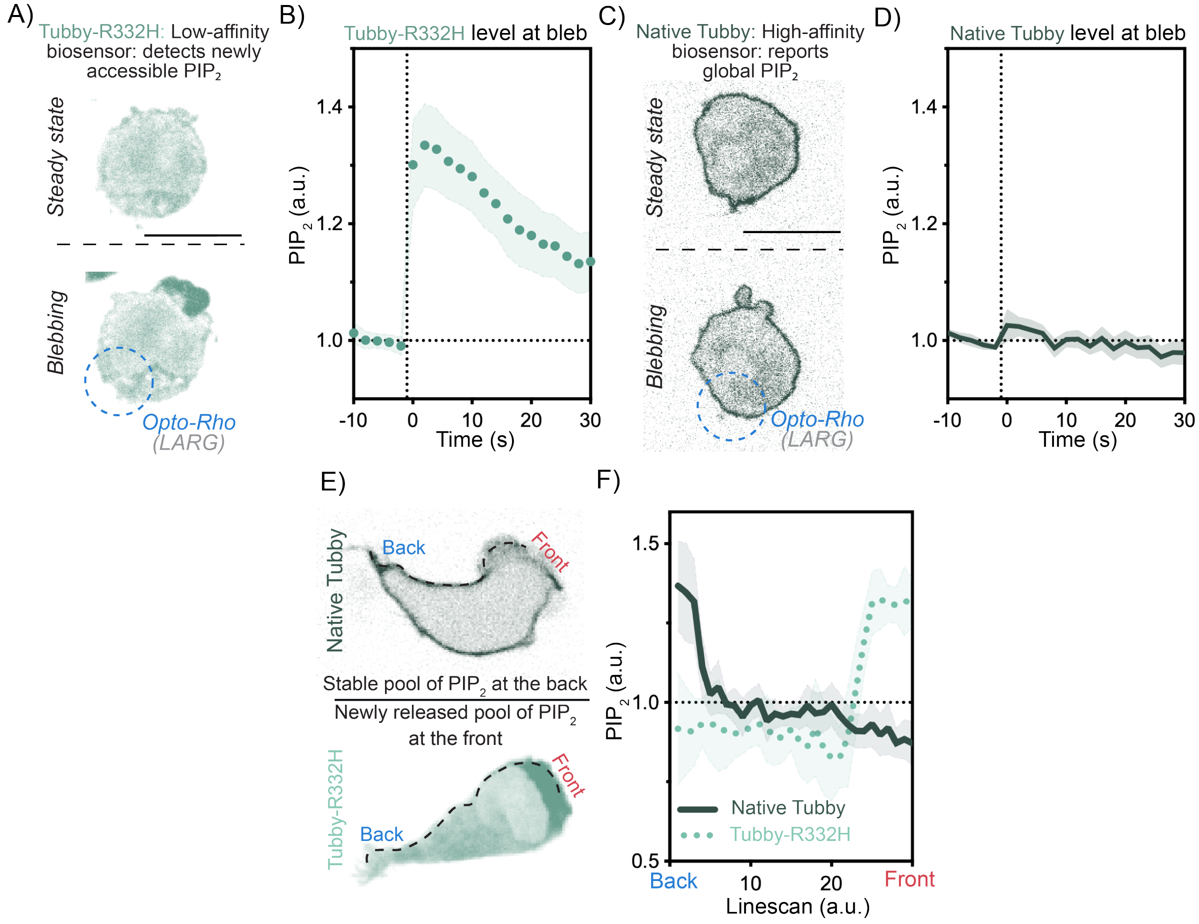

***Figure 5D-I**: **<u>Newly accessible $PI(4,5)P_2$ is enriched at the cell front while total $PI(4,5)P_2$ is enriched at the cell back.</u>** A) B) Time-lapse confocal images of an unpolarized opto-Rac HL-60 cell expressing the low-affinity PIP_2 biosensor (Tubby-R332H) before and during opto-Rho stimulation demonstrates PIP_2 enrichment in bleb-based protrusions. C) D) In contrast cells expressing a high affinity PIP_2 biosensor (Native-Tubby) which stains global PIP_2 show only a mild increase at the cell front. E) Confocal image of a migrating neutrophil-like HL-60 cell expressing either PIP2 biosensors Native Tubby or Tubby-R332H. F) Average line-scan of PIP2 level across migrating HL-60s, showing enrichment of newly accessible PIP2 at the cell front while overall level of PIP2 remain higher at the cell back as previously reported in the literature (mean ±95%CI; n = 15, N = 2).*

We speculated that the transient increases in PI(4,5)P2 observed at the cell front could arise from two possible mechanisms: release of pre-existing PI(4,5)P2 from PI(4,5)P2-binding proteins, such as MCA proteins, or de novo synthesis of PI(4,5)P2. To distinguish between

these possibilities, we treated cells either with inhibitors of PI(4,5)P2 synthesis or with the PI(4,5)P2-sequestering drug neomycin. Notably, inhibition of PI(4,5)P2 synthesis did not reduce PI(4,5)P2 levels at the cell front, whereas neomycin treatment caused a pronounced decrease in PI(4,5)P2 at the front (**Figure S7B-F**). These results support the release-based mechanism as the more likely origin of the transient PI(4,5)P2 increase. In future work, we aim to directly manipulate local PI(4,5)P2 levels; however, this will likely require more sophisticated perturbations than PI5K translocation, such as targeted MCA release, and is therefore beyond the scope of the current manuscript.

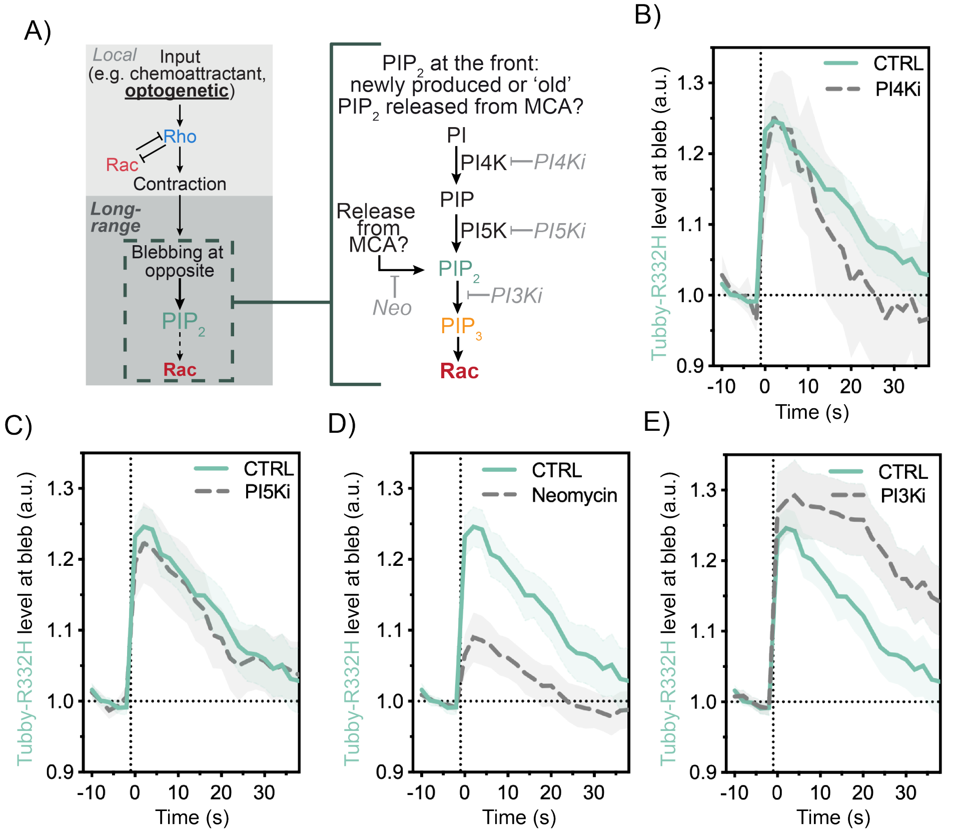

<u>Figure S7B-F</u>: <u>PI(4,5)P2 Synthesis Inhibition Fails to Suppress Front Enrichment, Whereas Sequestration Abolishes It</u> *Average time traces of PI(4,5)P2 levels (assayed with Tubby-R332H) at the plasma membrane at the protrusive blebs in control and cells treated with either 200 nM of PI4K inhibitor (GSK-A1), 5 μM of PI5K inhibitor (PIP5K1C-IN-1), 10mM of Neomycin or 1 μM PI3Kδ/γ inhibitor Duvelisib (mean ± 95%CI; n>30, N= 3).*

3b. The cause and effect of this mechanism is also not clear. For example, it is conceivable that an intracellular signalling cascade caused by RHOA activation results in the PI3K dependent activation of RAC1 on the opposite side of the cell which subsequently triggers blebbing and relocalisation of EZR. In my mind this is the most likely mechanism of action and needs to be investigated by the authors.

We agree with the Reviewer that epistasis could, in principle, involve multiple feedback loops, and we considered the alternative ordering proposed by the Reviewer (RhoA -

>PI3K/Rac->blebbing->ERM/Ezrin relocalization). However, when we treat the cells with PI3K inhibitors, we do not observe any changes in opto-Rho-mediated ERM redistribution, supporting our proposed model of MCA redistribution. (**Reviewer Figure 3**).

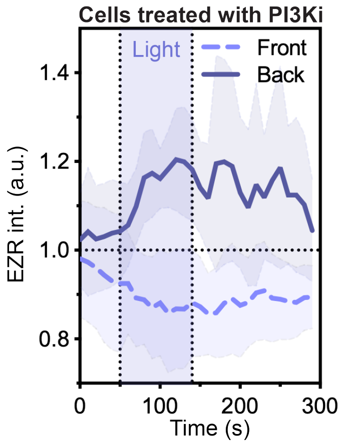

Reviewer Figure 3: <u>Inhibition of PI3K does not impair contraction induced MCA asymmetry.</u> *Average time trace of relative Ezrin intensity at the back (purple) and front (grey) of cell treated with 1 μM PI3Kδ/γ inhibitor Duvelisib in response to opto-Rho activation (at the back). (N = 2, n >20, means ± 95%CI).*

4. The authors seek to demonstrate the identified mechanisms in epithelial cells. However, there are some problems with this approach. Firstly, the origin of HEK293 cells is debatable and considered by many not to be epithelial. Moreover, neither HEK293 or HeLa cells polarise well in culture. Therefore, these are not appropriate cells to study the polarisation of epithelial cells. Furthermore, the approach used, where cells are in suspension, has problems due to the induction of anoikis in epithelial cells. HeLa cells are more resistant to anoikis than primary epithelial cells, yet they still exhibit some sensitivity. When applying this approach to an appropriate epithelial cell line anoikis will be a prominent issue. It is notable that RHOA/ROCK signalling is known to have roles in anoikis and inhibition of this pathway is often used to prevent it. Therefore, the authors need to demonstrate that the identified mechanisms occur in adherent epithelial cells and that these mechanisms are required for the maintenance of epithelial cell polarisation.

We thank the Reviewer for raising concerns regarding cell identity, epithelial polarization, and potential cofounding effects of suspension/anoikis. Our use of transient suspension assays was not intended to model epithelial polarization per se, but rather to isolate and mechanistically probe the coupling between RhoA-driven cortical contractility, bleb formation, and localized Rac signaling in a geometry where cells are unpolarized and cortical dynamics can be readily visualized and optogenetically manipulated. Importantly, these experiments were performed on short timescales (minutes), well before overt anoikis-associated phenotypes such as caspase activation or loss of cortical integrity become apparent.

We also agree that HEK293 and HeLa cells are suboptimal models for studying epithelial polarization. To directly address this concern, we now extend our optogenetic and mechanical perturbations to MDCK cells, which polarize robustly in culture. Using opto-Rho

together with the same Rac activation reporter Pak-PBD, we observed Rac enrichment in blebs in MDCK cells, consistent with our findings in HL60s (**Figure 6I, J & S8**:).

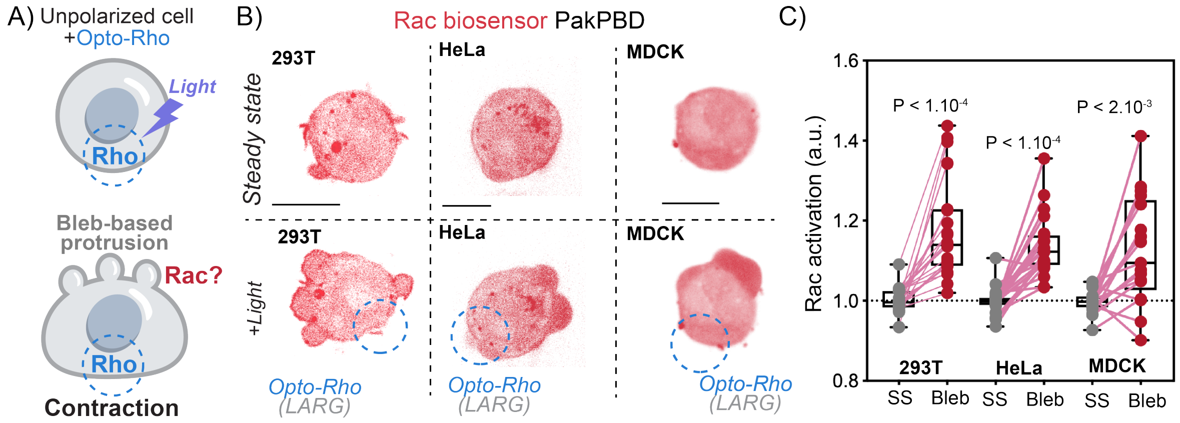

***Figure 6I, J & S8**: <u>**The long-range activation of Rac by Rho is conserved in non-migratory cells.**</u> A) B) Time-lapse confocal images of an unpolarized opto-Rac 293T, HeLa and MDCK cell expressing the Rac biosensor (Pak-PBD) at steady state and during opto-Rho stimulation. Local Rho activation induces blebs at the opposite end of the cell, and these blebs are enriched in Rac. C) Relative Rac level at the plasma membrane at steady state and at protrusive blebs, showing Rac enrichment in blebs. Box and whiskers: median and min to max; p values from Wilcoxon paired Student's t test.*

We also used micropipette aspiration to generate membrane deformations directly and assayed the resulting Rho activation (via Anillin-RBD) in 293T, HeLa, and MDCK cells (**Figure 6D, E & S8**).

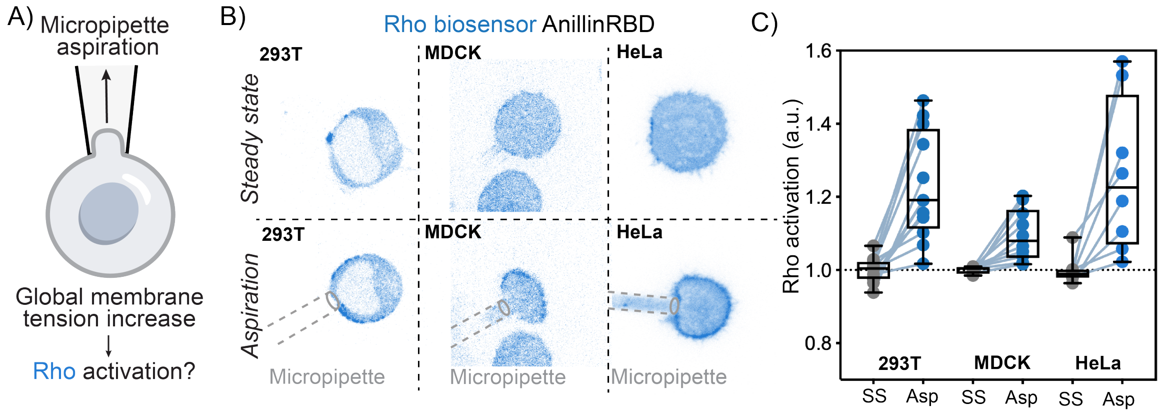

***Figure 6D, E & S8**: <u>**The membrane tension dependent activation of Rho is conserved in non-migratory cells.**</u> A) B) Time-lapse confocal images of 293T, MDCK and HeLa cells expressing the Rho biosensor (AnillinRBD) before and after micropipette aspiration. (B) Average Rho activity before (steady state) and during aspiration. Box and whiskers: median and min to max; p values from Wilcoxon paired Student's t test.*

5. The authors then suggest that these mechanisms occur in primary T-cells and show that the inhibition of Gαi or actomyosin can inhibit T-cell migration. With regard to this, it is not

novel that inhibiting Gαi signalling or actomyosin prevents migration and these experiments do not exclusively support the reported mechanisms functioning in T-cells.

We agree with the Reviewer that it is not, by itself, novel that inhibition of Gαi or actomyosin contractility impairs T cell motility. What was unexpected in our hands, is that Gαi was dispensable for T-cell polarization following chemotactic stimulation, even though it is important for efficient motility. This suggests that a substantial component of the polarization program can be driven by the trailing edge signaling module and motivates our proposal that rear-to-front crosstalk–as defined by our neutrophil experiments also contributes to T cell polarity and chemotaxis.

To expand on our T cell experiments, we extend our analysis to RhoA activation and found that CCL19 induces, as expected, long-lasting Rho polarization (**Figure S10A-C**). In contrast, impairing mTORC2 activity using a mTOR inhibitor elicits only transient Rho polarization and a surprisingly robust impairment of primary T-cell migration (**Figure 8H, I, O, S10C, S11E**).

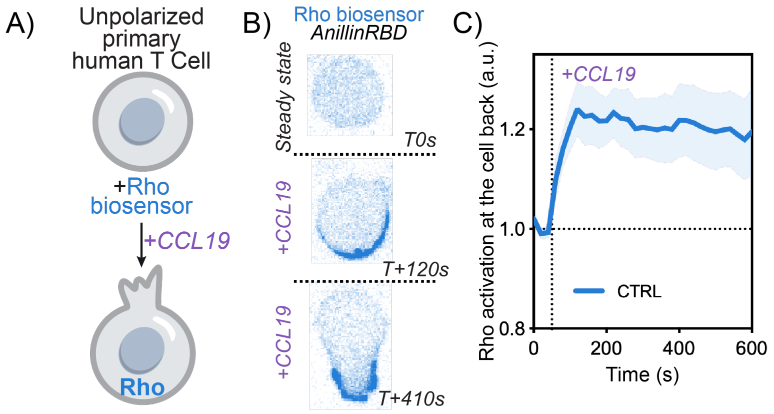

***Figure S10A-C*: <u>Chemoattractant CCL19 induces robustlong-lasting Rho polarization.</u>** *A) B) Time-lapse confocal images of an unpolarized human primary T cell before and during CCL19 stimulation. Rho activation was monitored via the Rho biosensor Anillin-RBD. (C) Average time trace of Rho activation at the cell back control cells (mean ±95%CI; n>20, N = 3).*

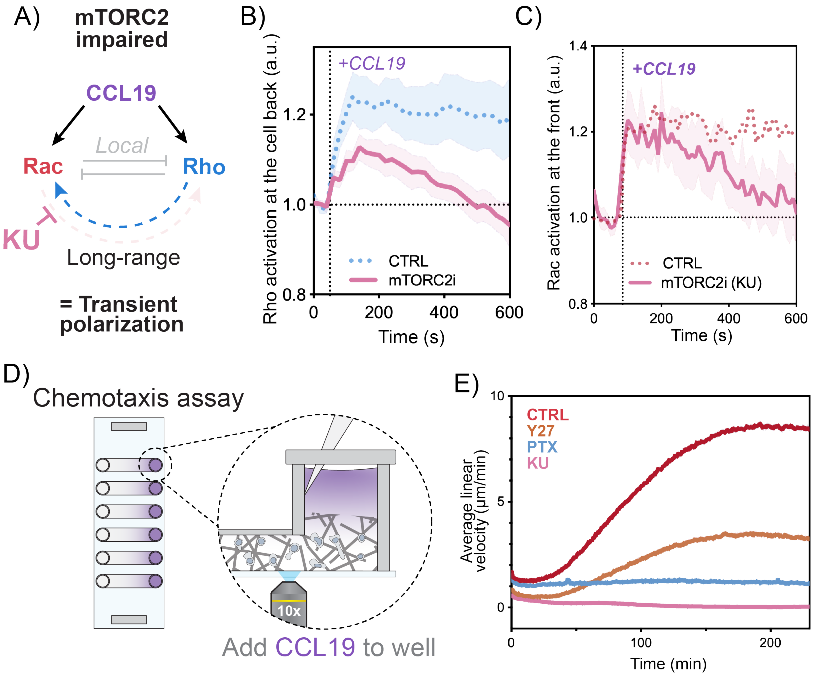

***Figure 8H, I, O, S10C, S11E*: <u>Impairing mTORC2 leads impairs CCL19-mediated long-lasting polarization and chemotaxis.</u>** *A) We prevent the long-range Rac to Rho communication using the mTORC2 inhibitor KU-0063794. B) C) Average time trace of Rho and Rac following addition of 25nM of CCL19 in cells treated with 10μM of KU-0063794 showing that cells with impaired mTORC2 signaling only polarize transiently in response to CCL19 (mean ±95%CI; n = 20, N= 2). D) Ex-vivo assay for human primary T cell chemotaxis. E) Average linear velocity of all tracks at each timepoint recorded. Control Volunteer N = 3, PTX Volunteer N = 3, Y27 Volunteer N = 2, KU Volunteer = 2 (mean ±95%CI).*

Minor points

A. The authors refer to RAC and RHO activity. However, the probes they use sense the activation of the GTPases and not their enzymatic activity. This should be changed to "activation" or "signalling activity".
Corrected.

B. Pak-PBD = Pak-Pak-binding domain. I assume the authors simply mean PBD or alternatively, Pak-CRIB.
We define Pak-PBD as: P21-Activated Kinase 1 P21-Binding Domain

C. In fig C and D of Fig 1, the increase in signal for the PBD probe is clearly increasing at the front, however, the loss in signal at the back is not evident in the representative image.
Corrected.

D. (E) (F) onward are wrongly written in the legend for Fig. 4.
Corrected.

24th February 2026

Reviewer #1 (Remarks to the Author):

The authors have satisfactorily addressed this reviewer's remarks. As such, our recommendation is: ACCEPT.

Reviewer #2 (Remarks to the Author):

The authors have performed a lot of work and responded to this reviewer's concerns satisfactorily.

We thank both reviewers for their careful evaluation and constructive feedback which has helped us significantly improve this manuscript.
